# Controlling the stereochemistry in 2-oxo-aldehyde-derived Ugi adducts through the cinchona alkaloid-promoted electrophilic fluorination

**DOI:** 10.3762/bjoc.16.163

**Published:** 2020-08-11

**Authors:** Yuqing Wang, Gaigai Wang, Anatoly A Peshkov, Ruwei Yao, Muhammad Hasan, Manzoor Zaman, Chao Liu, Stepan Kashtanov, Olga P Pereshivko, Vsevolod A Peshkov

**Affiliations:** 1 College of Chemistry, Chemical Engineering and Materials Science, Soochow University, Dushu Lake Campus, Suzhou, 215123, P.R. China; 2 Department of Chemistry, School of Science and Technology, Nazarbayev University, 53 Kabanbay Batyr Ave, Block 7, Nur-Sultan 010000, Republic of Kazakhstan; 3 Saint Petersburg State University, Saint Petersburg 199034, Russian Federation; 4 Department of Chemistry, Xi’an Jiaotong-Liverpool University, Suzhou, 215123, P.R. China; 5 The Environment and Resource Efficiency Cluster (EREC), Nazarbayev University, Nur-Sultan, Republic of Kazakhstan

**Keywords:** cinchona alkaloids, electrophilic fluorination, enantioselective synthesis, 2-oxo-aldehydes, Ugi reaction

## Abstract

In this report, we introduce a new strategy for controlling the stereochemistry in Ugi adducts. Instead of controlling stereochemistry directly during the Ugi reaction we have attempted to stereodefine the chiral center at the peptidyl position through the post-Ugi functionalization. In order to achieve this, we chose to study 2-oxo-aldehyde-derived Ugi adducts many of which partially or fully exist in the enol form that lacks the aforementioned chiral center. This in turn led to their increased nucleophilicity as compared to the standard Ugi adducts. As such, the stereocenter at the peptidyl position could be installed and stereodefined through the reaction with a suitable electrophile. Towards this end, we were able to deploy an asymmetric cinchona alkaloid-promoted electrophilic fluorination producing enantioenriched post-Ugi adducts fluorinated at the peptidyl position.

## Introduction

Multicomponent reactions (MCRs) [1–13] in general and isocyanide-based MCRs [14–18] in particular represent powerful tools of modern synthetic chemistry that allow to generate structurally diverse and complex products in a step and atom-economic manner from simple and accessible precursors. Three-component Passerini [19–22] and four-component Ugi [23–24] reactions that rely on the ability of organic isocyanides to participate in the nucleophilic attack onto the carbonyl or imine group are among the most studied MCRs. Accordingly, a wide range of post-MCR transformations have been elaborated allowing to upgrade the Passerini and Ugi adducts into potentially bioactive heterocycles [25–32]. While both Passerini and Ugi reactions proved to be quite robust towards different classes of substrates and possess broad functional group tolerance, the difficulties associated with controlling their stereochemical outcome [33–35] severely restrict their applicability in medicinal chemistry [36].

The typical Passerini three-component reaction (P-3CR) of a carboxylic acid **1**, an aldehyde **2**, and an isocyanide **3** being conducted in a polar-aprotic solvent such as THF or dichloromethane results in the assembly of an α-acyloxyamide adduct **4**. Just a few protocols for the asymmetric P-3CR [37–40] and its modifications [41–47] have been reported over the course of the last two decades (Table 1). In the majority of those protocols [37–39], the stereocontrol was achieved with the aid of different Lewis acid promoters bearing chiral ligands, while the most recent and at the same time the most general procedure developed by Liu, Tan and co-workers relied on the stereoinduction by a chiral phosphoric acid (Table 1) [40].

**Table 1:** Asymmetric protocols for Passerini three-component reaction (P-3CR).

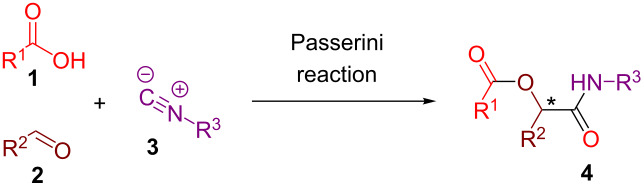				

Asymmetric protocol	Catalytic system	Conditions	Yield range, %	ee range, %

Dömling and co-workers, 2003 [37]	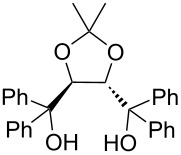 (1 equiv) Ti(OiPr)_4_ (1.35 equiv)	THF (0.5 M), overnight	12–48	32–42
Schreiber and co-workers, 2004 [38]	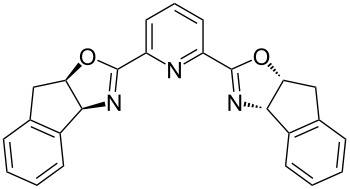 (20 mol %) Cu(OTf)_2_ (20 mol %)	DCM (0.04 M), molecular sieves, 18–48 h	75–98	62–98
Wang, Zhu and co-workers, 2008 [39]	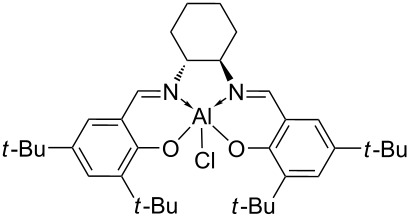 (10 mol %)	toluene (0.33 M), argon, −40 °C, 48 h	51–70	63 to >99
Liu, Tan and co-workers, 2015 [40]	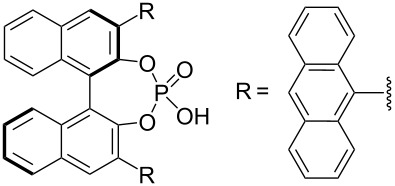 (10–20 mol %)	CHCl_3_ (0.033 M or 0.05 M), argon, MgSO_4_, rt or −20 °C or −35 °C, 36 h	41–99	84–99

In the standard Ugi four-component reaction (U-4CR) a carboxylic acid **1**, an aldehyde **2**, and an isocyanide **3** are complemented by a primary amine **5** that altogether undergo a condensation into a peptide-like adduct **6**. These reactions are typically conducted in polar protic solvents such as methanol or water. Several examples of diastereoselective U-4CR have been described [48–54]. Yet, the Ugi reaction turned out to be a rather challenging chemical transformation in terms of establishing the asymmetric version [33–35]. Apart from the two asymmetric three-component modifications lacking the acid component [55–56], no general asymmetric protocol for the four-component mode has been developed until very recently. In 2018, Houk, Tan and co-workers described an efficient enantioselective procedure operating in the presence of catalytic amounts of chiral phosphoric acids (Table 2) [57]. Two synthetic protocols have been elaborated allowing the engagement of different classes of substrates (Table 2). In addition, the group of Tan adapted their findings to the chiral phosphoric acid-catalyzed three-component Ugi reaction of an aldehyde **2**, an isocyanide **3**, and a primary amine **5** [58].

**Table 2:** Asymmetric protocols for Ugi four-component reaction (U-4CR) by Houk, Tan and co-workers.

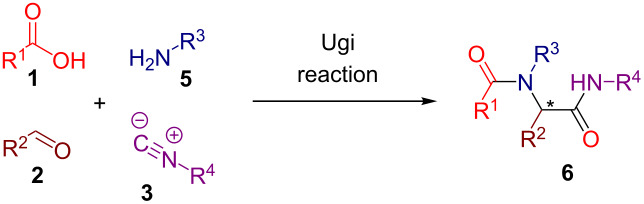				

Substrate scope	Catalytic system	Conditions	Yield range, %	ee range, %

R^2^ = Alk; R^3^ = Ar	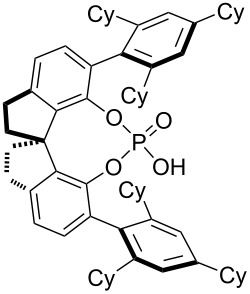 (5 mol %) Cy = cyclohexyl	DCM (0.05 M), argon, 5 Å MS, −20 °C, 12 h	43–96	75–97
R^2^ = Ar; R^3^ = Alk	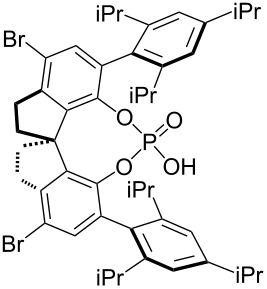 (10 mol %)	cyclohexane (0.025 M), argon, 5 Å MS, 20 °C, 36 h–7 d	60–96	80–94

**Scheme 1:**
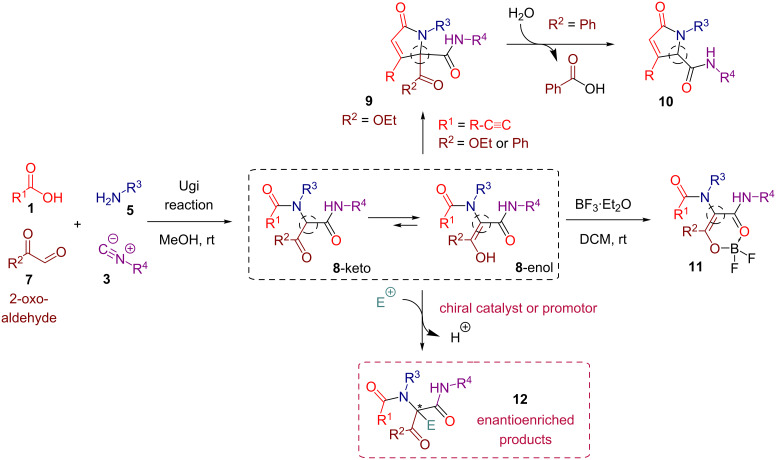
Post-transformations of 2-oxo-aldehyde-derived Ugi adducts **8**.

As a part of our ongoing research, we have been involved in the synthesis and utilization of 2-oxo-aldehyde-derived Ugi adducts **8** (Scheme 1). These products possess an increased nucleophilicity of the peptidyl position compared to the standard Ugi adducts owing to an additional electron-withdrawing group introduced with the 2-oxo-aldehyde **7**. For example, we have explored the potential of the peptidyl reactive site in a number of enolization-driven post-Ugi cyclizations leading to the assembly of pyrrol-2-ones **9** and **10** [59–60]. We have also taken advantage of a 1,3-dicarbonyl moiety present in **8** by engaging them in an enolization-triggered complexation with boron trifluoride diethyl etherate. The resulting *O*,*O*-chelated boron complexes **11** turned out to be strong solid-state emitters featuring clear aggregation-induced emission (AIE) characteristics [61].

Encouraged by these results, we decided to attempt the reaction of 2-oxo-aldehyde-derived Ugi adducts **8** with suitable electrophiles in the presence of chiral catalyst or promotor. In this way, the peptidyl position of **8** could be simultaneously functionalized and stereodefined leading to the formation of enantioenriched products **12** (Scheme 1). Towards this goal, we have previously tested the reactivity of **8** in the intermolecular aldol addition to ethyl glyoxalate [62]. As these attempts met with failure, we have turned our attention to enantioselective fluorination reactions [63–65]. Specifically, we decided to explore the enantioselective electrophilic fluorination of carbonyl compounds promoted by cinchona alkaloid derivatives developed independently by Shibata, Takeuchi and co-workers [65–66] and by the group of Cahard [67–70]. The method proved to be applicable to a broad range of substrates under a variety of conditions [71–78] and was successfully utilized for the synthesis of several bioactive molecules [79–81]. Herein we present its adaptation for the enantioselective fluorination of 2-oxo-aldehyde-derived Ugi adducts **8** leading to the installment of a fluorine-bound quaternary stereocenter at the peptidyl position. It should be stressed that the exploration of methods for the enantioselective fluorination continues to be an important topic considering the ever-growing role of fluorine derivatives in drug design and development [82–83]. An alternative strategy to prepare related quaternary carbon-containing adducts bearing fluorine and nitrogen atoms involves the asymmetric addition of α-fluoro-β-ketoesters or α-fluoro-α-nitro esters to appropriate electrophiles [84–89].

## Results and Discussion

**Scheme 2:**
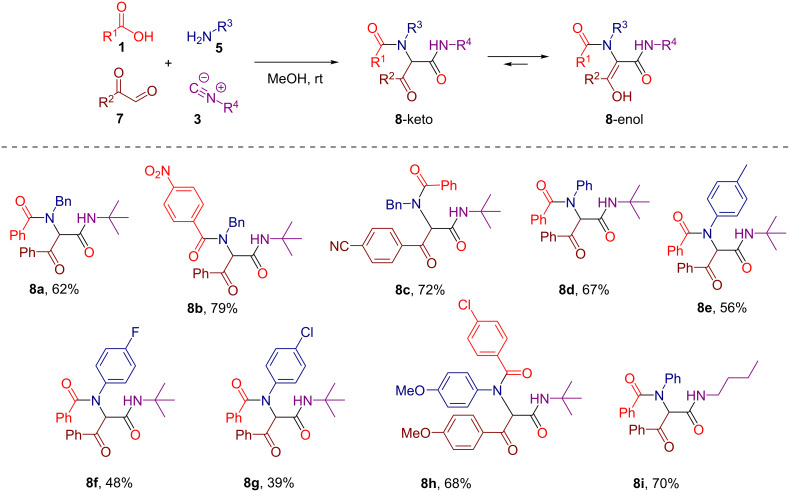
Synthesis of 2-oxo-aldehyde-derived Ugi adducts.

Initially, we prepared a series of Ugi adducts **8** by varying acid, 2-oxo-aldehyde, amine, and isocyanide components (Scheme 2). The product **8a** obtained from benzoic acid, phenylglyoxal, benzylamine, and *tert*-butyl isocyanide was selected for the screening of the reaction parameters (Table 3). The overall process was conducted in a two-step one-pot fashion as was originally designed by Shibata. In the first step, a cinchona alkaloid derivative was allowed to react with an electrophilic fluorinating agent to afford an *N*-fluoroammonium salt of the cinchona alkaloid via transfer fluorination. In the second step, the Ugi adduct **8a** was added to this in situ generated *N*-fluorocinchona intermediate to afford the desired fluorinated product **12a**. Thus, according to this synthetic and mechanistic path, the *N*-fluorocinchona intermediate acted as an asymmetric electrophilic fluorinating agent.

**Table 3:** Screening of the conditions for enantioselective fluorination of Ugi adduct **8a**.^a^

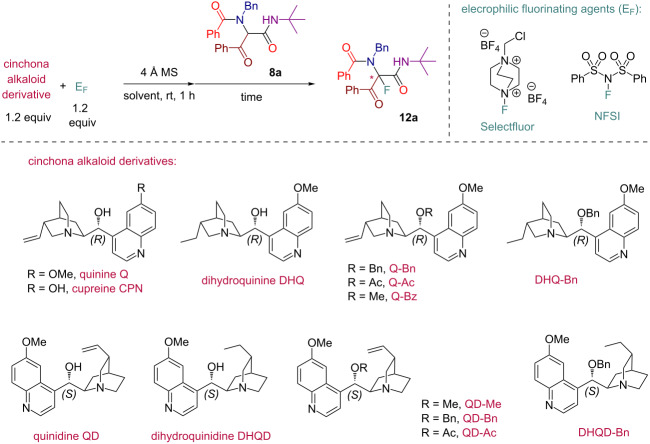						

Entry	Cinchona alkaloid derivative	Fluorinating agent	Solvent	Time, h	Yield,^b^ %	ee (major enantiomer),^c^ %

1	Q	Selectfluor	MeCN	23	88	19 (F)
2	Q	Selectfluor	THF	19	94	21 (F)
3	Q	Selectfluor	DCM	21	74	26 (F)
4^d^	Q	Selectfluor	DCM	70	42	20 (F)
5	Q	Selectfluor	CHCl_3_	21	71–79	31–44 (F)
6	CPN	Selectfluor	CHCl_3_	21	–^e^	–^e^
7	DHQ	Selectfluor	CHCl_3_	16-21	85–90	46–57 (F)
8	DHQ	Selectfluor	THF	12	76	43 (F)
9	Q-Bn	Selectfluor	CHCl_3_	21	78–83	39–41 (F)
10	Q-Ac	Selectfluor	CHCl_3_	21–24	35–59	42–51 (F)
11	Q-Bz	Selectfluor	CHCl_3_	20	62	32 (F)
12	DHQ-Bn	Selectfluor or NFSI	CHCl_3_	16–22	60–81	57–73 (F)
13	QD	Selectfluor or NFSI	CHCl_3_	21–23	83–94	51–69 (S)
14	DHQD	Selectfluor	CHCl_3_	21	86–92	15–21 (S)
15	QD-Me	Selectfluor	CHCl_3_	23	75	14 (S)
16	QD-Ac	Selectfluor	CHCl_3_	20	29	27 (S)
17	QD-Bn	Selectfluor	CHCl_3_	21	80	29 (S)
18	DHQD-Bn	Selectfluor	CHCl_3_	21	87	23 (S)

^a^The reactions were run on a 0.15–0.2 mmol scale; ^b^isolated yield; ^с^determined by chiral HPLC analysis, S: slower enantiomer, F: faster enantiomer; ^d^the reaction was conducted with 0.2 equiv of Q; ^e^no product **12a** was formed.

Conducting the fluorination of **8a** with a quinine (Q)/Selectfluor combination in different solvents revealed chloroform as a preferred reaction medium for this process with acetonitrile, tetrahydrofuran, and dichloromethane being less efficient in terms of ee of the obtained product **12a** (Table 3, entries 1–5). Using catalytic quantities of quinine led to a prolonged reaction time and diminished yield and ee of **12a** compared to the analogous reaction with stoichiometric amounts of quinine (Table 3, entry 4 versus entry 3). Thus, we focused on the exploration of a stoichiometric version considering that most of cinchona alkaloid derivatives are readily accessible and recoverable. The original Shibata’s protocol also relied on the use of stoichiometric amounts of the chiral promotor that was in an agreement with a two-step reaction mode requiring the initial formation of the asymmetric fluorinating agent. An attempt to use cupreine, a member of the cinchona family featuring free phenolic hydroxy group, met with failure producing no desired product **12a** (Table 3, entry 6). Using dihydroquinine (DHQ) led to an improved ee of **12a** compared to the analogous reactions with quinine (Table 3, entries 7 and 8 versus entries 5 and 2, respectively). Testing ether and ester derivatives of Q and DHQ (Table 3, entries 9–12) led to further improvement allowing to obtain **12a** with up to 73% ee through the reaction with dihydroquinine benzyl ether (DHQ-Bn, Table 3, entry 12). Switching to quinidine (QD), dihydroquinidine (DHQD), and their derivatives allowed to alter the stereoselectivity of the process leading to the preferred formation of another enantiomer of **12a** (Table 3, entries 13–18) with the QD-promoted reaction affording the best ee value (Table 3, entry 13). Additionally, we tested *N*-fluorobenzenesulfonimide (NFSI) as an alternative fluorinating agent in a combination with DHQ-Bn and QD with the outcome being indistinguishable with the analogous reactions with Selectfluor.

Having these results in hand, we moved to investigating the scope and limitation of this procedure with the Ugi adducts **8** obtained previously (Table 4). In order to have a more balanced representation we decided to test most of these substrates **8** with eight different cinchona alkaloid derivatives including Q, DHQ, QD, DHQD, and their benzyl ether derivatives. To assure reproducibility, some of these reactions were repeated two to five times. In all such cases a range for the yield and ee value is reported in Table 4. Testing Ugi adducts **8b** and **8c** that similarly to **8a** were obtained from benzylamine and *tert*-butyl isocyanide by varying either acid or 2-oxo-aldehyde components revealed that it was difficult to achieve high enantioselectivity for such substrates. Specifically, none of the studied chiral promoters could afford the desired products **12b** and **12c** with the ee value consistently higher than 50%. Turning to the substrates **8d–g** derived from aromatic amines we were able to achieve improved enantioselectivity. The best results were obtained with Q-Bn, DHQ-Bn, DHQD, and QD-Bn that performed consistently well with each of the substrates **8d**–**g** delivering the corresponding fluorinated products **12d**–**g** with the ee values higher than 50% and in some cases close or even over 70%. Furthermore, it was possible to divert the selectivity of these reactions towards the preferred formation of either of the enantiomers by choosing an appropriate chiral promotor. Testing Ugi adduct **8h** allowed us to highlight the possibility of simultaneous variation of acid, 2-oxo-aldehyde, and amine components. Overall, four cinchona derivatives were screened with **8h** producing the fluorinated product **12h** with moderate to good enantioselectivity, which was consistent with the performance of other arylamine-derived Ugi adducts **8d**–**g**. The last investigated Ugi adduct **8i** featured the variation of the isocyanide component. Conducting fluorinations with this *n*-butyl isocyanide-derived Ugi adduct **8i**, the desired product **12i** could be successfully obtained. However, the degree of enantioselective induction was lower than in the reactions with the analogous *tert*-butyl isocyanide-derived substrate **8d**. This could probably be ascribed to a reduced rigidity and bulkiness of the *n*-butyl group as compared to *tert*-butyl.

**Table 4:** Scope and limitations of the asymmetric electrophilic fluorination of 2-oxo-aldehyde-derived Ugi adducts **8**.^a^

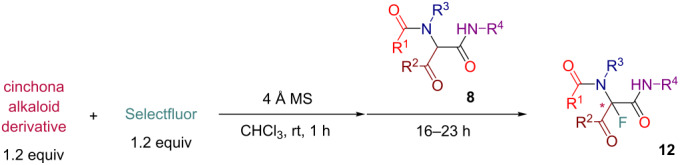									

		Q	DHQ	Q-Bn	DHQ‑Bn	QD	DHQD	QD-Bn	DHQD-Bn

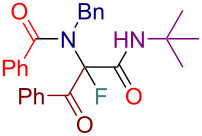 **12a**	yield,^b^ %	71–79	85–90	78–83	60–81	83–94	86–92	80	87
	ee,^c^ %	31–44 (F)	46–57 (F)	39–41 (F)	57–73 (F)	51–69 (S)	15–21 (S)	29 (S)	23 (S)

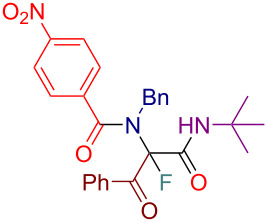 **12b**	yield,^b^ %	78–85	84–97	79	76–90	83–89	95–97	67–80	81
	ee,^c^ %	23–34 (S)	29–39 (S)	39 (S)	38–51 (S)	24–28 (F)	9–15 (F)	20–24 (F)	21 (F)

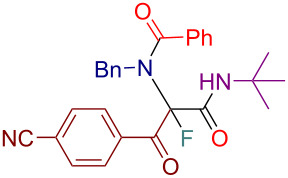 **12c**	yield,^b^ %	59–81	60–81	83	74–89	68–85	72–78	81	78
	ee,^c^ %	39–46 (S)	39–47 (S)	35 (S)	36 (S)	30–43 (F)	23–25 (F)	25 (F)	23 (F)

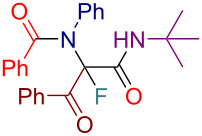 **12d**	yield,^b^ %	91	81	78	96	71–91	84	85	82
	ee,^c^ %	24 (F)	22 (F)	66 (S)	71 (S)	47–60 (S)	57 (S)	59 (F)	38 (F)

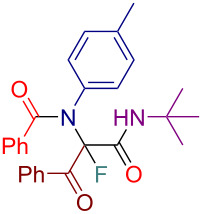 **12e**	yield,^b^ %	68–78	91–97	78–82	86–97	89–98	84–91	95–98	85
	ee,^c^ %	18–30 (F)	19–23 (F)	65–69 (S)	71–74 (S)	49–50 (S)	58–65 (S)	61–67 (F)	42 (F)

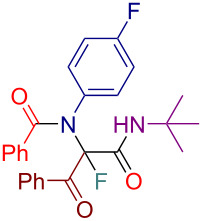 **12f**	yield,^b^ %	88–89	91–92	84–88	85–86	71–78	89–98	89	79
	ee,^c^ %	23–27 (F)	18–19 (F)	66–67 (S)	69-72 (S)	50–55 (S)	53–64 (S)	57–58 (F)	40 (F)

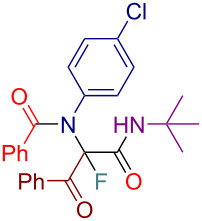 **12g**	yield,^b^ %	74–85	90	82–84	86–87	82–88	75–76	75–84	78–81
	ee,^c^ %	25–37 (F)	18–24 (F)	65–66 (S)	63–70 (S)	48–59 (S)	51–61 (S)	59–60 (F)	37–54 (F)

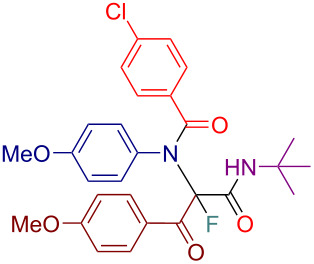 **12h**	yield,^b^ %	–^d^	–^d^	64	86	84	–^d^	75	–^d^
	ee,^c^ %	–^d^	–^d^	71 (F)	73 (F)	54 (F)	–^d^	55 (S)	–^d^

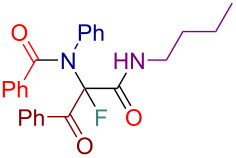 **12i**	yield,^b^ %	–^d^	–^d^	51	54	67	–^d^	49	–^d^
	ee,^c^ %	–^d^	–^d^	44 (F)	48 (F)	55 (F)	–^d^	29 (S)	–^d^

^a^The reactions were run on a 0.15–0.2 mmol scale; ^b^isolated yield; ^с^determined by chiral HPLC analysis, S: slower enantiomer, F: faster enantiomer; ^d^not conducted.

For the substrates **8a**–**g** tested with eight cinchona alkaloid derivatives fluorinations with QD and DHQD led to the reversed stereoselectivity as compared to the reactions with Q and DHQ. For the benzylamine-derived substrates **8a**–**c** benzyl ether derivatives of cinchona alkaloids favored the formation of the same enantiomer of **12** as their free alcohol counterparts (e.g., both Q-Bn and Q favored the formation of the same enantiomer of **12**). In contrast, for the substrates **8d**–**g** derived from aromatic amines a reversed trend was observed. In case of **8d**–**g**, benzyl ether derivatives of cinchona alkaloids favored the formation of the opposite enantiomer of **12** as compared to the one favored by the parent alkaloids featuring free alcohol moiety (e.g., Q-Bn favored the formation of the enantiomer of **12** with the absolute configuration opposite to the one favored by Q). Substrates **8h** and **8i** were tested with only four cinchona alkaloid derivatives, but the observed stereochemical trends appeared to be consistent with those established for the rest of arylamine-derived Ugi adducts **8d**–**g**.

**Figure 1:**
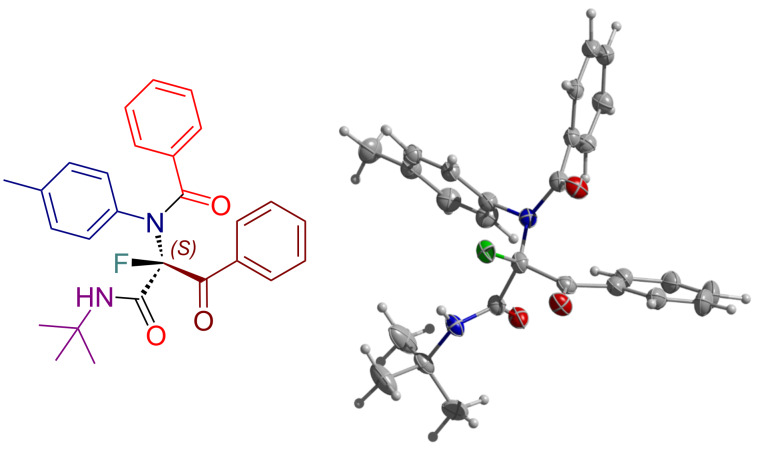
Molecular representation of the X-ray crystal structure of (*S*)-**12e** (slow enantiomer).

Finally, we attempted to determine the absolute configuration of a stereocenter for the major enantiomer of the representative fluorinated product. Using a sample of product **12e** with the ee value of 74% we were able to obtain crystals suitable for the structure determination via X-ray crystallographic analysis. It was found that a part of the substance crystallized in a racemic form while the rest crystallized in an enantiopure form. By resolving both types of crystals [90], the configuration of the major slow enantiomer was successfully established as *S* (Figure 1).

## Conclusion

In summary, we have explored a new strategy for controlling the stereochemistry in the Ugi adducts through the enantioselective post-Ugi functionalization. Specifically, we have adapted the cinchona alkaloid-promoted asymmetric electrophilic fluorination for derivatizing the 2-oxo-aldehyde-derived Ugi adducts. This allowed us to obtain the post-Ugi products fluorinated at the peptidyl position with the enantiomeric excess values in several instances reaching more than 70%.

## Supporting Information

File 1 Experimental procedures, characterization data and copies of spectra.

File 2 Crystallographic data (CIF) for compound (*S*)-**12e**.

File 3 Crystallographic data (CIF) for compound (rac)-**12e**.

## References

[1] Bienaymé H, Hulme C, Oddon G, Schmitt P (2000). Chem – Eur J.

[2] Zhu J, Bienaymé H (2005). Multicomponent Reactions.

[3] Sunderhaus J D, Martin S F (2009). Chem – Eur J.

[4] Ganem B (2009). Acc Chem Res.

[5] Orru R V A, Ruijter E (2010). Synthesis of Heterocycles via Multicomponent Reactions I.

[6] Orru R V A, Ruijter E (2010). Synthesis of Heterocycles via Multicomponent Reactions II.

[7] Jiang B, Rajale T, Wever W, Tu S-J, Li G (2010). Chem – Asian J.

[8] Dömling A, Wang W, Wang K (2012). Chem Rev.

[9] Zhu J, Wang Q, Wang M-X (2014). Multicomponent Reactions in Organic Synthesis.

[10] Banfi L, Basso A, Moni L, Riva R (2014). Eur J Org Chem.

[11] Cioc R C, Ruijter E, Orru R V A (2014). Green Chem.

[12] Levi L, Müller T J J (2016). Chem Soc Rev.

[13] Murlykina M V, Morozova A D, Zviagin I M, Sakhno Y I, Desenko S M, Chebanov V A (2018). Front Chem (Lausanne, Switz).

[14] Dömling A, Ugi I (2000). Angew Chem, Int Ed.

[15] Akritopoulou-Zanze I (2008). Curr Opin Chem Biol.

[16] Koopmanschap G, Ruijter E, Orru R V A (2014). Beilstein J Org Chem.

[17] Váradi A, Palmer T C, Notis Dardashti R, Majumdar S (2016). Molecules.

[18] Bode M L, Gravestock D, Rousseau A L (2016). Org Prep Proced Int.

[19] Passerini M (1921). Gazz Chim Ital.

[20] Banfi L, Riva R (2005). Org React.

[21] Kazemizadeh A R, Ramazani A (2012). Curr Org Chem.

[22] Chandgude A (2017). Multicomponent Reactions: Development, Scope, and Applications.

[23] Ugi I, Meyr R, Fetzer U, Steinbrückner C (1959). Angew Chem.

[24] Ugi I, Steinbrückner C (1960). Angew Chem.

[25] Banfi L, Basso A, Riva R, Orru R V A, Ruijter E (2010). Synthesis of Heterocycles Through Classical Ugi and Passerini Reactions Followed by Secondary Transformations Involving One or Two Additional Functional Groups. Synthesis of Heterocycles via Multicomponent Reactions I.

[26] Sharma U K, Sharma N, Vachhani D D, Van der Eycken E V (2015). Chem Soc Rev.

[27] Zhang Z, Zheng X, Guo C (2016). Chin J Org Chem.

[28] Li X, Jia X, Yin L (2017). Chin J Org Chem.

[29] Banfi L, Basso A, Lambruschini C, Moni L, Riva R (2017). Chem Heterocycl Compd.

[30] Heravi M M, Mohammadkhani L (2020). Synthesis of various N-heterocycles using the four-component Ugi reaction. Advances in Heterocyclic Chemistry.

[31] Bariwal J, Kaur R, Voskressensky L G, Van der Eycken E V (2018). Front Chem (Lausanne, Switz).

[32] Mohammadi-Khanaposhtani M, Jalalimanesh N, Saeedi M, Larijani B, Mahdavi M (2020). Mol Diversity.

[33] Wang Q, Wang D-X, Wang M-X, Zhu J (2018). Acc Chem Res.

[34] Shaabani S, Dömling A (2018). Angew Chem, Int Ed.

[35] Riva R (2018). Science.

[36] Huang Y, Wolf S, Koes D, Popowicz G M, Camacho C J, Holak T A, Dömling A (2012). ChemMedChem.

[37] Kusebauch U, Beck B, Messer K, Herdtweck E, Dömling A (2003). Org Lett.

[38] Andreana P R, Liu C C, Schreiber S L (2004). Org Lett.

[39] Wang S-X, Wang M-X, Wang D-X, Zhu J (2008). Angew Chem, Int Ed.

[40] Zhang J, Lin S-X, Cheng D-J, Liu X-Y, Tan B (2015). J Am Chem Soc.

[41] Denmark S E, Fan Y (2005). J Org Chem.

[42] Wang S, Wang M-X, Wang D-X, Zhu J (2007). Eur J Org Chem.

[43] Wang S-X, Wang M-X, Wang D-X, Zhu J (2007). Org Lett.

[44] Yue T, Wang M-X, Wang D-X, Zhu J (2008). Angew Chem, Int Ed.

[45] Yue T, Wang M-X, Wang D-X, Masson G, Zhu J (2009). J Org Chem.

[46] Mihara H, Xu Y, Shepherd N E, Matsunaga S, Shibasaki M (2009). J Am Chem Soc.

[47] Zeng X, Ye K, Lu M, Chua P J, Tan B, Zhong G (2010). Org Lett.

[48] Gulevich A V, Balenkova E S, Nenajdenko V G (2007). J Org Chem.

[49] Nenajdenko V G, Gulevich A V, Chernichenko K Y, Sokolova N V, Balenkova E S (2011). Mendeleev Commun.

[50] Banfi L, Basso A, Cerulli V, Rocca V, Riva R (2011). Beilstein J Org Chem.

[51] Banfi L, Basso A, Chiappe C, De Moliner F, Riva R, Sonaglia L (2012). Org Biomol Chem.

[52] Zhu D, Xia L, Pan L, Li S, Chen R, Mou Y, Chen X (2012). J Org Chem.

[53] Caputo S, Basso A, Moni L, Riva R, Rocca V, Banfi L (2016). Beilstein J Org Chem.

[54] Lambruschini C, Moni L, Banfi L (2020). Eur J Org Chem.

[55] Hashimoto T, Kimura H, Kawamata Y, Maruoka K (2012). Angew Chem, Int Ed.

[56] Zhao W, Huang L, Guan Y, Wulff W D (2014). Angew Chem, Int Ed.

[57] Zhang J, Yu P, Li S-Y, Sun H, Xiang S-H, Wang J, Houk K N, Tan B (2018). Science.

[58] Zhang J, Wang Y-Y, Sun H, Li S-Y, Xiang S-H, Tan B (2020). Sci China: Chem.

[59] Nechaev A A, Peshkov A A, Peshkov V A, Van der Eycken E V (2016). Synthesis.

[60] Peshkov A A, Peshkov V A, Li Z, Pereshivko O P, Van der Eycken E V (2014). Eur J Org Chem.

[61] Wei H, Wang G, Wang Y, Li B, Huang J, Kashtanov S, Van Hecke K, Pereshivko O P, Peshkov V A (2017). Chem – Asian J.

[62] Hasan M, Zaman M, Peshkov A A, Amire N, Les A, Nechaev A A, Wang Y, Kashtanov S, Van der Eycken E V, Pereshivko O P (2020). Eur J Org Chem.

[63] Shibata N, Ishimaru T, Nakamura S, Toru T (2007). J Fluorine Chem.

[64] Lectard S, Hamashima Y, Sodeoka M (2010). Adv Synth Catal.

[65] Shibatomi K, Narayama A, Soga Y, Muto T, Iwasa S (2011). Org Lett.

[66] Shibata N, Suzuki E, Takeuchi Y (2000). J Am Chem Soc.

[67] Shibata N, Suzuki E, Asahi T, Shiro M (2001). J Am Chem Soc.

[68] Cahard D, Audouard C, Plaquevent J-C, Roques N (2000). Org Lett.

[69] Cahard D, Audouard C, Plaquevent J-C, Toupet L, Roques N (2001). Tetrahedron Lett.

[70] Mohar B, Baudoux J, Plaquevent J-C, Cahard D (2001). Angew Chem, Int Ed.

[71] Baudequin C, Plaquevent J-C, Audouard C, Cahard D (2002). Green Chem.

[72] Baudequin C, Loubassou J-F, Plaquevent J-C, Cahard D (2003). J Fluorine Chem.

[73] Thierry B, Audouard C, Plaquevent J-C, Cahard D (2004). Synlett.

[74] Mohar B, Sterk D, Ferron L, Cahard D (2005). Tetrahedron Lett.

[75] Fukuzumi T, Shibata N, Sugiura M, Nakamura S, Toru T (2006). J Fluorine Chem.

[76] Ramírez J, Huber D P, Togni A (2007). Synlett.

[77] Yi W-B, Huang X, Zhang Z, Zhu D-R, Cai C, Zhang W (2012). Beilstein J Org Chem.

[78] Wang M, Wang B M, Shi L, Tu Y Q, Fan C-A, Wang S H, Hu X D, Zhang S Y (2005). Chem Commun.

[79] Shibata N, Ishimaru T, Suzuki E, Kirk K L (2003). J Org Chem.

[80] Zoute L, Audouard C, Plaquevent J-C, Cahard D (2003). Org Biomol Chem.

[81] Shibata N, Ishimaru T, Nakamura M, Toru T (2004). Synlett.

[82] Gillis E P, Eastman K J, Hill M D, Donnelly D J, Meanwell N A (2015). J Med Chem.

[83] Inoue M, Sumii Y, Shibata N (2020). ACS Omega.

[84] Huber D P, Stanek K, Togni A (2006). Tetrahedron: Asymmetry.

[85] Mang J-Y, Kim D-Y (2008). Bull Korean Chem Soc.

[86] Mang J Y, Kwon D G, Kim D Y (2009). J Fluorine Chem.

[87] Han X, Zhong F, Lu Y (2010). Adv Synth Catal.

[88] Cui H-F, Li P, Wang X-W, Chai Z, Yang Y-Q, Cai Y-P, Zhu S-Z, Zhao G (2011). Tetrahedron.

[89] Kwiatkowski J, Lu Y (2015). Org Biomol Chem.

[R90] 90Crystallographic data for rac-**12e** (CCDC 2015756) and (S)-**12e** (CCDC 2015755) have been deposited with the Cambridge Crystallographic Data Centre.

