# Supplementary material for: Controlling the stereochemistry in 2-oxo-aldehyde-derived Ugi adducts through the cinchona alkaloid-promoted electrophilic fluorination
Source: Beilstein J Org Chem. 2020 Aug 11;16:1963–73. doi: 10.3762/bjoc.16.163 (PMC7431756; doi:10.3762/bjoc.16.163)
Supplement: File 1 — Experimental procedures, characterization data and copies of spectra. [file Beilstein_J_Org_Chem-16-1963-s001.pdf]

BEILSTEIN JOURNAL OF ORGANIC CHEMISTRY

Supporting Information

for

Controlling the stereochemistry in 2-oxo-aldehyde-derived Ugi adducts through the cinchona alkaloid-promoted electrophilic fluorination

Yuqing Wang, Gaigai Wang, Anatoly A. Peshkov, Ruwei Yao, Muhammad Hasan, Manzoor Zaman, Chao Liu, Stepan Kashtanov, Olga P. Pereshivko and Vsevolod A. Peshkov

Beilstein J. Org. Chem. **2020,** *16,* 1963–1973. doi:10.3762/bjoc.16.163

Experimental procedures, characterization data and copies of spectra

License and Terms: This is a supporting information file under the terms of the Creative Commons Attribution License (http://creativecommons.org/licenses/by/4.0). Please note that the reuse, redistribution and reproduction in particular requires that the authors and source are credited.
The license is subject to the *Beilstein Journal of Organic Chemistry* terms and conditions: (https://www.beilstein-journals.org/bjoc)

Table of contents

X-ray crystallographic analysis	S2
Synthesis and characterization	S10
General information	S10
General procedure for the synthesis of Ugi adducts **8**	S11
General procedure for cinchona alkaloid-promoted electrophilic fluorination of Ugi adduct **8**	S15
Copies of ^{1}H, ^{13}C and ^{19}F NMR spectra	S27–S69

X-ray crystallographic analysis

General details

Single crystals were obtained from the sample of product **12e** with the ee value of 74%. Slow evaporation from ethyl acetate/*n*-hexane 1:20 yielded two types of crystals. A part of the substance was crystallized in a racemic form (rac-**12e**) while the rest was crystallized in an enantiopure form ((*S*)-**12e**).

HPLC traces of the original sample

ee = 74 (slower enantiomer), chiral promotor is DHQ-Bn

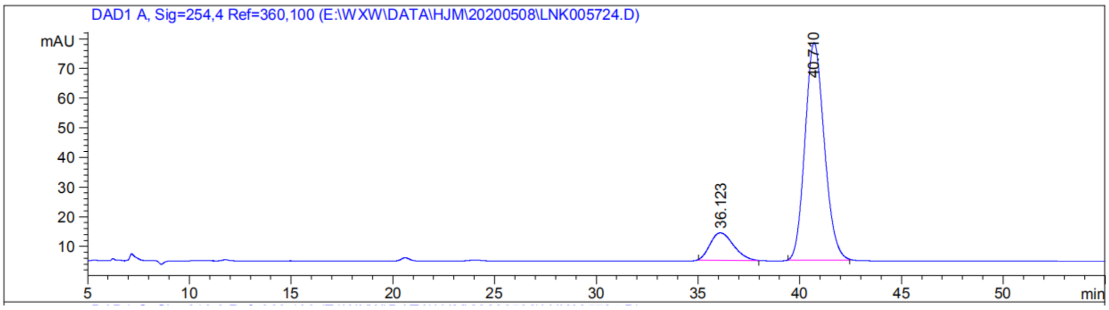

Peak #	RetTime [min]	Type	Width [min]	Area [mAU*s]	Height [mAU]	Area %
1	36.123	BB	0.9524	736.17999	9.19173	13.1752
2	40.710	BB	1.0203	4851.42139	73.28774	86.8248

HPLC traces of (*S*)-**12e** (slower enantiomer)

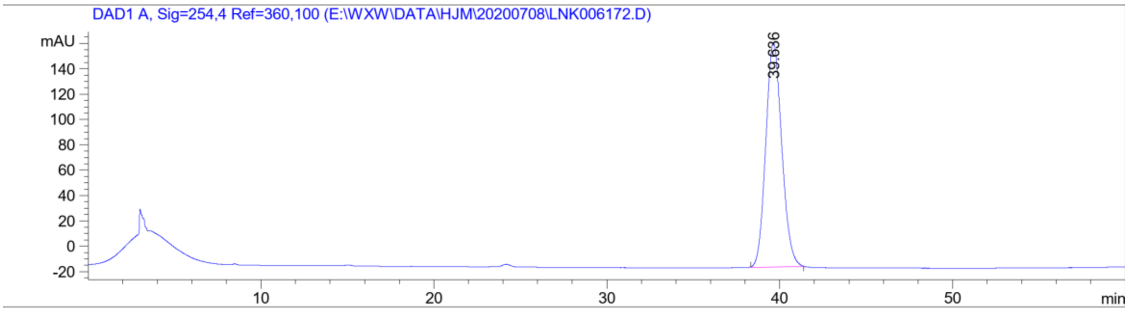

The images were interpreted and integrated with the program Diamond v.4.0 (Crystal Impact) [1]. Using

1. Diamond - Crystal and Molecular Structure Visualization Crystal Impact - Dr. H. Putz & Dr. K. Brandenburg GbR, Kreuzherrenstr. 102, 53227 Bonn, Germany http://www.crystalimpact.com/diamond.

Olex2 [2], the structures were solved with the ShelXS [3] structure solution program using direct methods, and refined by full-matrix least-squares on F^2 with the ShelXL [4] refinement package.

Crystal data for rac-12e.

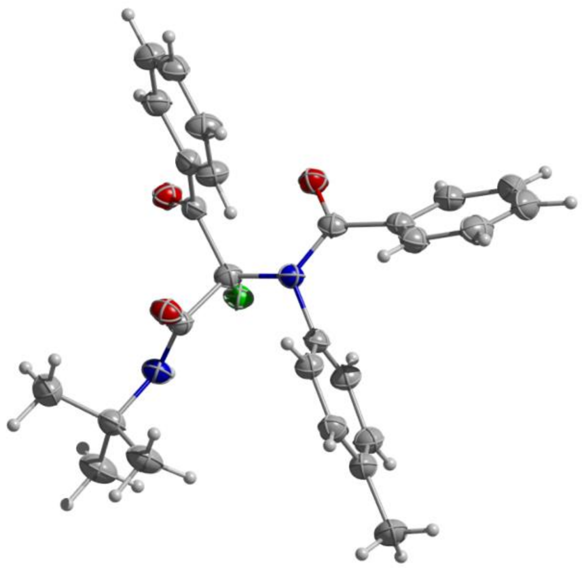

Molecular structure of rac-**12e**, showing thermal displacement ellipsoids at the 50% probability level

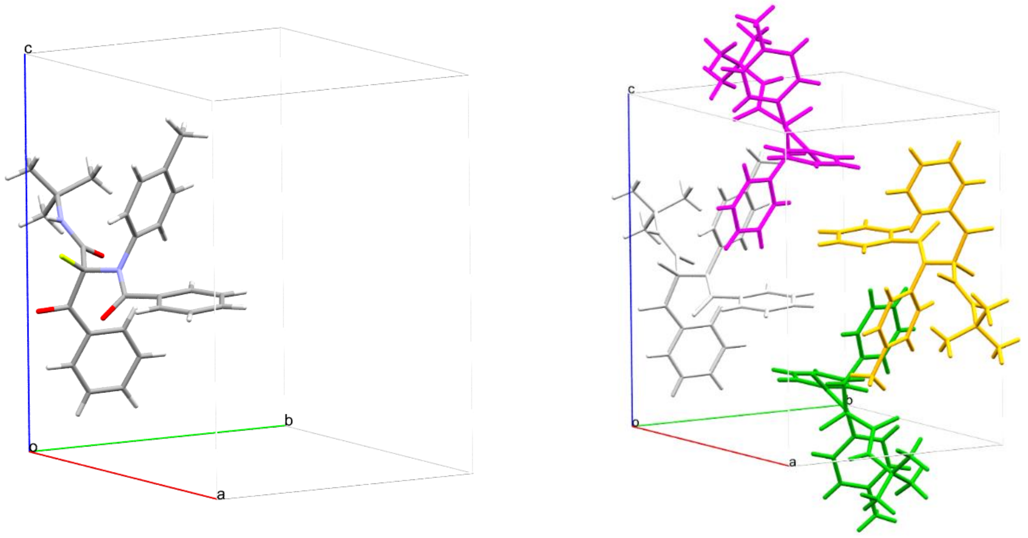

Composition of the unit cell of rac-**12e.** Asymmetric unit (left). Packing within the unit cell (right; colors: asymmetric unit (grey), molecules created by symmetry operations (green, yellow and magenta))

2. Dolomanov, O. V.; Bourhis, L. J.; Gildea, R. J.; Howard, J. A. K.; Puschmann, H. *J. Appl. Cryst.* **2009,** *42,* 339–341.

3. Sheldrick, G. M. *Acta Cryst. A* **2008,** *64,* 112–122.

4. Sheldrick, G. M. *Acta Cryst. C* **2015,** *71,* 3–8.

Crystal data for rac-12e. $C_{27}H_{27}FN_2O_3$ (M = 446.50 g/mol): monoclinic, space group $P2_1/n$ (no. 14), a = 13.199(2) Å, b = 11.712(2) Å, c = 15.424(3) Å, V = 2383.5(7) Å^3, Z = 4, T = 120.0 K, μ(CuKα) = 0.705 mm^{-1}, D*calc* = 1.244 g/cm^3, 31661 reflections measured (9.482 ≤ 2Θ ≤ 137.564), 4366 unique (R_{int} = 0.1095, R_{sigma} = 0.0530) which were used in all calculations. The final R_1 was 0.0637 (I > 2σ(I)) and wR_2 was 0.1783 (all data).

Structural features:

- chirality: (*S*/*R*) both enantiomers are present in the unit cell

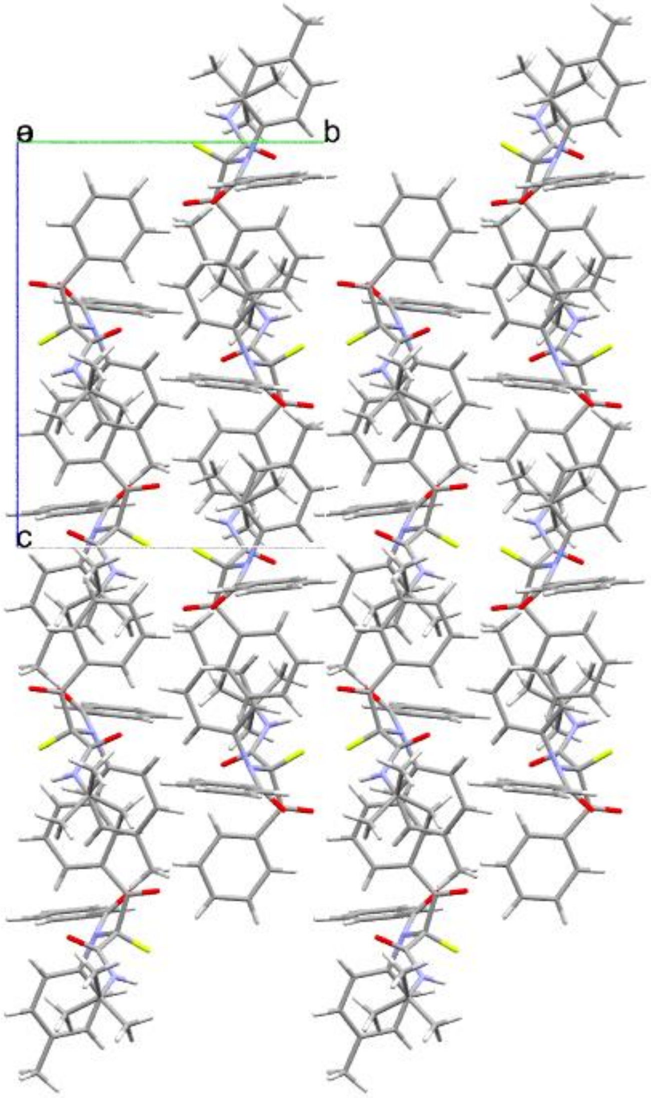

Packing diagram of the crystal structure of rac-**12e**, viewed down the crystallographic *a*-axis.

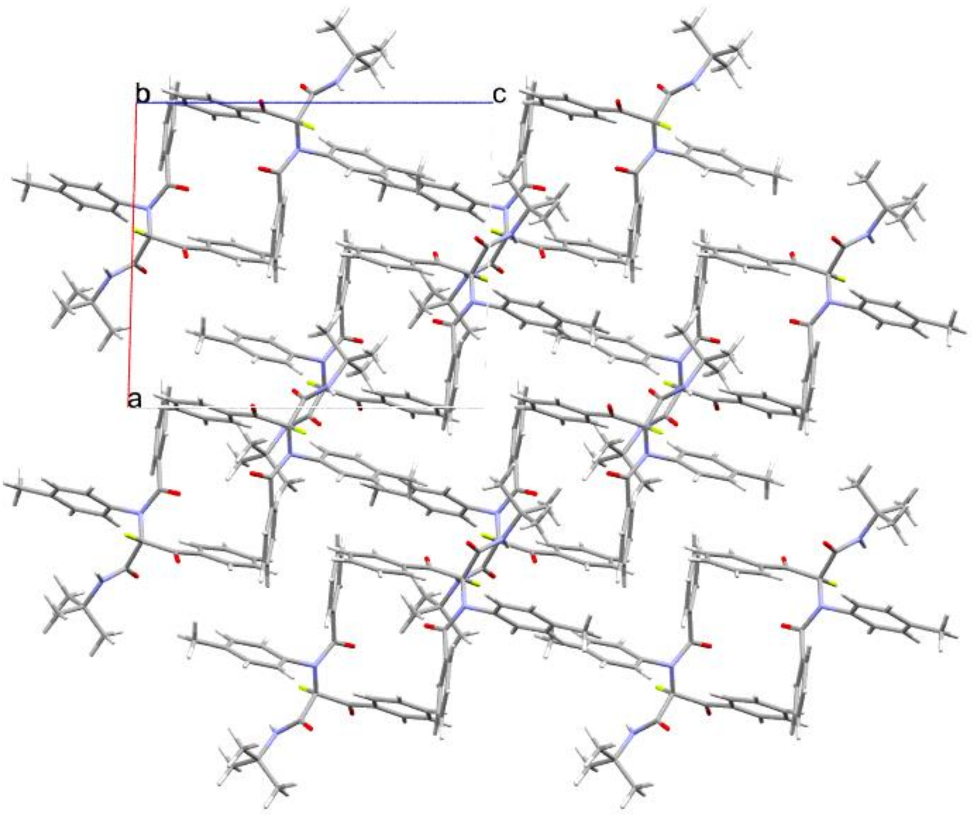

Packing diagram of the crystal structure of rac-**12e**, viewed down the crystallographic *b*-axis.

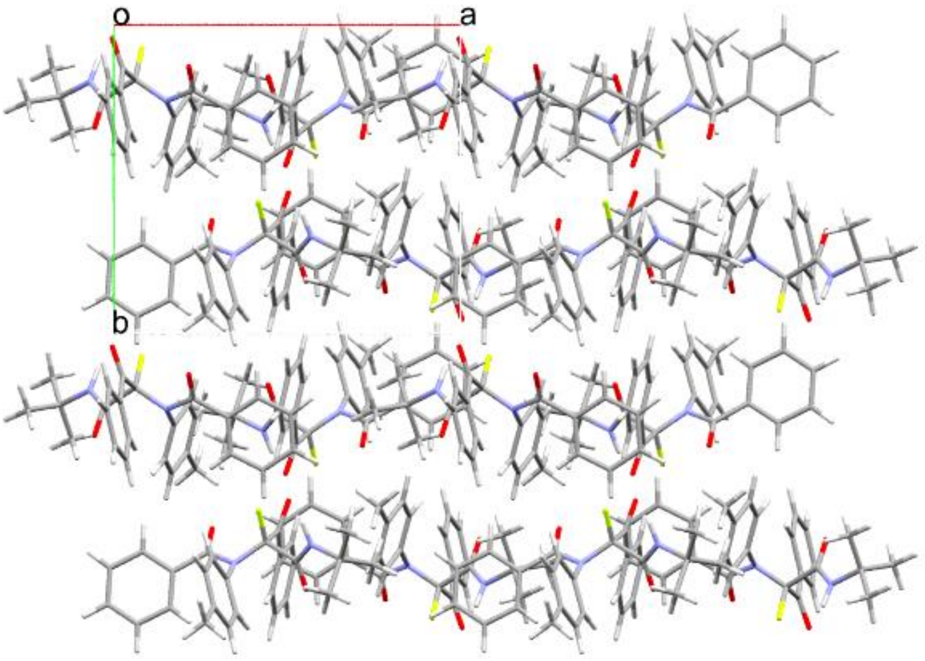

Packing diagram of the crystal structure of rac-**12e**, viewed down the crystallographic *c*-axis.

Crystal data for (*S*)-12e.

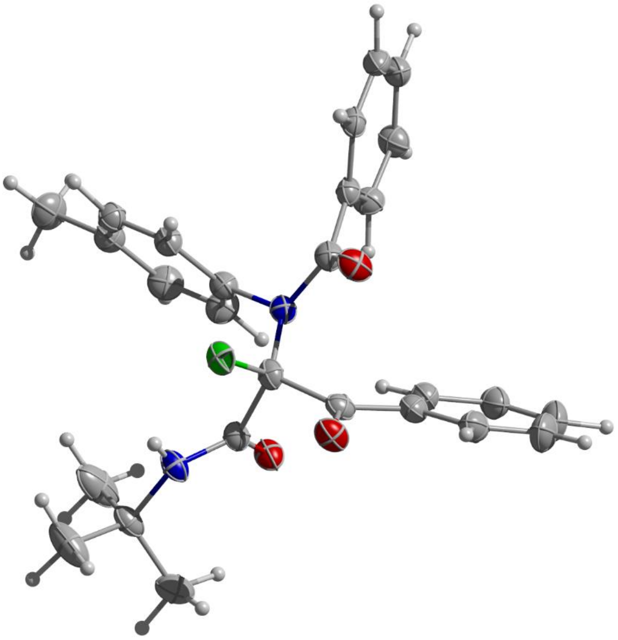

Molecular structure of (*S*)-**12e**, showing thermal displacement ellipsoids at the 50% probability level

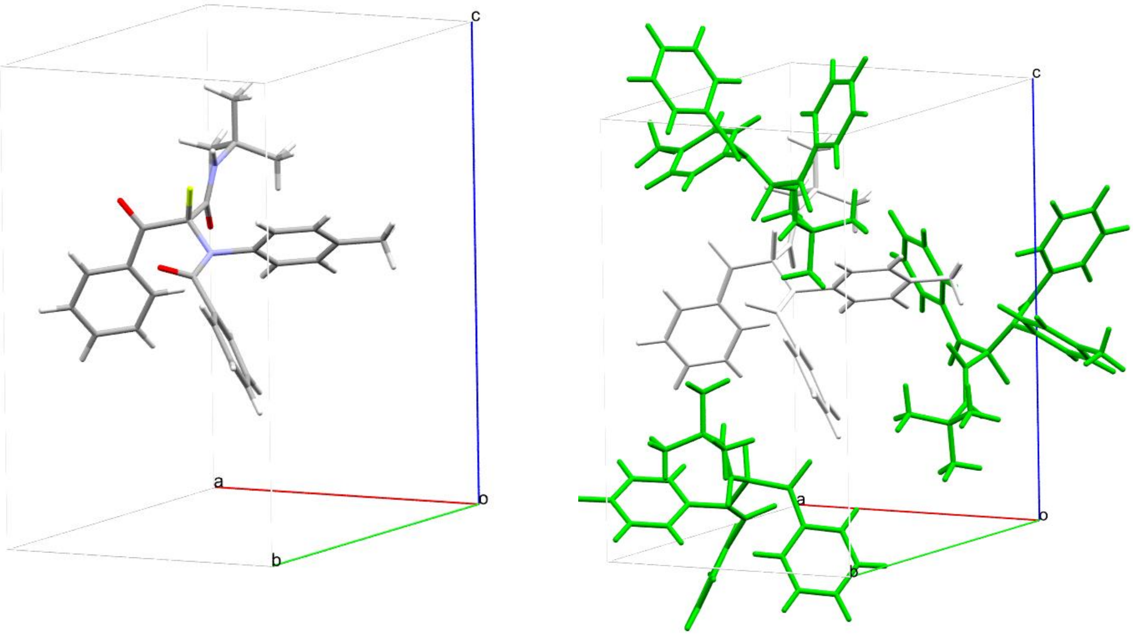

Composition if the unit cell of (*S*)-**12e.** Asymmetric unit (left). Packing within the unit cell (right; colors: asymmetric unit (grey), molecules created by symmetry operations (green))

Crystal data for (S)-12e. $C_{27}H_{27}FN_2O_3$ (M = 44.65 g/mol): orthorhombic, space group $P2_12_12_1$ (no. 19), a = 10.0069(3) Å, b = 14.7854(5) Å, c = 16.3253(5) Å, V = 2415.43(13) Å^3, Z = 40, T = 120.0 K, μ(CuKα) = 0.696 mm^{-1}, D$calc$ = 1.228 g/cm^3, 97609 reflections measured (8.068 ≤ 2Θ ≤ 136.662), 4430 unique (R_{int} = 0.0666, R_{sigma} = 0.0181) which were used in all calculations. The final R_1 was 0.0334 (I > 2σ(I)) and wR_2 was 0.0883 (all data).

Structural features:

- chirality: C00O (S)

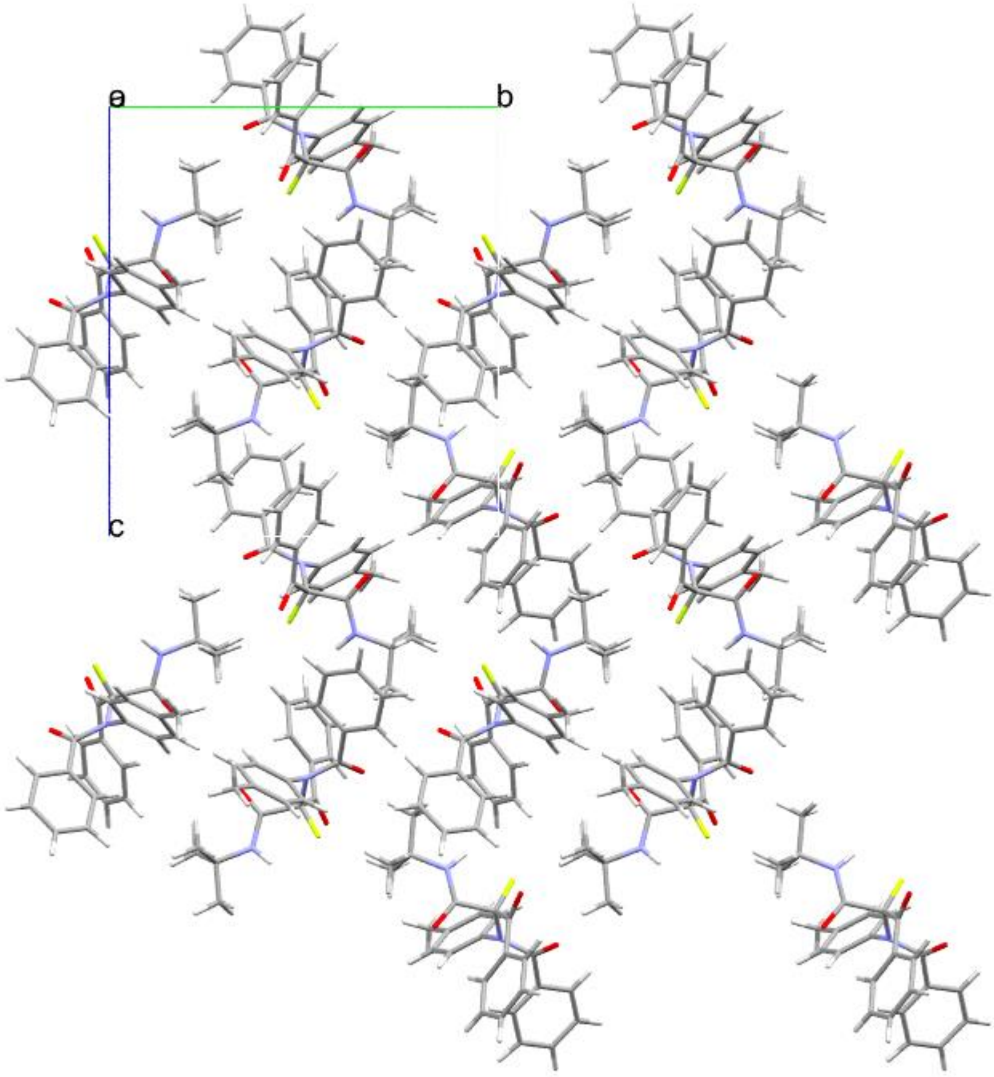

Packing diagram of the crystal structure of (*S*)-**12e**, viewed down the crystallographic *a*-axis.

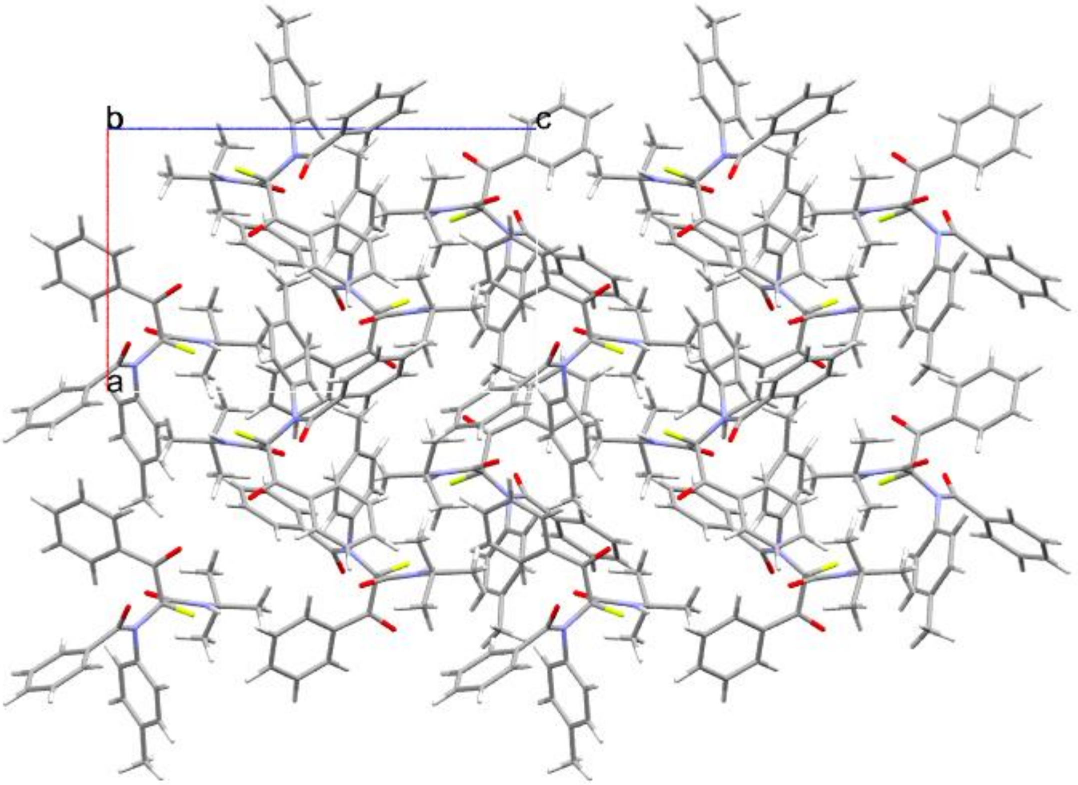

Packing diagram of the crystal structure of (*S*)-**12e**, viewed down the crystallographic *b*-axis.

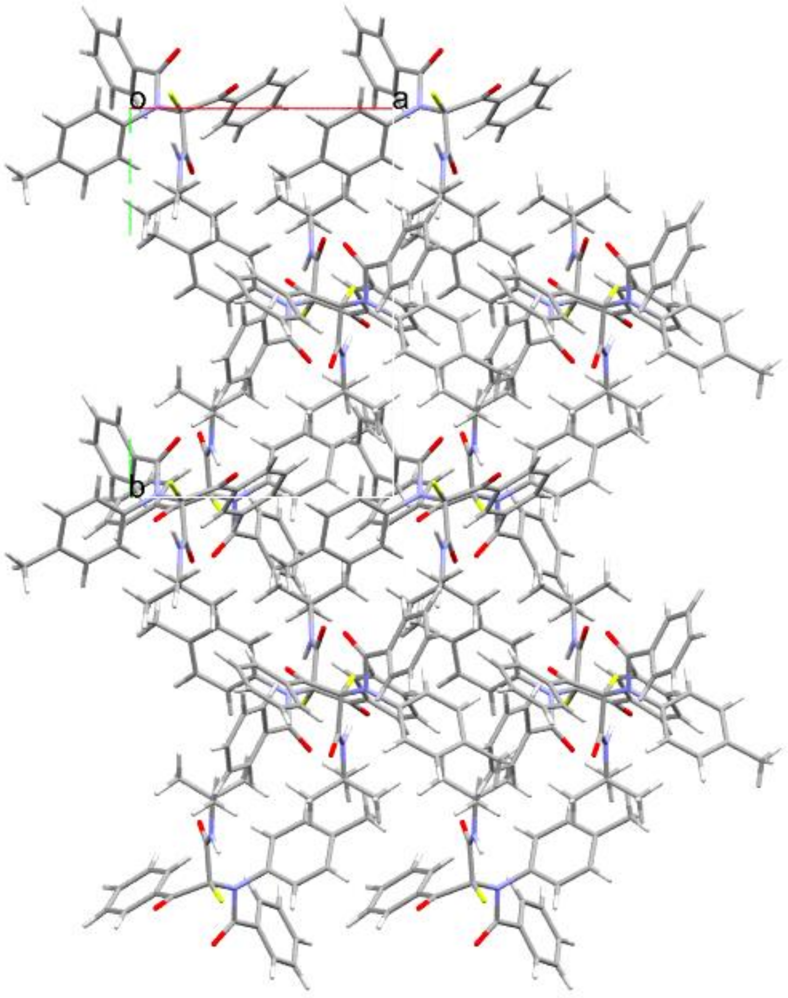

Packing diagram of the crystal structure of (*S*)-**12e**, viewed down the crystallographic *c*-axis.

Synthesis and characterization

General information

Unless otherwise specified, starting materials, reagents, and solvents were purchased from commercial sources and used as received. Q-Bn [5,6], Q-Ac [7], Q-Bz [7], DHQ-Bn [8], QD-Me [9], QD-Ac [7], QD-Bn [5,10,11] were synthesized following previously described protocols. Melting points were measured using INESA WRR apparatus. NMR spectra were recorded using 400 MHz Bruker Avance instruments. The ^{1}H and ^{13}C chemical shifts are reported relative to TMS using the residual $CDCl_3$ or [D_6]DMSO signal as internal reference. ^{19}F NMR spectra were referenced externally to CF_3COOH (−76.5 ppm) in a sealed capillary. HRMS were performed on a Bruker microTOF-Q III. High performance liquid chromatography (HPLC) was performed on an Agilent 1200 Series chromatograph using Daicel Chiralpak AD-H column (0.46 cm × 25 cm).

Synthesis of dihydroquinidine benzyl ether (DHQD-Bn)

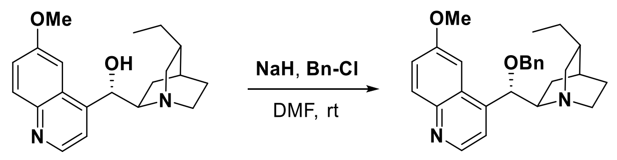

DHQD   DHQD-Bn

Adapted from the reported syntheses of similar cinchona alkaloid benzyl ethers [5,6,8,10,11], DHQD (2.0 g, 6.2 mmol) was dissolved in dry DMF (20 mL) followed by addition of NaH (0.68 g, 60% dispersion in mineral oil, 17 mmol). The resulting mixture was stirred at room temperature under inert atmosphere for 2 h. Then, benzyl chloride (0.75 mL, 0.83 g, 6.5 mmol) was added dropwise over the course of 10 minutes. The resulting mixture was stirred at room temperature under inert atmosphere for 17 h. Upon completion of this time, brine (50 mL) was added carefully and the resulting mixture was extracted with EtOAc (40 mL). The organic phase was washed with water (3 × 100 mL), dried over Na_2SO_4, and concentrated under reduced pressure. Column chromatography with ethyl acetate/methanol 99:1 provided DHQD-Bn as light yellow oil. $[\alpha]_D^{25}$ = + 117.6 (c 0.86, $CHCl_3$); ^{1}H NMR (400 MHz, $CDCl_3$) δ 8.75 (d, J = 4.5 Hz, 1H), 8.05 (d, J = 9.2 Hz, 1H), 7.48 (bd, J = 4.4 Hz, 1H), 7.41 – 7.27 (m, 7H), 5.26 (bs, 1H), 4.48 (d, J = 11.4 Hz, 1H), 4.38 (d, J = 11.4 Hz, 1H), 3.90 (s, 3H), 3.10 – 2.93 (m, 2H), 2.92 – 2.64 (m, 3H), 2.08 – 1.97 (m, 1H), 1.70 – 1.63 (m, 1H), 1.52 – 1.33 (m, 5H), 1.28 – 1.15

5. He, P.; Liu, X.; Shi, J.; Lin, L.; Feng, X. *Org. Lett.* **2011,** *13,* 936–939.

6. Maddox, S. M.; Dawson, G. A.; Rochester, N. C.; Ayonon, A. B.; Moore, C. E.; Rheingold, A. L.; Gustafson, J. L. *ACS Catal.* **2018,** *8,* 5443–5447.

7. Hutzler, J. M.; Walker, G. S.; Wienkers, L. C. *Chem. Res. Toxicol.* **2003,** *16,* 450–459.

8. Kohler, M. C.; Yost, J. M.; Garnsey, M. R.; Coltart, D. M. *Org. Lett.* **2010,** *12,* 3376–3379.

9. Papageorgiou, C. D.; de Dios, M. A. C.; Ley, S. V.; Gaunt, M. J. *Angew. Chem. Int. Ed.* **2004,** *43,* 4641–4644.

10. Medina, S.; Harper, M.; Balmond, E.; Miranda, S.; Crisenza, G. E. M.; Coe, D.; McGarrigle, E.; Galan, M. C. *Org. Lett.* **2016,** *18,* 4222–4225.

11. Li, H. M.; Wang, Y.; Tang, L.; Deng, L. *J. Am. Chem. Soc.* **2004,** *126,* 9906–9907.

(m, 1H), 0.84 (t, J = 7.1 Hz, 3H); ^{13}C NMR (100 MHz, $CDCl_3$) δ 157.9, 147.6, 144.9, 144.7, 138.0, 131.8, 128.4, 128.0, 127.8, 127.6, 121.9, 119.0 (bs), 101.3, 80.8 (bs), 71.4, 60.1, 55.7, 51.1, 50.3, 37.5, 27.4, 26.5, 25.3, 21.8 (bs), 12.0; HRMS (ESI, $[M+H]^+$) for $C_{27}H_{33}N_2O_2^+$ calcd. 417.2537, found 417.2556.

General procedure for the synthesis of Ugi adducts 8

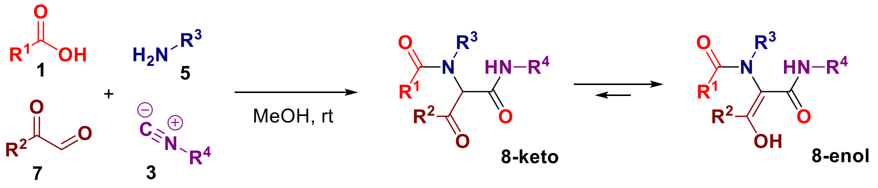

Analogous as described in [12,13], arylglyoxal monohydrate (**7**·H_2O, 1 mmol) was dissolved in methanol (5 mL), followed by addition of carboxylic acid (**1**, 1 mmol), amine (**5**, 1 mmol), and isocyanide (**3**, 1 mmol). The resulting mixture was stirred at room temperature for 24 hours. Upon completion of the reaction time, the mixture was diluted with ethyl acetate and concentrated with silica. Column chromatography with petroleum ether/ethyl acetate 19:1 → 9:1 as eluent followed by triturating with hexane delivered pure Ugi adducts **8a–h**, while Ugi adduct **8i** was not triturated after the chromatography.

***N*-Benzyl-*N*-(1-(*tert*-butylamino)-1,3-dioxo-3-phenylpropan-2-yl)benzamide (8a)**

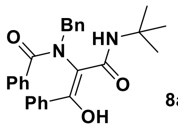

8a-enol

Yield: 62%; the analytical data was consistent with what is described in [12].

***N*-Benzyl-*N*-(1-(*tert*-butylamino)-1,3-dioxo-3-phenylpropan-2-yl)-4-nitrobenzamide (8b)**

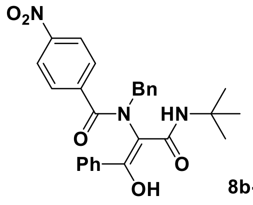

8b-enol

Yield: 79%; pale yellow solid; mp 156-158 °C; in $CDCl_3$ observed as an enol form; ^{1}H NMR (400 MHz, $CDCl_3$): δ 15.34 (s, 1H), 8.03 (d, J = 8.9 Hz, 2H), 7.53 – 7.43 (m, 7H), 7.43 – 7.37 (m, 3H), 7.21 (d, J =

12. Wei, H.; Wang, G.; Wang, Y.; Li, B.; Huang, J.; Kashtanov, S.; Van Hecke, K.; Pereshivko, O. P.; Peshkov, V. A. *Chem. Asian J.* **2017,** *12*, 825–829.

13. Hasan, M.; Zaman, M.; Peshkov, A. A.; Amire, N.; Les, A.; Nechaev, A. A.; Wang, Y.; Kashtanov, S.; Van der Eycken, E. V.; Pereshivko, O. P.; Peshkov, V. A. *Eur. J. Org. Chem.* **2020,** 3378–3389.

8.9 Hz, 2H), 5.87 (d, J = 13.9 Hz, 1H), 5.24 (bs, 1H), 3.82 (d, J = 13.9 Hz, 1H), 0.95 (s, 9H); ^{13}C NMR (100 MHz, $CDCl_3$) δ 170.61, 170.55, 169.3, 148.5, 142.0, 137.7, 133.5, 131.1, 129.7, 129.5, 128.90, 128.87, 127.8, 127.6, 123.0, 110.3, 55.9, 51.7, 28.1; HRMS (ESI, $[M+H]^+$) for $C_{27}H_{28}N_3O_5^+$ calcd. 474.2024, found 474.2033.

***N*-Benzyl-*N*-(1-(*tert*-butylamino)-3-(4-cyanophenyl)-1,3-dioxopropan-2-yl)benzamide (8c)**

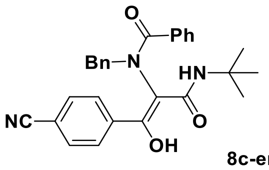

8c-enol

Yield: 72%; yellow solid; mp 163 – 165 °C; in $CDCl_3$ observed as an enol form; ^{1}H NMR (400 MHz, $CDCl_3$): δ 15.36 (s, 1H), 7.66 (d, J = 8.4 Hz, 2H), 7.53 (d, J = 8.4 Hz, 2H), 7.50 – 7.43 (m, 2H), 7.41 – 7.31 (m, 4H), 7.25 – 7.15 (m, 4H), 5.78 (d, J = 13.8 Hz, 1H), 5.32 (bs, 1H), 3.74 (d, J = 13.8 Hz, 1H), 0.93 (s, 9H); ^{13}C NMR (100 MHz, $CDCl_3$) δ 171.7, 170.3, 166.1, 138.1, 137.6, 135.1, 132.3, 130.8, 129.5, 129.4, 128.7, 128.2, 128.0, 127.2, 118.2, 114.0, 112.2, 55.5, 51.7, 27.9; HRMS (ESI, $[M+Na]^+$) for $C_{28}H_{27}N_3O_3Na^+$ calcd. 476.1945, found 476.1948.

***N*-(1-(*tert*-Butylamino)-1,3-dioxo-3-phenylpropan-2-yl)-*N*-phenylbenzamide (8d)**

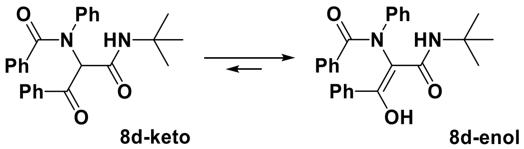

Yield: 67%; the analytical data was consistent with what is described in [13].

***N*-(1-(*tert*-Butylamino)-1,3-dioxo-3-phenylpropan-2-yl)-*N*-(*p*-tolyl)benzamide (8e)**

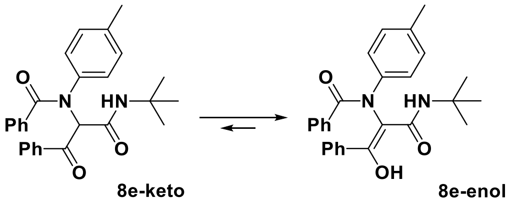

Yield: 56%; white solid; mp 154 – 156 °C; in $CDCl_3$ observed as a 14:11 mixture of enol and keto forms; ^{1}H NMR (400 MHz, $CDCl_3$) δ 14.98 (s, 0.56H, enol), 7.92 – 7.84 (m, 0.88H, keto), 7.58 – 7.50 (m, 0.44H, keto), 7.46 – 7.07 (m, 9.68H, enol + keto), 6.99 – 6.86 (m, 3.44H, enol + keto), 6.21 (s, 0.44H, keto), 5.81 (bs, 0.56H, enol), 2.34 (s, 1.68H, enol), 2.22 (s, 1.32H, keto), 1.29 (s, 5.04H, enol), 1.25 (s, 3.96H, keto); ^{13}C NMR (100 MHz, $CDCl_3$) δ 195.8, 171.2, 171.1, 170.3, 170.2, 165.0, 139.3 (bs), 139.1, 137.4, 136.7, 136.2, 136.0, 135.0, 133.7, 133.2, 130.6, 130.4, 130.1, 129.9, 129.6, 128.7, 128.6, 128.39,

128.35, 128.1, 127.77, 127.75, 127.5, 127.2 (bs), 123.6, 109.0, 69.5, 52.0, 51.7, 28.8, 28.5, 21.1, 21.0; HRMS (ESI, $[M+Na]^+$) for $C_{27}H_{28}N_2O_3Na^+$ calcd. 451.1992, found 451.2007.

_N_-(1-(_tert_-Butylamino)-1,3-dioxo-3-phenylpropan-2-yl)-_N_-(4-fluorophenyl)benzamide (8f)

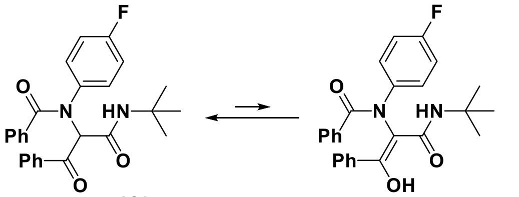

8f-keto 8f-enol

Yield: 48%; pale yellow solid, mp 128 – 130°C; in $CDCl_3$ observed as a 2:3 mixture of enol and keto forms; ^{1}H NMR (400 MHz, $CDCl_3$) δ 14.99 (s, 0.4H, enol), 7.92 – 7.84 (m, 1.2H, keto), 7.61 – 7.51 (m, 0.6H, keto), 7.49 – 7.19 (m, 6.2H, enol + keto), 7.18 – 7.01 (m, 4H, enol + keto), 6.99 – 6.93 (m, 0.8H, enol), 6.86 – 6.68 (m, 1.8H, keto), 6.40 – 6.31 (m, 0.6H, keto), 5.74 (bs, 0.4H, enol), 1.31 (s, 3.6H, enol), 1.24 (s, 5.4H, keto); ^{13}C NMR (100 MHz, $CDCl_3$) δ 195.9, 171.2, 171.1, 170.6, 170.2, 164.7, 161.5 (d, J = 248.4 Hz), 160.3 (d, J = 247.0 Hz), 138.1 (m), 137.5 (d, J = 3.2 Hz), 136.5, 135.8, 134.8, 133.6, 133.5, 130.74 (d, J = 7.0 Hz), 130.71 (d, J = 8.6 Hz), 130.1, 128.7, 128.6, 128.5, 128.2, 128.0, 127.9, 127.5, 127.3 (bs), 125.5, 125.4, 116.3 (d, J = 22.7 Hz), 115.79 (d, J = 22.7 Hz), 108.9, 68.7, 52.2, 52.0, 28.9, 28.5; HRMS (ESI, $[M+Na]^+$) for $C_{26}H_{25}FN_2O_3Na^+$ calcd. 455.1741, found 455.1762.

_N_-(1-(_tert_-Butylamino)-1,3-dioxo-3-phenylpropan-2-yl)-_N_-(4-chlorophenyl)benzamide (8g)

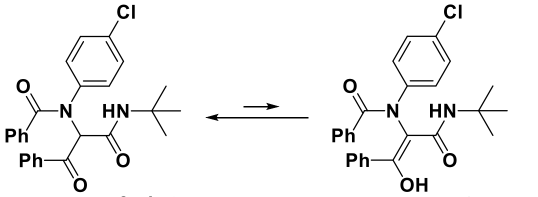

8g-keto 8g-enol

Yield: 39%; white solid; mp 131 – 133 °C; in $CDCl_3$ observed as a 3:7 mixture of enol and keto forms; ^{1}H NMR (400 MHz, $CDCl_3$) δ 15.01 (s, 0.3H, enol), 7.87 – 7.81 (m, 1.4H, keto), 7.60 – 7.53 (m, 0.7H, keto), 7.47 – 7.21 (m, 6.5H, enol + keto), 7.19 – 7.12 (m, 2H, enol + keto), 7.09 (d, J = 8.8 Hz, 1.4H, keto), 6.99 (d, J = 8.8 Hz, 1.4H, keto), 6.97 – 6.93 (m, 0.6H, enol), 6.79 (bs, 0.7H, keto), 6.29 (s, 0.7H, keto), 5.71 (bs, 0.3H, enol), 1.31 (s, 2.7H, enol), 1.27 (s, 6.3H, keto); ^{13}C NMR (100 MHz, $CDCl_3$) δ 195.9, 171.2, 171.0, 170.8, 170.1, 164.6, 140.6, 140.2, 136.6, 135.7, 134.5, 133.5, 133.4, 133.2, 131.4, 130.83, 130.81, 130.3, 129.9, 129.6, 129.1, 128.7, 128.5, 128.1, 128.0, 127.9, 127.5, 127.3, 124.9, 108.6, 68.6, 52.2, 52.0, 28.9, 28.6; HRMS (ESI, $[M+Na]^+$) for $C_{26}H_{25}ClN_2O_3Na^+$ calcd. 471.1446, found 471.1515.

***N*-(1-(*tert*-Butylamino)-3-(4-methoxyphenyl)-1,3-dioxopropan-2-yl)-4-chloro-*N*-(4-methoxyphenyl)benzamide (8h)**

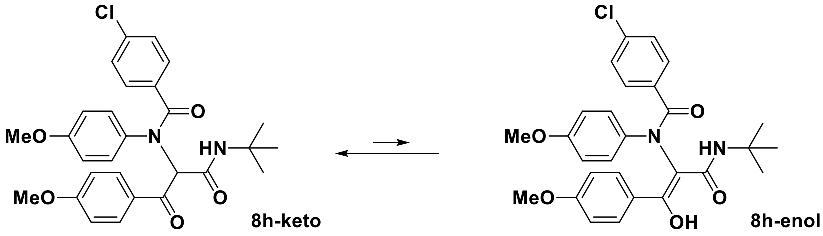

8h-keto 8h-enol

Yield: 68%; white solid; mp 87-89 °C; in $CDCl_3$ observed as a 17:83 mixture of enol and keto forms; ^{1}H NMR (400 MHz, $CDCl_3$) δ 15.01 (s, 0.17H, enol), 7.94 (d, *J* = 8.9 Hz, 1.66H, keto), 7.36 (d, *J* = 8.8 Hz, 0.34H, enol), 7.22 (d, *J* = 8.6 Hz, 1.66H, keto), 7.14 – 7.07 (m, 2H, enol + keto), 7.04 – 6.83 (m, 4.34H, enol + keto), 6.79 (d, *J* = 9.0 Hz, 0.34H, enol), 6.73 (bs, 0.83H, keto), 6.62 (d, *J* = 9.0 Hz, 1.66H, keto), 6.51 (s, 0.83H, keto), 5.71 (bs, 0.17H, enol), 3.85 (s, 2.49H, keto), 3.82 (bs, 0.51H, enol), 3.79 (s, 0.51H, enol), 3.70 (s, 2.49H, keto), 1.27 (s, 1.53H, enol), 1.20 (s, 7.47H, keto); ^{13}C NMR (100 MHz, $CDCl_3$) δ 194.0, 170.4, 170.3, 170.2, 170.1, 164.8, 164.1, 161.6, 158.8, 157.7, 136.4, 135.8, 133.8, 133.4, 130.8, 130.4, 130.1, 129.4, 129.0, 128.3 (bs), 128.1, 128.0, 124.8 (bs), 114.9, 114.1, 114.0, 113.9, 67.4, 55.7, 55.6, 55.4, 52.0, 51.8, 28.8, 28.5; HRMS (ESI, $[M+H]^+$) for $C_{28}H_{30}ClN_2O_5^+$ calcd. 509.1838, found 509.1821.

***N*-(1-(Butylamino)-1,3-dioxo-3-phenylpropan-2-yl)-*N*-phenylbenzamide (8i)**

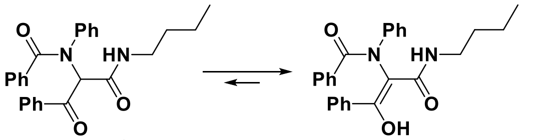

8i-keto 8i-enol

Yield: 70%; pale yellow oil; in $CDCl_3$ observed as a 2:1 mixture of enol and keto forms; ^{1}H NMR (400 MHz, $CDCl_3$) δ 14.90 (s, 0.67H, enol), 7.86 – 7.80 (m, 0.66H, keto), 7.59 – 7.53 (m, 0.33H, keto), 7.51 – 7.02 (m, 13H, enol + keto), 6.97 – 6.91 (m, 1.34H, enol), 6.14 (s, 0.33H, keto), 5.87 (bt, *J* = 5.5 Hz, 0.67H, enol), 3.45 – 3.34 (m, 0.67H, enol), 3.33 – 3.17 (m, 1.33H, enol + keto), 1.47 – 1.22 (m, 2.66H, enol + keto), 1.13 – 1.02 (m, 1.34H, enol), 0.87 (t, *J* = 7.3 Hz, 0.99H, keto), 0.81 (t, *J* = 7.3 Hz, 2.01H, enol); ^{13}C NMR (100 MHz, $CDCl_3$) δ 195.1, 171.4, 171.2, 170.3, 170.1, 166.1, 141.7, 141.5, 136.4, 135.8, 134.7, 133.3, 133.1, 130.6, 130.5, 130.0, 129.9, 129.4, 128.7, 128.5, 128.42, 128.36, 128.3, 127.9, 127.7, 127.4, 127.31, 127.26, 126.0, 123.7, 108.5, 68.8, 39.6, 39.0, 31.3, 31.1, 19.9, 19.5, 13.7, 13.6; HRMS (ESI, $[M+H]^+$) for $C_{26}H_{27}N_2O_3^+$ calcd. 415.2016, found 415.2021.

General procedure for cinchona alkaloid-promoted electrophilic fluorination of Ugi adduct 8

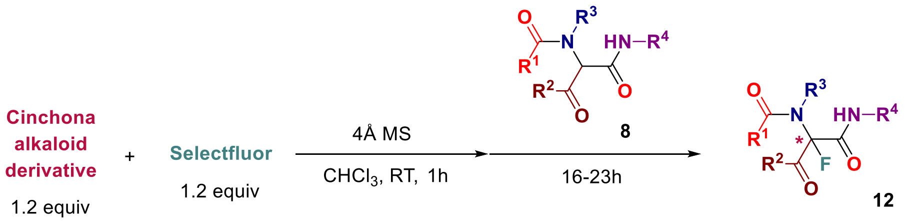

The cinchona alkaloid (0.18 mmol) was added to the reaction vial charged with 4 Å crushed molecular sieves (300 mg), followed by the addition of chloroform (2 mL), and Selectfluor (64 mg, 0.18 mmol). The resulting mixture was sealed and stirred at room temperature for 1 hour. Upon completion of this time the Ugi adduct **8** (0.15 mmol) dissolved in chloroform (2 mL) was added. The resulting mixture was stirred at room temperature for 21 hours (the reaction progress was monitored by TLC). After the starting compound **8** was consumed, the mixture was diluted with ethyl acetate and concentrated with a small amount of silica gel in order to perform a dry pack. Pure fluorinated products **12** were obtained by column chromatography using petroleum ether/ethyl acetate (the exact ratio was selected according to the TLC analysis) as eluent. Caution: Some of the products **12**, especially **12a–c** that were obtained from benzyl amine-derived Ugi adducts **8a–c**, were prone to a slow racemization over the prolonged storage at the room temperature. Therefore, all the samples of the isolated products **12** were stored in a fridge. In addition, we have tried to avoid any heating during the isolation of the most sensitive products **12a–c** (e.g., we did not use a water bath during the evaporation and utilized pre-cooled solvents for the column chromatography).

N-Benzyl-*N*-(1-(*tert*-butylamino)-2-fluoro-1,3-dioxo-3-phenylpropan-2-yl)benzamide (12a)

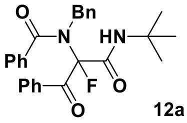

12a

White solid; mp 120 – 122 °C; $[\alpha]_D^{20}$ = + 149.8 (c 0.135, DCM, ee = 69 [faster enantiomer]); ^{1}H NMR (400 MHz, $CDCl_3$) δ 8.32 – 8.21 (m, 2H), 7.54 – 7.47 (m, 1H), 7.47 – 7.40 (m, 2H), 7.40 – 7.32 (m, 3H), 7.32 – 7.19 (m, 5H), 7.16 – 7.08 (m, 2H), 6.42 (d, *J* = 5.5 Hz, 1H), 4.99 (dd, *J* = 16.7, 1.9 Hz, 1H), 4.93 (d, *J* = 16.4 Hz, 1H), 1.22 (s, 9H); ^{13}C NMR (100 MHz, $CDCl_3$) δ 189.3 (bd, *J* = 26.8 Hz), 174.2 (d, *J* = 1.8 Hz), 161.8 (d, *J* = 25.9 Hz), 136.7 (d, *J* = 1.5 Hz), 134.6, 134.2, 132.8, 131.0, 129.5 (d, *J* = 2.1 Hz), 128.6, 128.5, 128.2, 127.74, 127.67 (d, *J* = 1.7 Hz), 127.2, 102.5 (d, *J* = 234.7 Hz), 52.4, 52.3 (d, *J* = 2.0 Hz), 28.2; ^{19}F NMR (377 MHz, $CDCl_3$) δ -120.6 (bs); HRMS (ESI, $[M+H]^+$) for $C_{27}H_{28}FN_2O_3^+$ calcd. 447.2079, found 447.2078; HPLC analysis: chiral AD-H column, *i*PrOH/Hexanes = 10:90, flow rate: 1.0 mL/min, retention times are ~ 37 min (faster enantiomer) and ~ 41 min (slower enantiomer).

ee = 73 (faster enantiomer), chiral promotor is DHQ-Bn

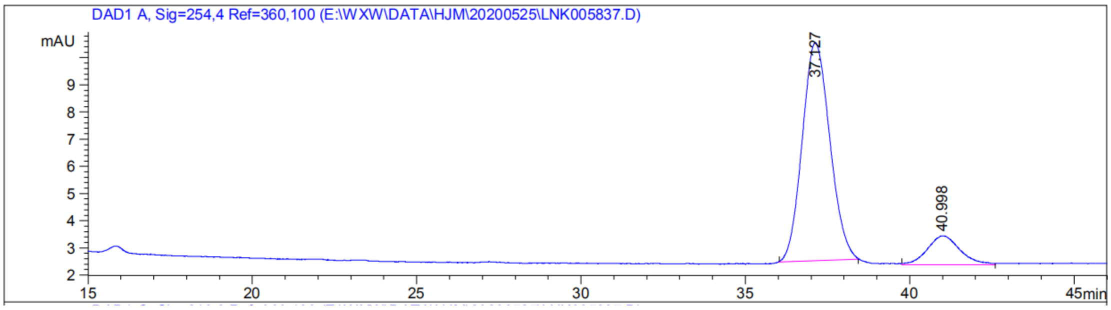

Peak #	RetTime [min]	Type	Width [min]	Area [mAU*s]	Height [mAU]	Area %
1	37.127	MM R	0.9752	469.57669	8.02542	86.5261
2	40.998	MM R	1.1563	73.12293	1.05396	13.4739

ee = 62 (slower enantiomer), chiral promotor is QD

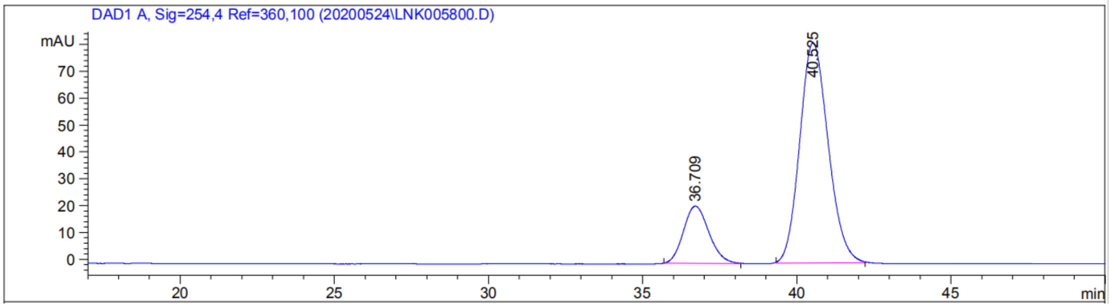

Peak #	RetTime [min]	Type	Width [min]	Area [mAU*s]	Height [mAU]	Area %
1	36.709	BB	0.8598	1230.31799	21.36578	18.9207
2	40.525	BB	0.9973	5272.17285	82.08831	81.0793

_N_-Benzyl-_N_-(1-(_tert_-butylamino)-2-fluoro-1,3-dioxo-3-phenylpropan-2-yl)-4-nitrobenzamide (12b)

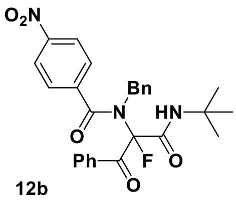

12b

White solid; mp 142 – 144 °C; ^{1}H NMR (400 MHz, $CDCl_3$) δ 8.26 – 8.19 (m, 2H), 8.12 (d, *J* = 8.8 Hz, 2H), 7.59 – 7.51 (m, 1H), 7.50 – 7.40 (m, 4H), 7.29 – 7.22 (m, 3H), 7.15 – 7.07 (m, 2H), 6.44 (d, *J* = 5.1 Hz, 1H), 4.93 (d, *J* = 16.9 Hz, 1H), 4.81 (dd, *J* = 16.8, 2.4 Hz, 1H), 1.22 (s, 9H); ^{13}C NMR (100

MHz, $CDCl_3$) δ 189.1 (d, *J* = 27.2 Hz), 172.2 (d, *J* = 2.0 Hz), 161.4 (d, *J* = 25.4 Hz), 148.9, 140.3, 136.2 (d, *J* = 1.5 Hz), 134.5, 133.2, 129.5 (d, *J* = 2.7 Hz), 128.9, 128.3, 128.1, 127.4 (d, *J* = 1.5 Hz), 102.6 (d, *J* = 235.3 Hz), 52.7, 52.4 (d, *J* = 1.6 Hz), 28.2; ^{19}F NMR (377 MHz, $CDCl_3$) δ -121.0 (bs); HRMS (ESI, $[M+H]^+$) for $C_{27}H_{27}FN_2O_5^+$ calcd. 492.1929, found 492.1904; HPLC analysis: chiral AD-H column, *i*PrOH/Hexanes = 30:70, flow rate: 1.0 mL/min, retention times are ~ 26 min (faster enantiomer) and ~ 37 min (slower enantiomer).

ee = 51 (slower enantiomer), chiral promotor is DHQ-Bn

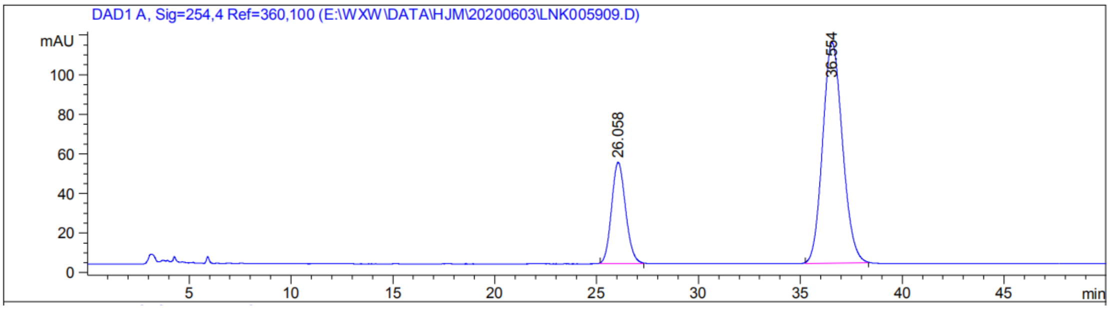

Peak #	RetTime [min]	Type	Width [min]	Area [mAU*s]	Height [mAU]	Area %
1	26.058	BB	0.7400	2443.36597	51.08418	24.4455
2	36.554	BB	1.0505	7551.77588	111.44859	75.5545

ee = 24 (faster enantiomer), chiral promotor is QD-Bn

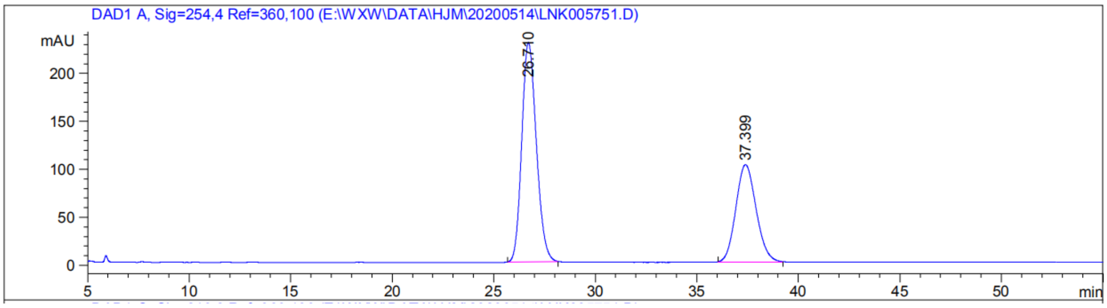

Peak #	RetTime [min]	Type	Width [min]	Area [mAU*s]	Height [mAU]	Area %
1	26.710	BB	0.7749	1.14443e4	229.03825	61.8749
2	37.399	BB	1.0761	7051.55762	101.55120	38.1251

***N*-Benzyl-*N*-(1-(*tert*-butylamino)-3-(4-cyanophenyl)-2-fluoro-1,3-dioxopropan-2-yl)benzamide (12c)**

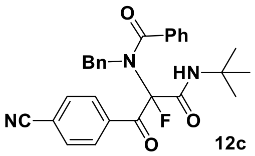

12c

Pale yellow solid; mp 130 – 132 °C; ^{1}H NMR (400 MHz, $CDCl_3$) δ 8.36 (bd, *J* = 7.8 Hz, 2H), 7.72 (d, *J* = 8.7 Hz, 2H), 7.45 – 7.38 (m, 1H), 7.36 – 7.28 (m, 4H), 7.28 – 7.21 (m, 3H), 7.15 – 7.07 (m, 2H), 6.38 (d, *J* = 5.8 Hz, 1H), 5.04 (dd, *J* = 16.6, 2.0 Hz, 1H), 4.91 (d, *J* = 16.6 Hz, 1H), 1.23 (s, 9H); ^{13}C NMR (100 MHz, $CDCl_3$) δ 188.1 (bd, *J* = 28.0 Hz), 174.4 (d, *J* = 1.7 Hz), 161.4 (d, *J* = 25.7 Hz), 138.4, 136.3 (d, *J* = 1.5 Hz), 133.6, 131.9, 131.4, 129.8 (d, *J* = 1.9 Hz), 128.8, 128.7, 128.0, 127.7 (d, *J* = 1.5 Hz), 127.1, 118.2, 115.9, 102.1 (d, *J* = 235.6 Hz), 52.7, 52.4 (d, *J* = 1.2 Hz), 28.2; ^{19}F NMR (377 MHz, $CDCl_3$) δ -120.9 (bs); HRMS (ESI, $[M+H]^+$) for $C_{28}H_{27}FN_3O_3^+$ calcd. 472.2031, found 472.2031; HPLC analysis: chiral AD-H column, *i*PrOH/Hexanes = 20:80, flow rate: 1.0 mL/min, retention times are ~ 30 min (faster enantiomer) and ~ 39 min (slower enantiomer).

ee = 43 (slower enantiomer), chiral promotor is DHQ

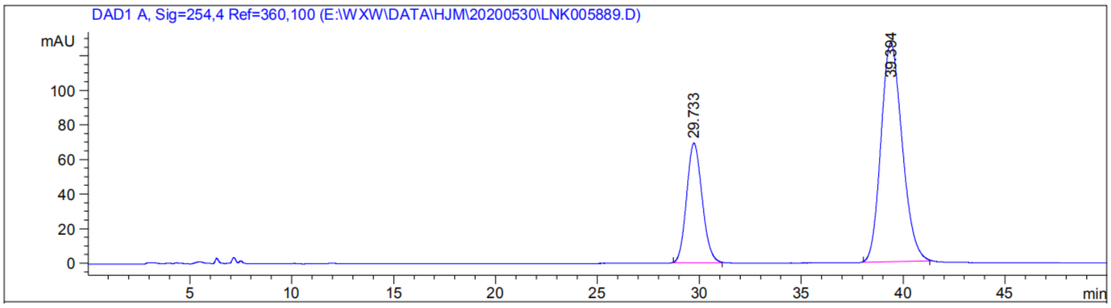

Peak #	RetTime [min]	Type	Width [min]	Area [mAU*s]	Height [mAU]	Area %
1	29.733	BB	0.8153	3660.97266	69.18863	28.5165
2	39.394	BB	1.1234	9177.09082	126.64333	71.4835

ee = 30 (faster enantiomer), chiral promotor is QD

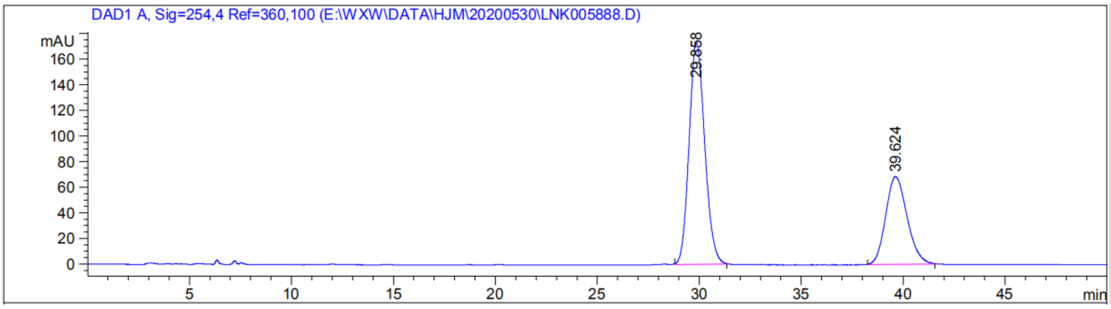

Peak #	RetTime [min]	Type	Width [min]	Area [mAU*s]	Height [mAU]	Area %
1	29.858	BB	0.8262	9222.59473	172.94363	64.8830
2	39.624	BB	1.1275	4991.60156	68.39449	35.1170

***N*-(1-(*tert*-Butylamino)-2-fluoro-1,3-dioxo-3-phenylpropan-2-yl)-*N*-phenylbenzamide (12d)**

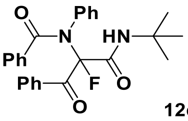

12d

White solid; mp 133 – 135 °C; $[\alpha]_D^{20}$ = + 180.3 (c 0.217, DCM, ee = 71 [slower enantiomer]); ^{1}H NMR (400 MHz, [D$_6$]DMSO) δ 8.25 (bs, 2H), 7.74 (bs, 1H), 7.65 – 7.52 (m, 3H), 7.49 – 7.38 (m, 2H), 7.34 – 7.16 (m, 8H), 0.97 (s, 9H); ^{13}C NMR (100 MHz, $CDCl_3$) δ 189.5 (bd, *J* = 28.2 Hz), 172.5, 161.0 (d, *J* = 25.8 Hz), 138.8 (d, *J* = 2.2 Hz), 135.2, 133.8, 132.7, 130.79 (bs), 130.75, 129.7 (d, *J* = 3.1 Hz), 129.2, 128.9, 128.6, 128.1, 127.9, 102.3 (d, *J* = 236.1 Hz), 52.0, 28.0; ^{19}F NMR (377 MHz, $CDCl_3$) δ -123.9 (bs); HRMS (ESI, $[M+H]^+$) for $C_{26}H_{26}FN_2O_3^+$ calcd. 433.1922, found 433.1922; HPLC analysis: chiral AD-H column, *i*PrOH/Hexanes = 30:70, flow rate: 1.0 mL/min, retention times are ~ 9 min (faster enantiomer) and ~ 12 min (slower enantiomer).

ee = 59 (faster enantiomer), chiral promotor is QD-Bn

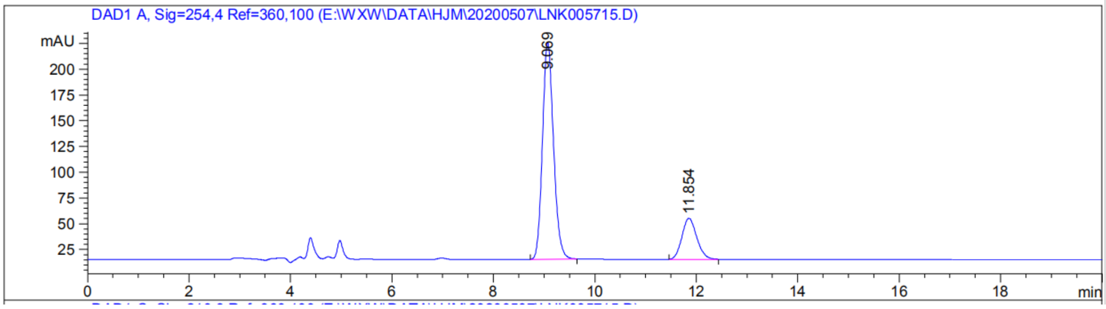

Peak #	RetTime [min]	Type	Width [min]	Area [mAU*s]	Height [mAU]	Area %
1	9.069	BB	0.2325	3182.16138	210.38148	79.5376
2	11.854	BB	0.3171	818.66614	39.94847	20.4624

ee = 71 (slower enantiomer), chiral promotor is DHQ-Bn

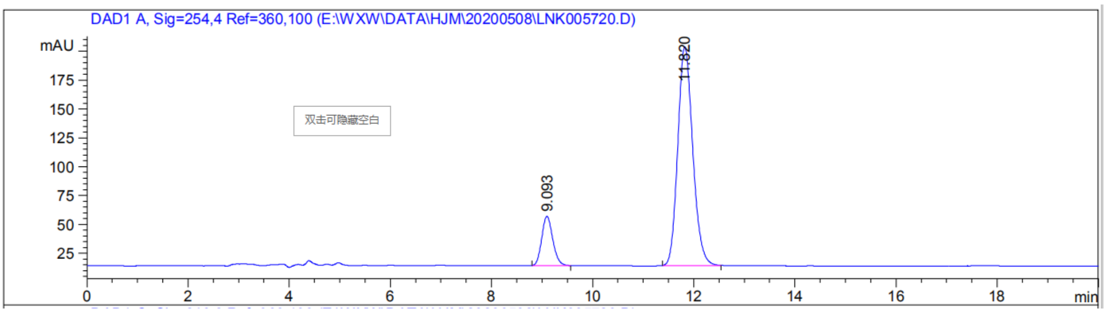

Peak #	RetTime [min]	Type	Width [min]	Area [mAU*s]	Height [mAU]	Area %
1	9.093	BB	0.2367	656.60040	42.85953	14.3962
2	11.820	BB	0.3186	3904.31860	189.33868	85.6038

_N_-(1-(_tert_-Butylamino)-2-fluoro-1,3-dioxo-3-phenylpropan-2-yl)-_N_-(_p_-tolyl)benzamide (12e)

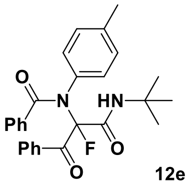

12e

White solid; mp 128 – 130 °C; $[\alpha]_D^{20}$ = + 192.5 (c 0.581, DCM, ee = 74 [slower enantiomer]); ^{1}H NMR (400 MHz, [D$_6$]DMSO) δ 8.24 (bs, 2H), 7.68 (bd, *J* = 2.8 Hz, 1H), 7.64 – 7.58 (m, 1H), 7.58 – 7.51 (m, 2H), 7.36 – 7.17 (m, 7H), 7.11 (d, *J* = 8.3 Hz, 2H), 2.22 (s, 3H), 0.98 (s, 9H); ^{13}C NMR (100 MHz, CDCl$_3$) δ 189.4 (bd, *J* = 25.6 Hz), 172.6, 161.0 (d, *J* = 25.9 Hz), 138.9, 136.0 (d, *J* = 2.2 Hz), 135.2, 133.8, 132.6, 130.7, 130.5 (bs), 129.7, 129.6 (d, *J* = 2.9 Hz), 128.5, 128.1, 127.8, 102.3 (d, *J* = 236.0 Hz), 51.9, 28.0, 21.1; ^{19}F NMR (377 MHz, CDCl$_3$) δ -123.8 (bs); HRMS (ESI, [M+H]$^+$) for $C_{27}H_{28}FN_2O_3^+$ calcd. 447.2079, found 447.2081; HPLC analysis: chiral AD-H column, *i*PrOH/Hexanes = 10:90, flow rate: 1.0 mL/min, retention times are ~ 36 min (faster enantiomer) and ~ 41 min (slower enantiomer).

ee = 61 (faster enantiomer), chiral promotor is QD-Bn

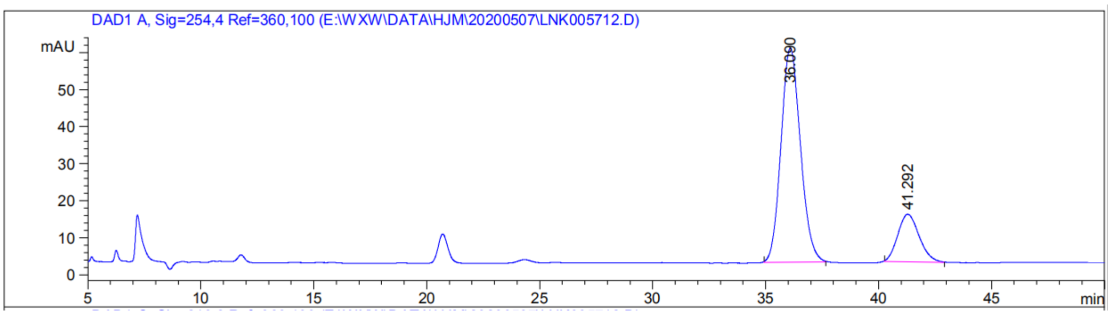

Peak #	RetTime [min]	Type	Width [min]	Area [mAU*s]	Height [mAU]	Area %
1	36.090	BB	0.9327	3457.74121	57.73540	80.4733
2	41.292	BB	0.9369	839.01526	12.79787	19.5267

ee = 74 (slower enantiomer), chiral promotor is DHQ-Bn

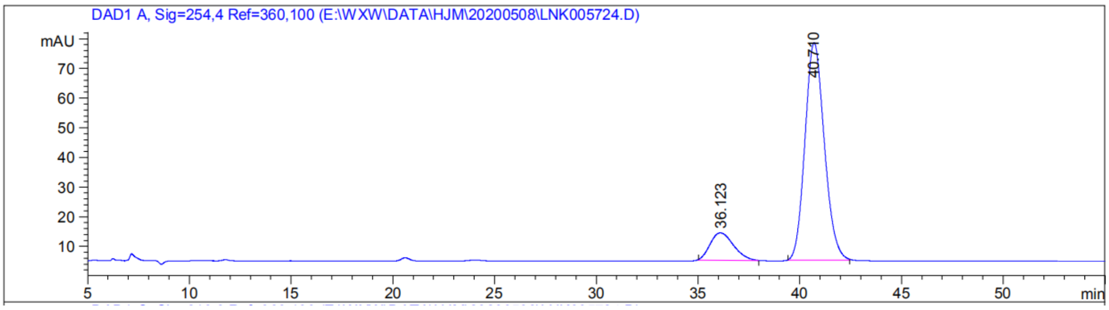

Peak #	RetTime [min]	Type	Width [min]	Area [mAU*s]	Height [mAU]	Area %
1	36.123	BB	0.9524	736.17999	9.19173	13.1752
2	40.710	BB	1.0203	4851.42139	73.28774	86.8248

_N_-(1-(_tert_-Butylamino)-2-fluoro-1,3-dioxo-3-phenylpropan-2-yl)-_N_-(4-fluorophenyl)benzamide (12f)

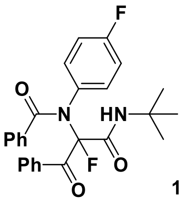

12f

White solid; mp 125 – 127 °C; $[\alpha]_D^{20}$ = + 177.0 (c 0.547, DCM, ee = 66 [slower enantiomer]); ^{1}H NMR (400 MHz, [D$_6$]DMSO) δ 8.22 (bs, 2H), 7.85 (bs, 1H), 7.66 – 7.59 (m, 1H), 7.59 – 7.44 (m, 4H), 7.33 – 7.27 (m, 1H), 7.27 – 7.20 (m, 4H), 7.19 – 7.12 (m, 2H), 1.00 (s, 9H); ^{13}C NMR (100 MHz, $CDCl_3$) δ 189.1 (bd, *J* = 26.7 Hz), 172.5, 162.4 (d, *J* = 250.3 Hz), 160.9 (d, *J* = 25.7 Hz), 135.1, 134.7 (dd, *J* = 3.1, 2.5 Hz), 133.6, 132.8, 132.7 (bd, *J* = 8.8 Hz), 130.9, 129.6 (d, *J* = 2.9 Hz), 128.5, 128.2, 128.1, 116.0 (d, *J* = 22.8 Hz), 102.3 (d, *J* = 236.3 Hz), 52.1, 28.1; ^{19}F NMR (377 MHz, $CDCl_3$) δ (-111.6) – (-111.8) (m), -124.0 (bs); HRMS (ESI, $[M+H]^+$) for $C_{26}H_{25}F_2N_2O_3^+$ calcd. 451.1828, found 451.1854; HPLC analysis: chiral AD-H column, *i*PrOH/Hexanes = 20:80, flow rate: 1.0 mL/min, retention times are ~ 14 min (faster enantiomer) and ~ 27 min (slower enantiomer).

ee = 58 (faster enantiomer), chiral promotor is QD-Bn

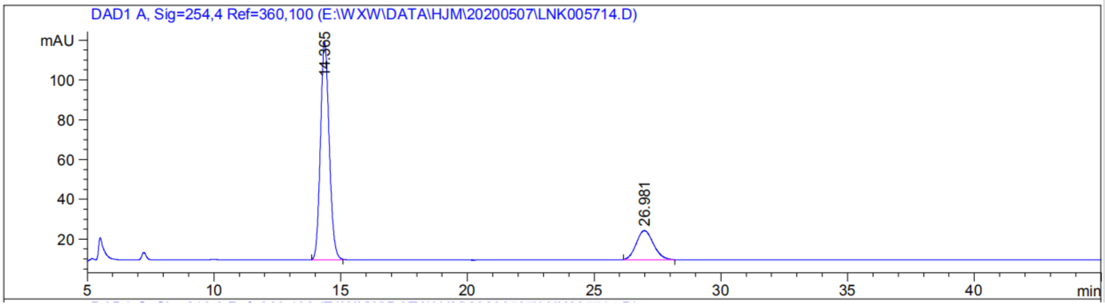

Peak #	RetTime [min]	Type	Width [min]	Area [mAU*s]	Height [mAU]	Area %
1	14.365	BB	0.3736	2639.30957	109.35254	79.2013
2	26.981	BB	0.7227	693.09692	14.68353	20.7987

ee = 72 (slower enantiomer), chiral promotor is DHQ-Bn

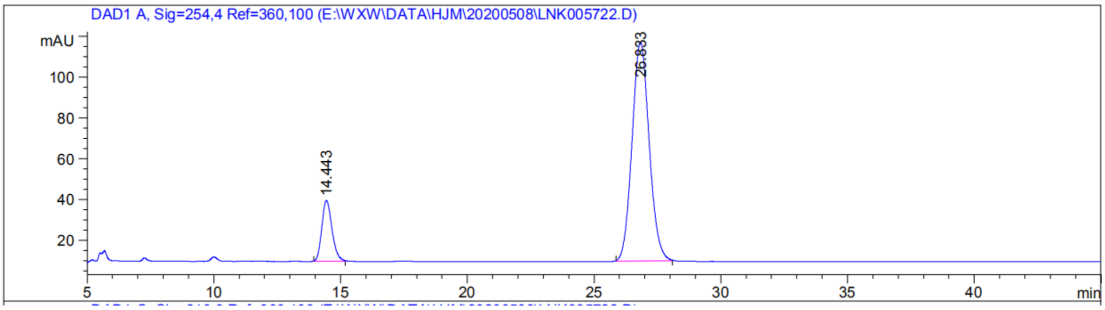

Peak #	RetTime [min]	Type	Width [min]	Area [mAU*s]	Height [mAU]	Area %
1	14.443	BB	0.4265	829.92725	29.83978	14.1275
2	26.833	BB	0.7295	5044.61621	107.10073	85.8725

N-(1-(tert-Butylamino)-2-fluoro-1,3-dioxo-3-phenylpropan-2-yl)-N-(4-chlorophenyl)benzamide (12g)

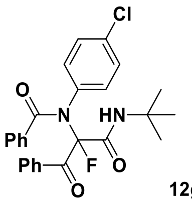

12g

White solid; mp 153 – 155 °C; $[\alpha]_D^{20}$ = + 186.9 (c 0.546, DCM, ee = 70 [slower enantiomer]); ^{1}H NMR (400 MHz, [D$_6$]DMSO) δ 8.21 (bs, 2H), 7.88 (bs, 1H), 7.66 – 7.59 (m, 1H), 7.59 – 7.52 (m, 2H), 7.52 – 7.42 (m, 2H), 7.39 (d, *J* = 8.9 Hz, 2H), 7.34 – 7.28 (m, 1H), 7.28 – 7.20 (m, 4H), 1.01 (s, 9H); ^{13}C NMR (100 MHz, CDCl$_3$) δ 189.1 (bd, *J* = 27.2 Hz), 172.3, 160.9 (d, *J* = 25.7 Hz), 137.3 (d, *J* = 2.2 Hz), 135.0 (bs), 134.9, 133.5, 132.9, 132.1 (bs), 131.1, 129.6 (d, *J* = 3.0 Hz), 129.3, 128.5, 128.2, 128.1, 102.2 (d, *J* = 236.5 Hz), 52.2, 28.1; ^{19}F NMR (377 MHz, CDCl$_3$) δ -124.1 (bs); HRMS (EI, [M+Na]$^+$) for $C_{26}H_{24}ClFN_2O_3Na^+$ calcd. 489.1351, found 489.1378; HPLC analysis: chiral AD-H column, *i*PrOH/Hexanes = 30:70, flow rate: 1.0 mL/min, retention times are ~ 12 min (faster enantiomer) and ~ 18 min (slower enantiomer).

ee = 60 (faster enantiomer), chiral promotor is QD-Bn

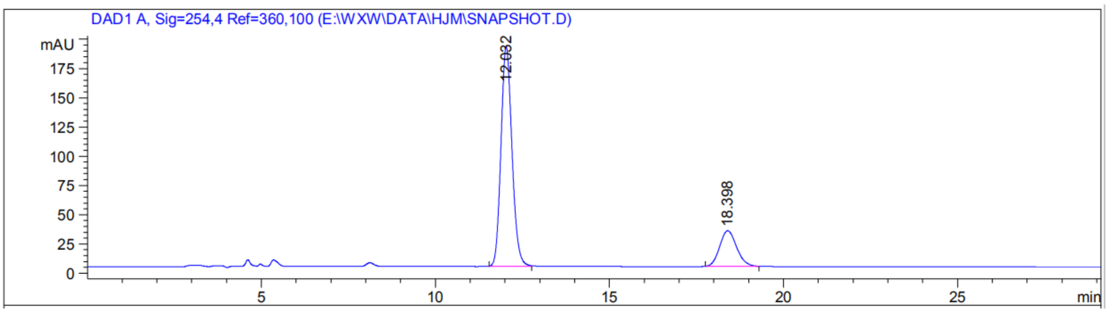

Peak #	RetTime [min]	Type	Width [min]	Area [mAU*s]	Height [mAU]	Area %
1	12.032	BB	0.3390	4137.33252	187.82994	79.8687
2	18.398	BB	0.5259	1042.83301	30.57278	20.1313

ee = 70 (slower enantiomer), chiral promotor is DHQ-Bn

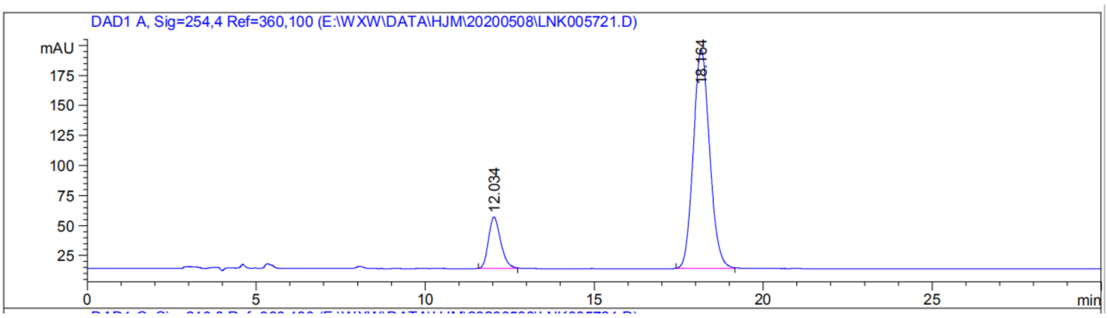

Peak #	RetTime [min]	Type	Width [min]	Area [mAU*s]	Height [mAU]	Area %
1	12.034	BB	0.3872	1075.07947	42.77799	14.9377
2	18.164	BB	0.5204	6122.02441	181.99448	85.0623

***N*-(1-(*tert*-Butylamino)-2-fluoro-3-(4-methoxyphenyl)-1,3-dioxopropan-2-yl)-4-chloro-*N*-(4-methoxyphenyl)benzamide (12h)**

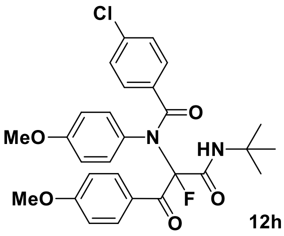

12h

White solid; mp 103-105 °C; $[\alpha]_D^{20}$ = + 189.0 (c 0.258, DCM, ee = 71 [faster enantiomer]); ^{1}H NMR (400 MHz, $CDCl_3$) δ 8.37 (bd, *J* = 8.7 Hz, 2H), 7.33 – 7.22 (m, 4H), 7.12 (d, *J* = 8.7 Hz, 2H), 6.93 (d, *J* = 9.1 Hz, 2H), 6.76 (d, *J* = 9.1 Hz, 2H), 6.37 (bd, *J* = 5.3 Hz, 1H), 3.84 (s, 3H), 3.75 (s, 3H), 1.07 (s, 9H); ^{13}C NMR (100 MHz, $CDCl_3$) δ 187.5 (bd, *J* = 23.9 Hz), 171.7, 163.4, 161.2 (d, *J* = 25.8 Hz), 159.7, 136.9, 132.5, 132.22 (d, *J* = 3.2 Hz), 132.0 (bs), 131.2 (d, *J* = 1.9 Hz), 130.2, 128.3, 127.6, 102.60 (d, *J* = 236.0 Hz), 55.53, 55.50, 52.0, 28.1; ^{19}F NMR (377 MHz, $CDCl_3$) δ -128.6 (bs); HRMS (ESI, $[M+H]^+$) for $C_{28}H_{29}ClFN_2O_5^+$ calcd. 527.1744, found 527.1749; HPLC analysis: chiral AD-H column, *i*PrOH/Hexanes = 20:80, flow rate: 1.0 mL/min, retention times are ~ 18 min (faster enantiomer) and ~ 26 min (slower enantiomer).

ee = 55 (slower enantiomer), chiral promotor is QD-Bn

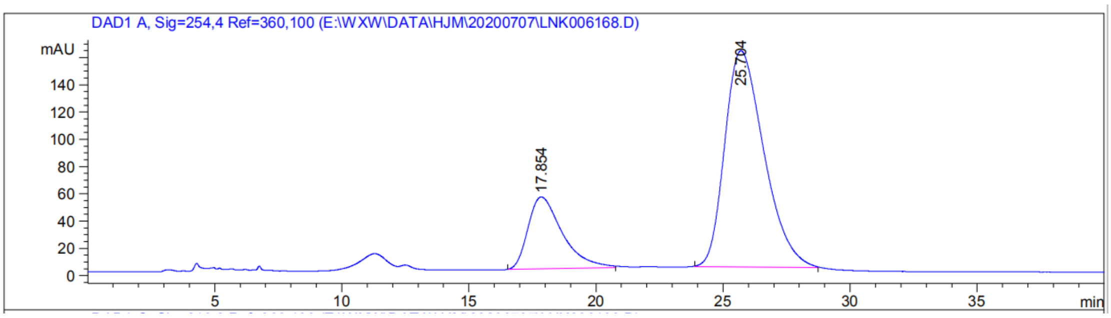

Peak #	RetTime [min]	Type	Width [min]	Area [mAU*s]	Height [mAU]	Area %
1	17.854	MM R	1.5647	4959.39258	52.82526	22.5677
2	25.704	BB	1.6417	1.70162e4	158.60370	77.4323

ee = 73 (faster enantiomer), chiral promotor is DHQ-Bn

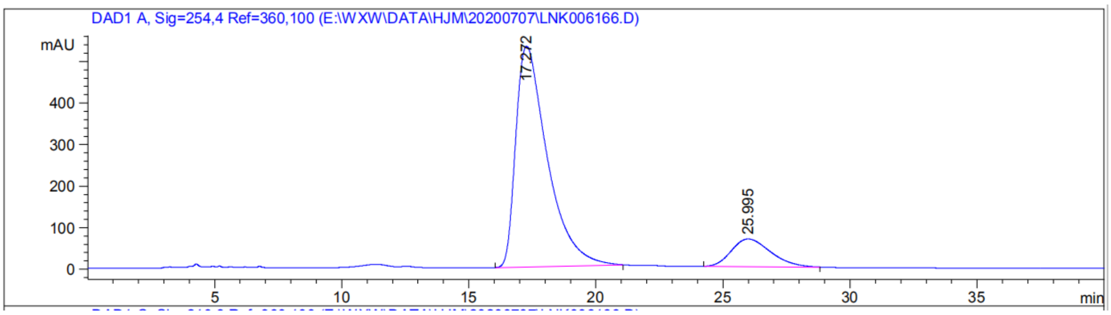

Peak #	RetTime [min]	Type	Width [min]	Area [mAU*s]	Height [mAU]	Area %
1	17.272	BB	1.2695	4.59521e4	531.04010	86.4561
2	25.995	BB	1.5765	7198.70166	66.73314	13.5439

_N_-(1-(Butylamino)-2-fluoro-1,3-dioxo-3-phenylpropan-2-yl)-_N_-phenylbenzamide (12i)

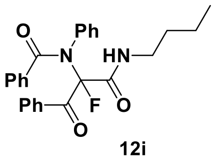

12i

White solid; mp 117 – 119 °C; $[\alpha]_D^{20}$ = + 162.9 (c 0.269, DCM, ee = 55 [faster enantiomer]); ^{1}H NMR (400 MHz, $CDCl_3$) δ 8.43 – 8.34 (m, 2H), 7.56 – 7.49 (m, 1H), 7.49 – 7.42 (m, 2H), 7.41 – 7.35 (m, 2H), 7.31 – 7.19 (m, 6H), 7.16 – 7.09 (m, 2H), 6.64 – 6.54 (m, 1H), 3.16 – 3.05 (m, 1H), 2.99 – 2.87 (m, 1H), 1.19 – 1.08 (m, 2H), 1.06 – 0.94 (m, 2H), 0.75 (t, J = 7.2 Hz, 3H); ^{13}C NMR (100 MHz, $CDCl_3$) δ 172.7, 162.2 (d, J = 27.1 Hz), 138.8 (d, J = 2.0 Hz), 134.9, 133.7, 132.9, 130.9, 130.7 (bs), 129.8 (d,

J = 2.9 Hz), 129.0, 128.7, 128.2, 128.0, 102.79 (d, *J* = 235.1 Hz), 39.6, 30.9, 19.7, 13.7; ^{19}F NMR (377 MHz, $CDCl_3$) δ -126.0 (bs); HRMS (ESI, $[M+Na]^+$) for $C_{26}H_{25}FN_2O_3Na^+$ calcd. 455.1741, found 455.1739; HPLC analysis: chiral AD-H column, *i*PrOH/Hexanes = 10:90, flow rate: 1.0 mL/min, retention times are ~ 30 min (faster enantiomer) and ~ 36 min (slower enantiomer).

ee = 29 (slower enantiomer), chiral promotor is QD-Bn

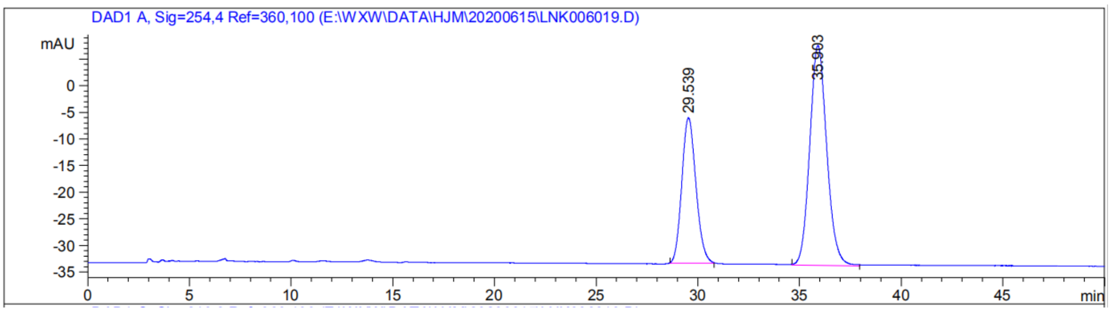

Peak #	RetTime [min]	Type	Width [min]	Area [mAU*s]	Height [mAU]	Area %
1	29.539	BB	0.7340	1320.68848	27.31943	35.4344
2	35.903	MM R	0.9704	2406.45044	41.32970	64.5656

ee = 55 (faster enantiomer), chiral promotor is QD

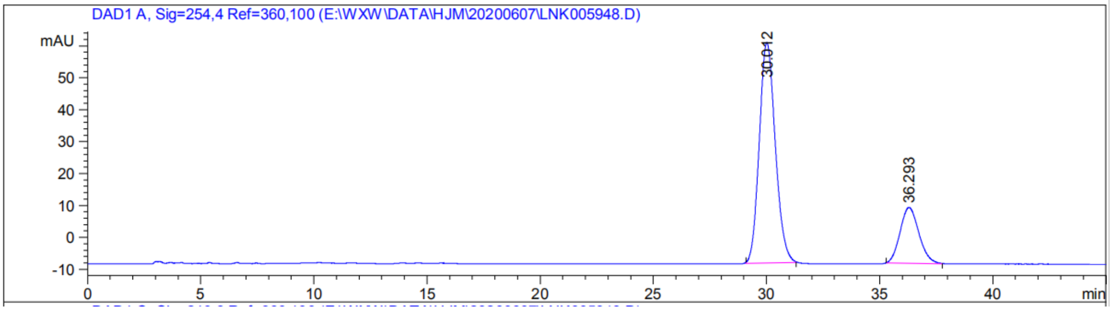

Peak #	RetTime [min]	Type	Width [min]	Area [mAU*s]	Height [mAU]	Area %
1	30.012	BB	0.7542	3402.79053	69.14426	77.1316
2	36.293	BB	0.8522	1008.87598	17.45934	22.8684

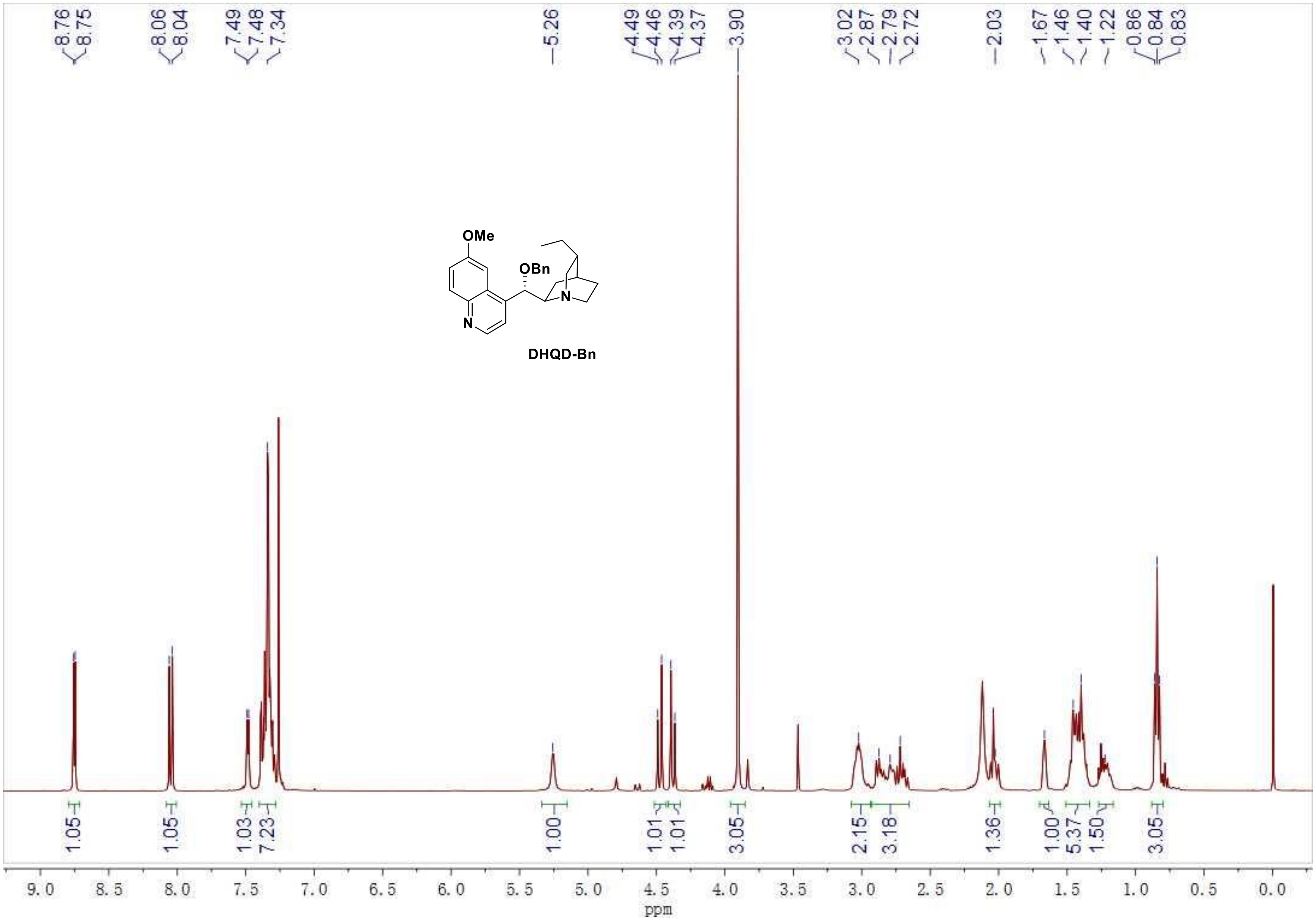

8.76
8.75
8.06
8.04
7.49
7.48
7.34
5.26
4.49
4.46
4.39
4.37
3.90
3.02
2.87
2.79
2.72
2.03
1.67
1.46
1.40
1.22
0.86
0.84
0.83
OMe
OBn
N
N
DHQD-Bn
1.05
1.05
1.03
7.23
1.00
1.01
1.01
3.05
2.15
3.18
1.36
1.00
5.37
1.50
3.05
9.0
8.5
8.0
7.5
7.0
6.5
6.0
5.5
5.0
4.5
4.0
3.5
3.0
2.5
2.0
1.5
1.0
0.5
0.0
ppm

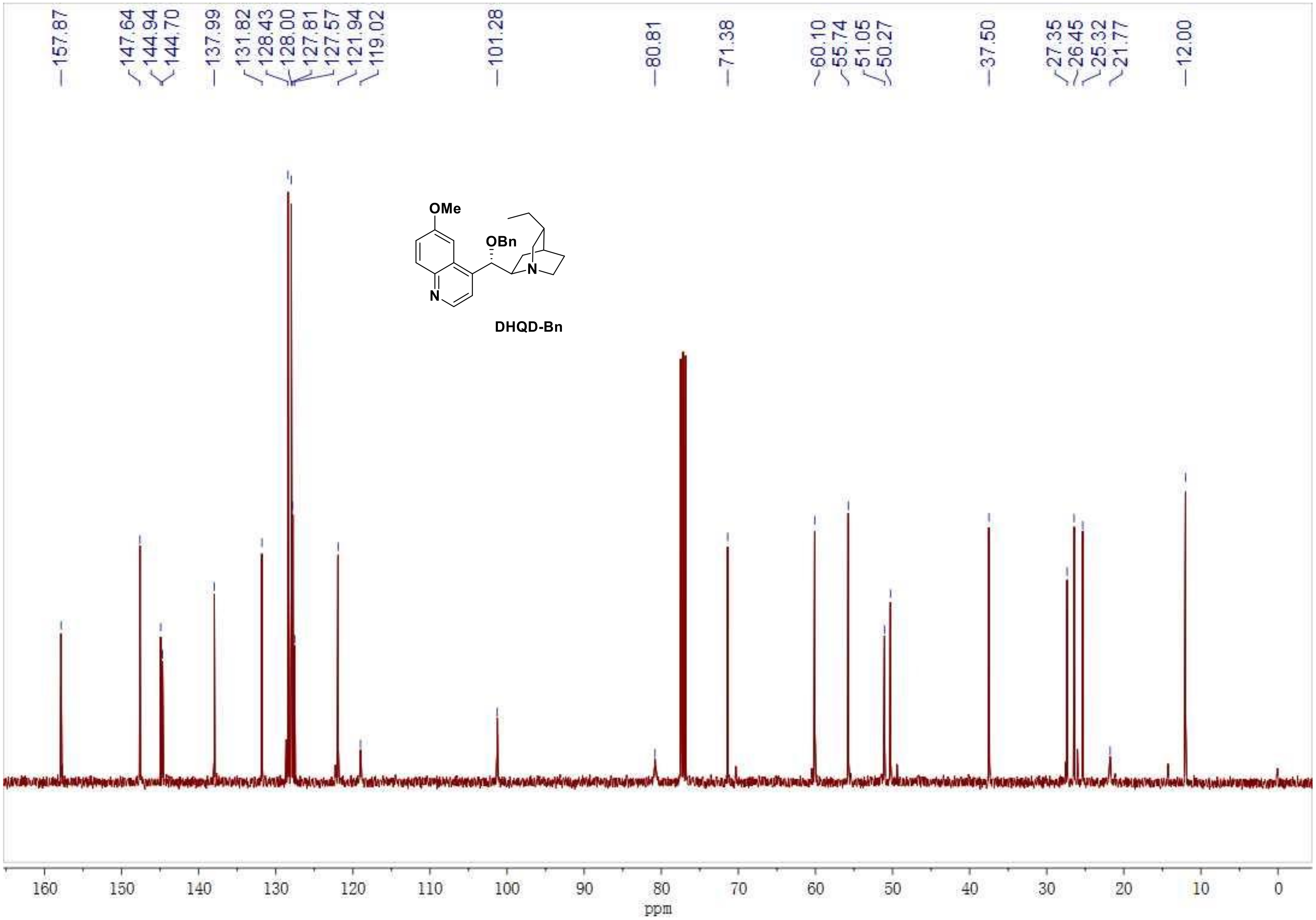
157.87
147.64
144.94
144.70
137.99
131.82
128.43
128.00
127.81
127.57
121.94
119.02
101.28
80.81
71.38
60.10
55.74
51.05
50.27
37.50
27.35
26.45
25.32
21.77
12.00
OMe
OBn
N
N
DHQD-Bn
160
150
140
130
120
110
100
90
80
70
60
50
40
30
20
10
0
ppm

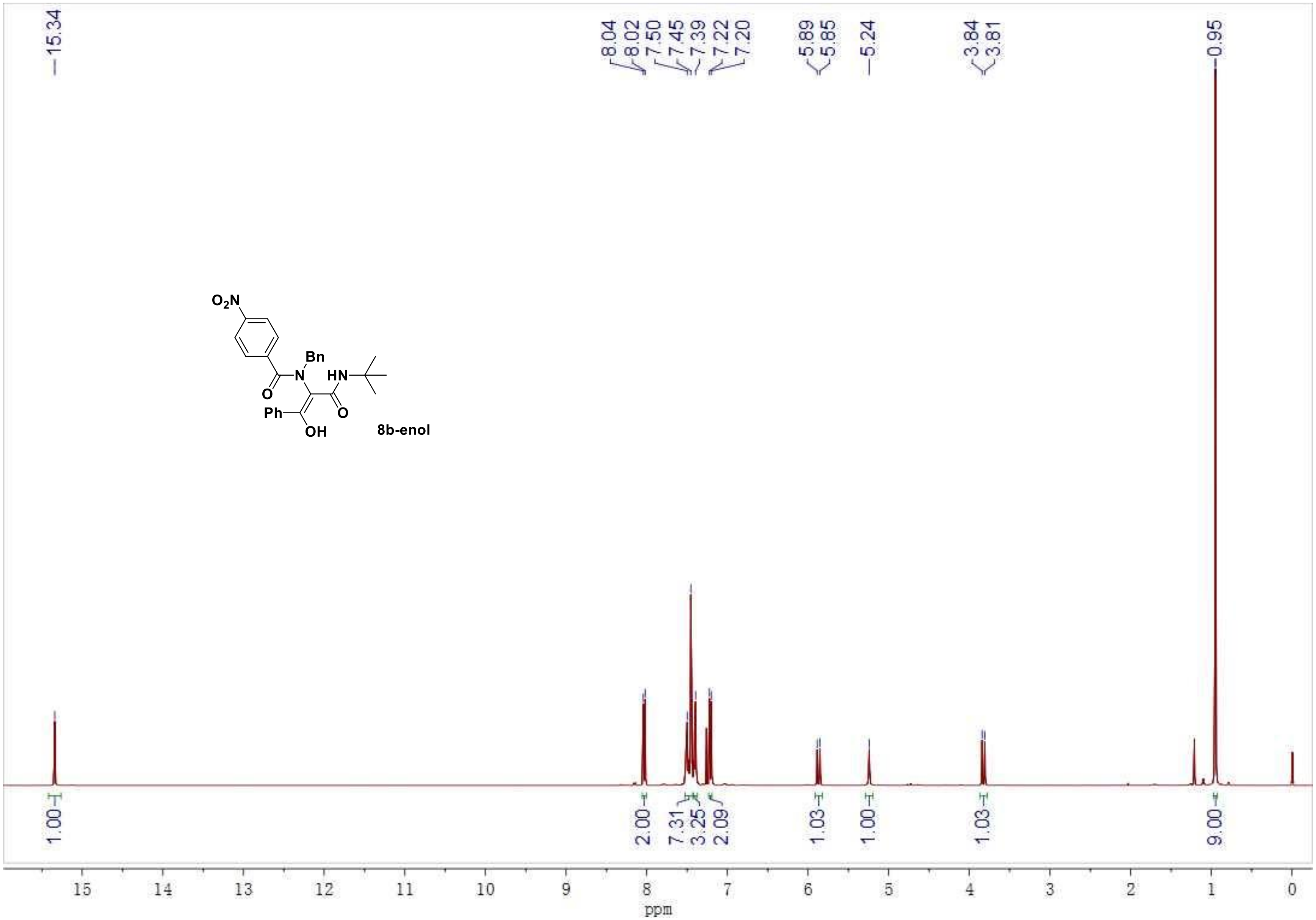

15.34
8.04
8.02
7.50
7.45
7.39
7.22
7.20
5.89
5.85
5.24
3.84
3.81
0.95
O2N
Bn
N
HN
O
O
Ph
OH
1.00
2.00
7.31
3.25
2.09
1.03
1.00
1.03
9.00
15
14
13
12
11
10
9
8
7
6
5
4
3
2
1
0
ppm

8b-enol

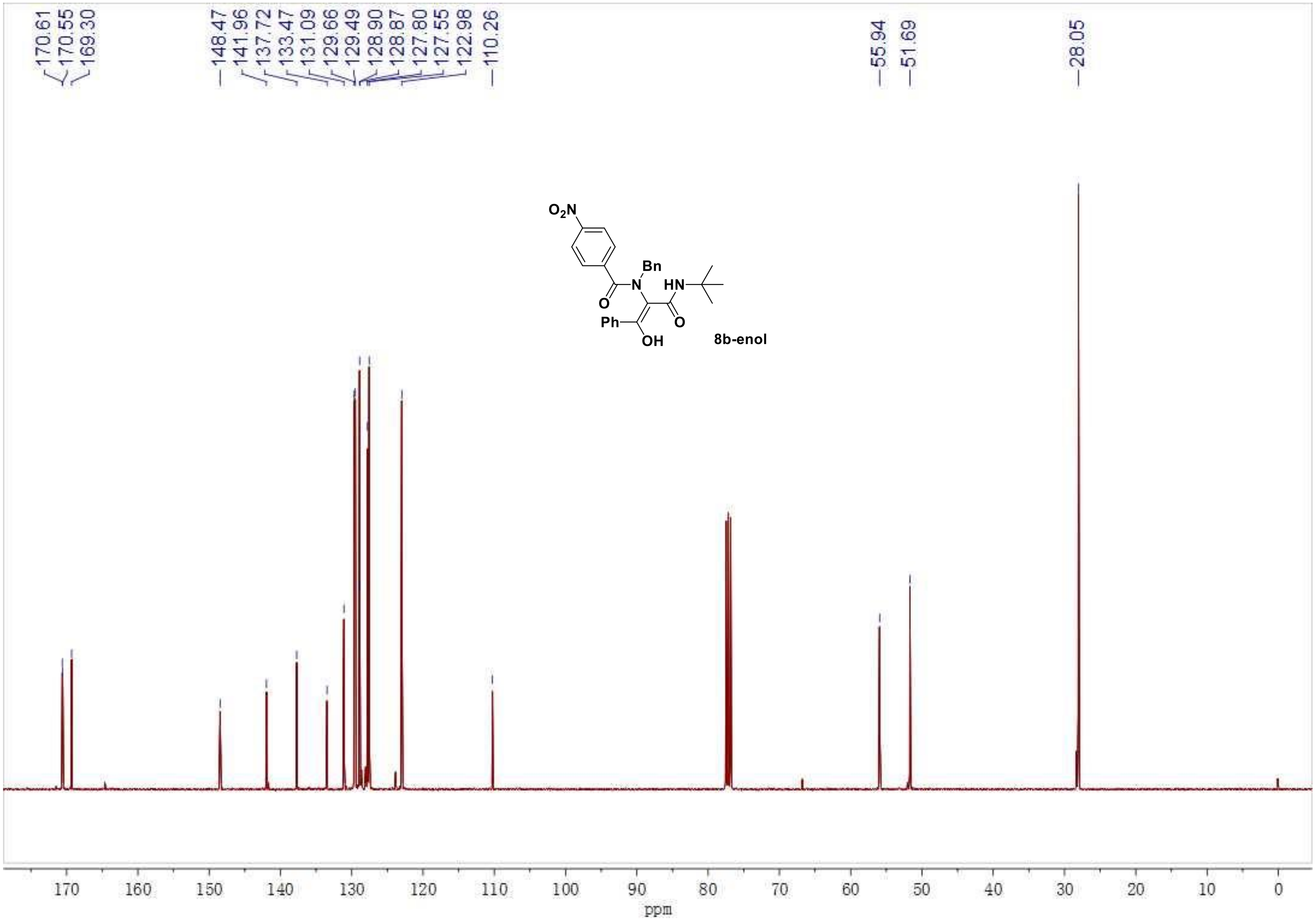
170.61
170.55
169.30
148.47
141.96
137.72
133.47
131.09
129.66
129.49
128.90
128.87
127.80
127.55
122.98
110.26
55.94
51.69
28.05
O2N
Bn
N
HN
O
Ph
OH
O
8b-enol
ppm

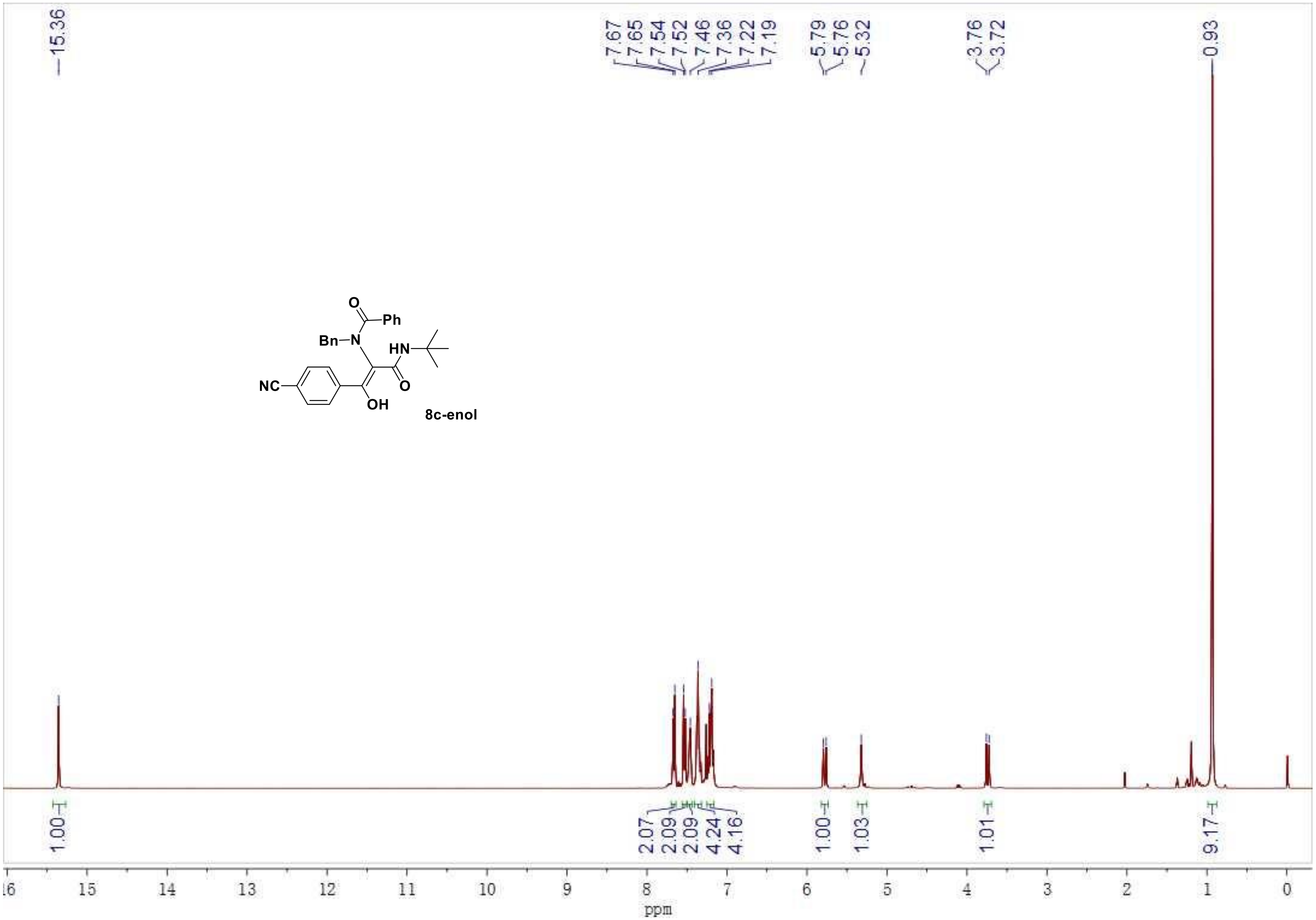
15.36
7.67
7.65
7.54
7.52
7.46
7.36
7.22
7.19
5.79
5.76
5.32
3.76
3.72
0.93
O
Ph
Bn
N
HN
NC
O
OH
8c-enol
1.00
2.07
2.09
2.09
4.24
4.16
1.00
1.03
1.01
9.17
16
15
14
13
12
11
10
9
8
7
6
5
4
3
2
1
0
ppm

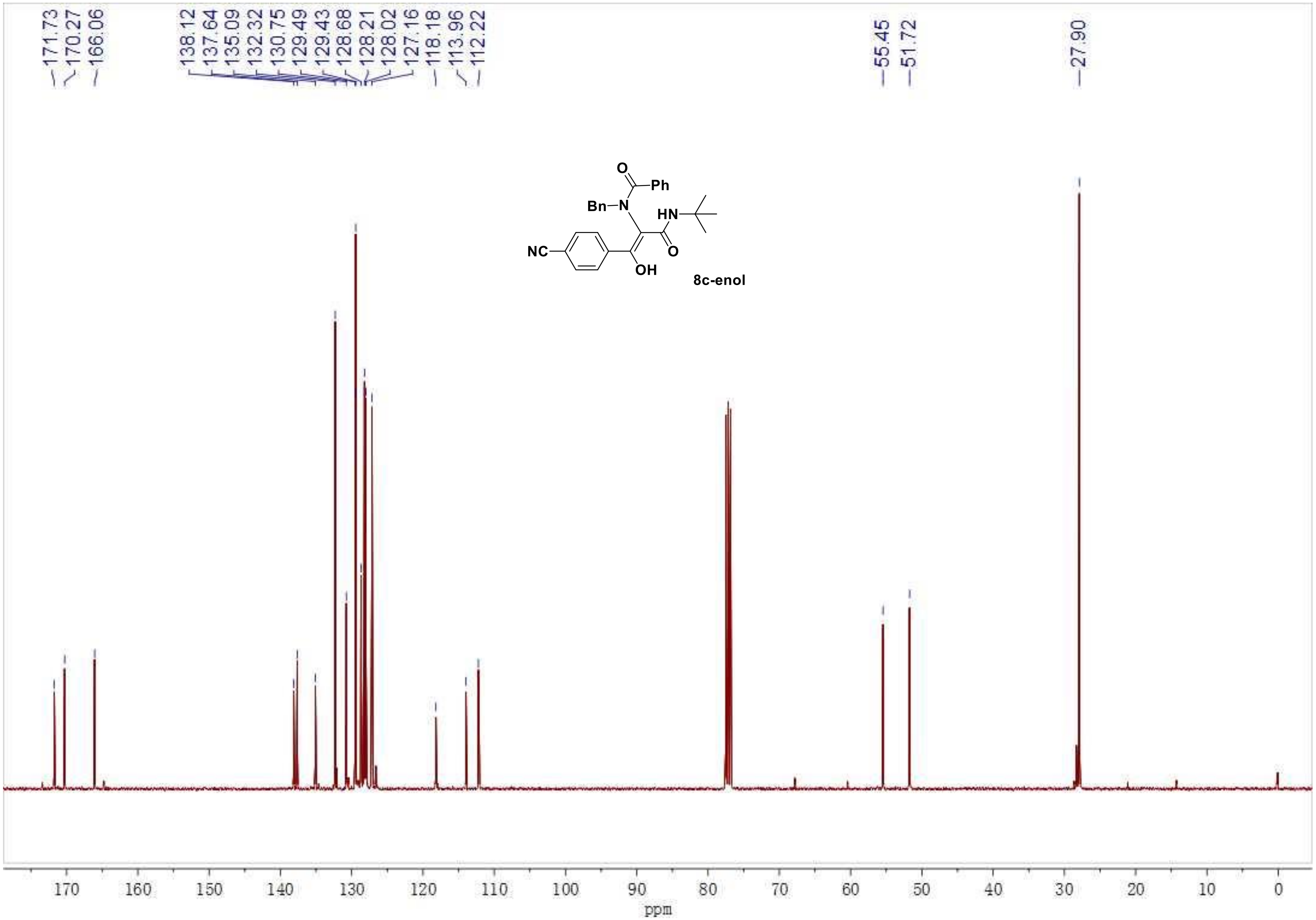

171.73
170.27
166.06
138.12
137.64
135.09
132.32
130.75
129.49
129.43
128.68
128.21
128.02
127.16
118.18
113.96
112.22
55.45
51.72
27.90
O
Ph
Bn
N
HN
NC
O
OH
8c-enol
170 160 150 140 130 120 110 100 90 80 70 60 50 40 30 20 10 0
ppm

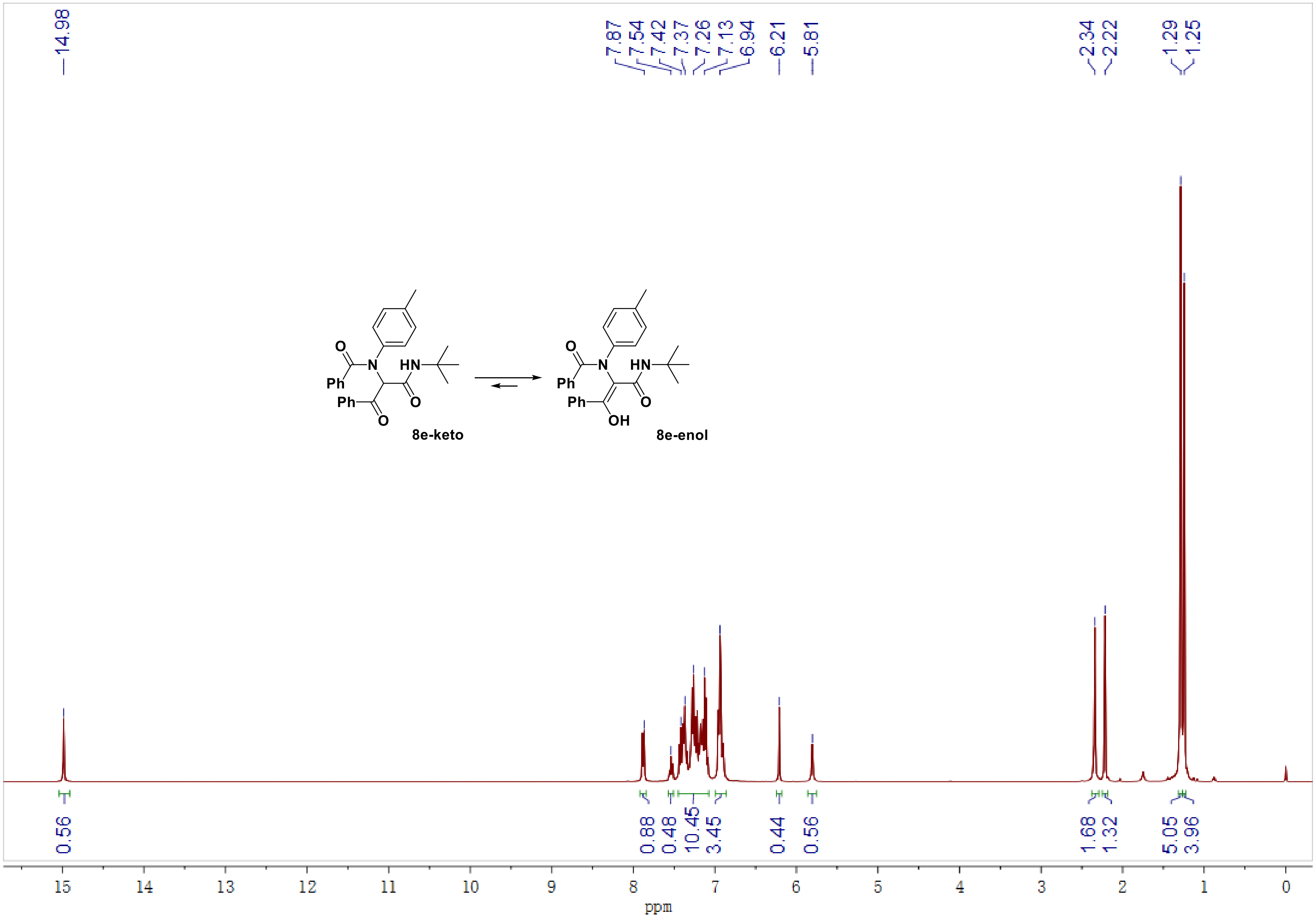

14.98
7.87
7.54
7.42
7.37
7.26
7.13
6.94
6.21
5.81
2.34
2.22
1.29
1.25
O
Ph
N
HN
Ph
O
8e-keto
8e-enol
OH
0.56
0.88
0.48
10.45
3.45
0.44
0.56
1.68
1.32
5.05
3.96
15
14
13
12
11
10
9
8
7
6
5
4
3
2
1
0
ppm

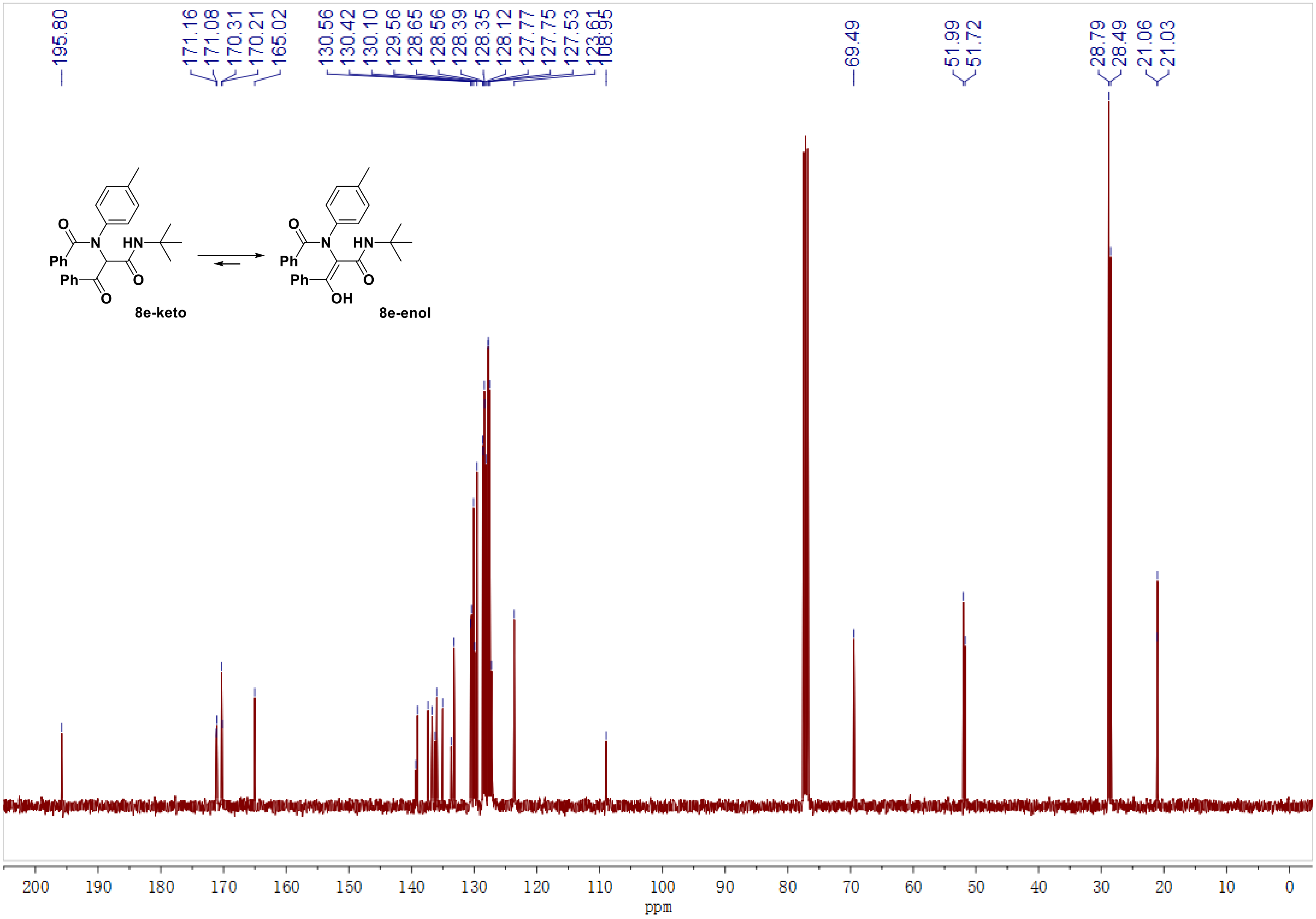
195.80
171.16
171.08
170.31
170.21
165.02
130.56
130.42
130.10
129.56
128.65
128.56
128.39
128.35
128.12
127.77
127.75
127.53
123.61
108.95
69.49
51.99
51.72
28.79
28.49
21.06
21.03
8e-keto
8e-enol
200 190 180 170 160 150 140 130 120 110 100 90 80 70 60 50 40 30 20 10 0
ppm

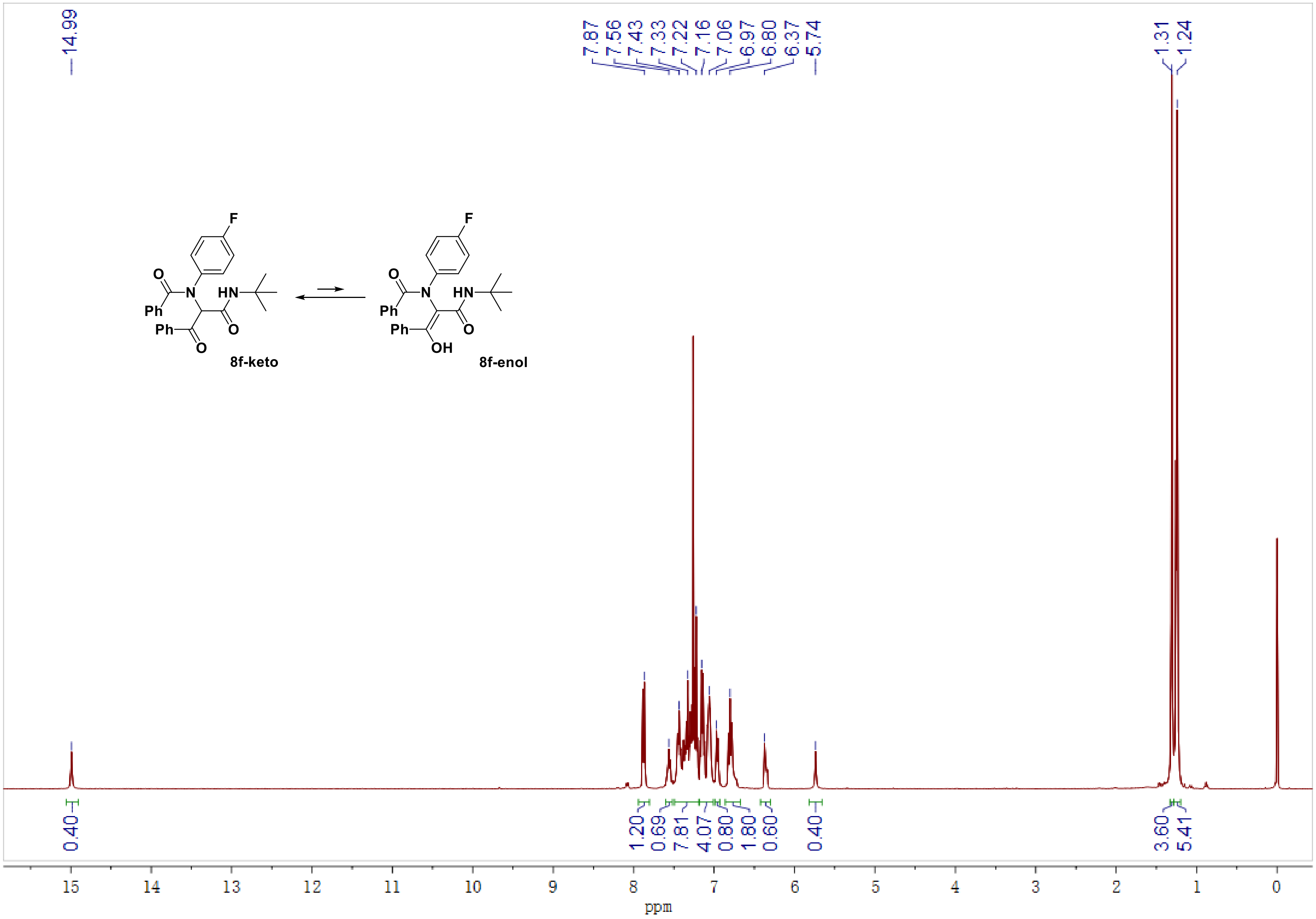
14.99
7.87
7.56
7.43
7.33
7.22
7.16
7.06
6.97
6.80
6.37
5.74
1.31
1.24
F
O
N
HN
Ph
Ph
O
O
8f-keto
F
O
N
HN
Ph
Ph
OH
O
8f-enol
0.40
1.20
0.69
7.81
4.07
0.80
1.80
0.60
0.40
3.60
5.41
15
14
13
12
11
10
9
8
7
6
5
4
3
2
1
0
ppm

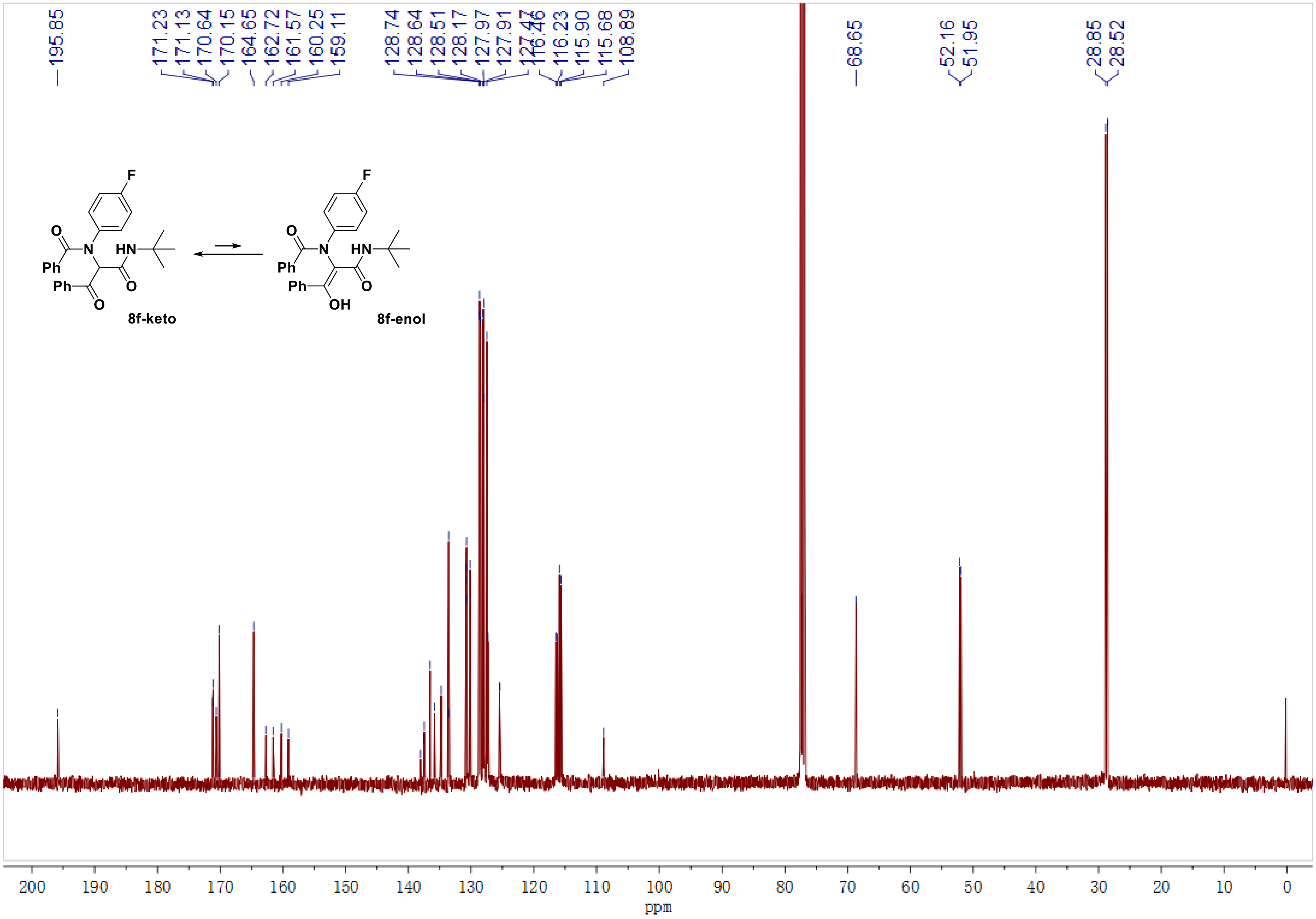
195.85
171.23
171.13
170.64
170.15
164.65
162.72
161.57
160.25
159.11
128.74
128.64
128.51
128.17
127.97
127.91
127.47
116.23
115.90
115.68
108.89
68.65
52.16
51.95
28.85
28.52
F
O
Ph
N
HN
Ph
O
O
8f-keto
OH
8f-enol
200
190
180
170
160
150
140
130
120
110
100
90
80
70
60
50
40
30
20
10
0
ppm

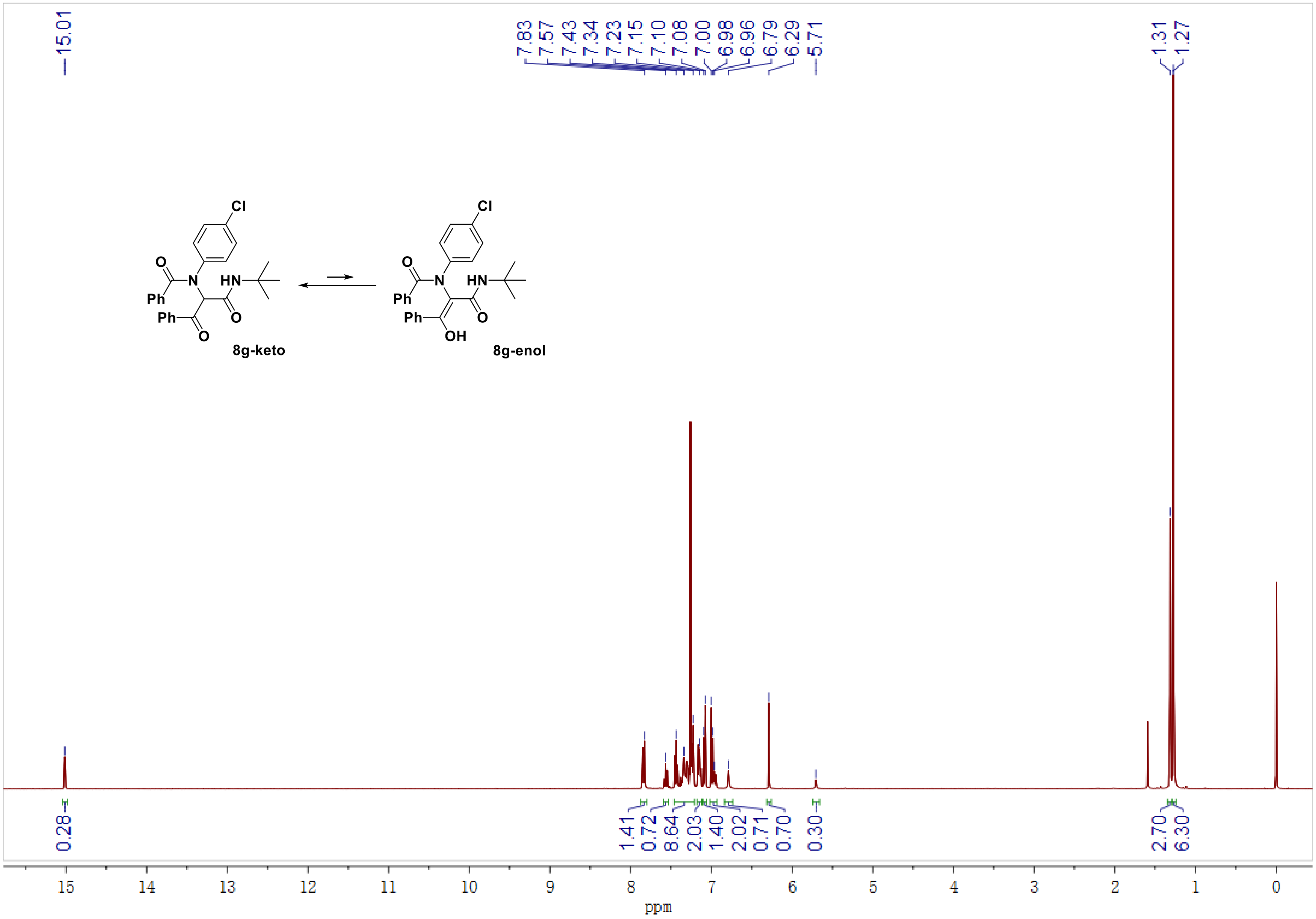

15.01
7.83
7.57
7.43
7.34
7.23
7.15
7.10
7.08
7.00
6.98
6.96
6.79
6.29
5.71
1.31
1.27
Cl
O
N
HN
Ph
Ph
O
O
8g-keto
OH
8g-enol
0.28
1.41
0.72
8.64
2.03
1.40
2.02
0.71
0.70
0.30
2.70
6.30
15
14
13
12
11
10
9
8
7
6
5
4
3
2
1
0
ppm

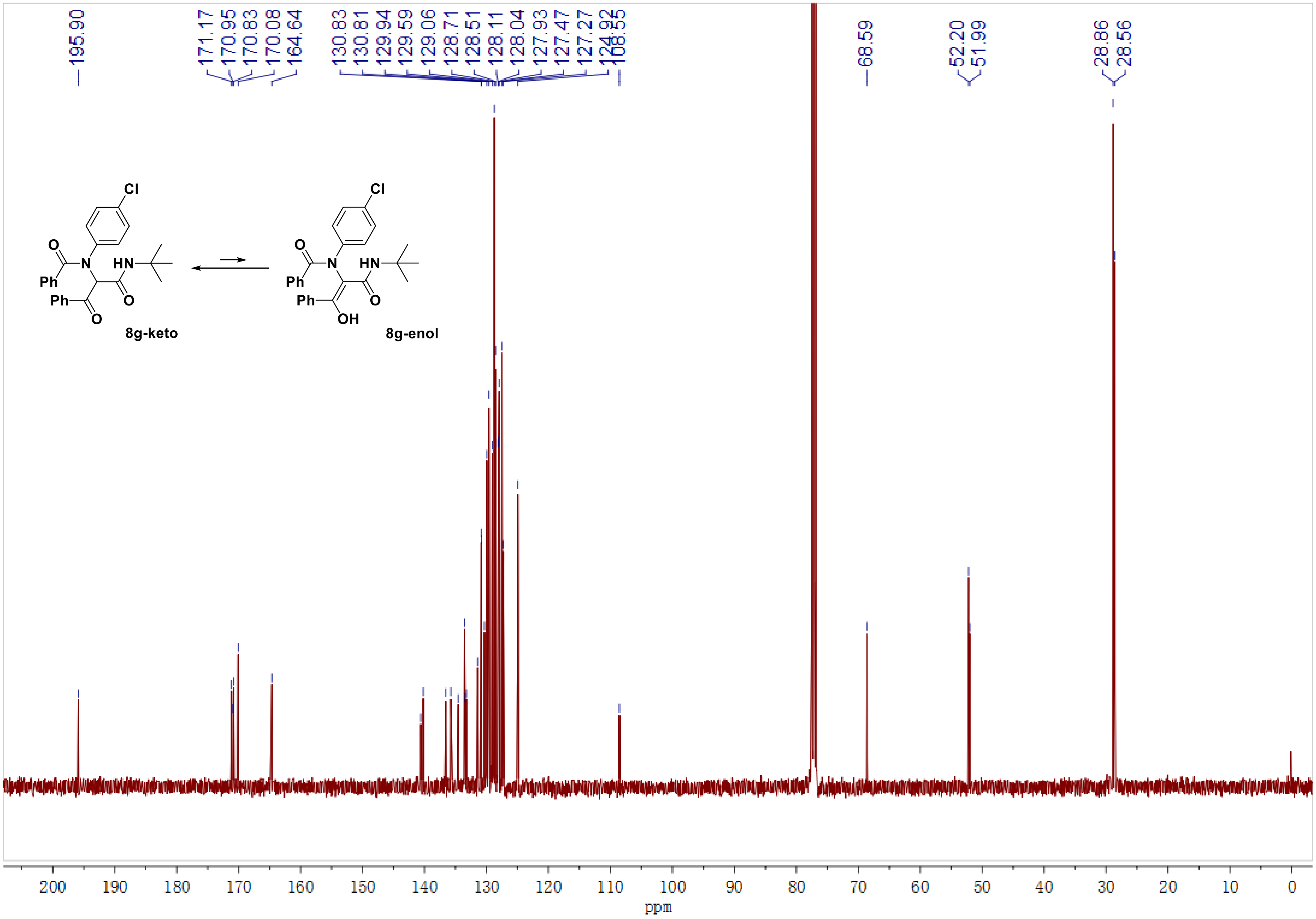
195.90
171.17
170.95
170.83
170.08
164.64
130.83
130.81
129.94
129.59
129.06
128.71
128.51
128.11
128.04
127.93
127.47
127.27
124.92
108.55
68.59
52.20
51.99
28.86
28.56
Cl
O
N
HN
Ph
Ph
O
O
8g-keto
Cl
O
N
HN
Ph
Ph
O
OH
8g-enol
200
190
180
170
160
150
140
130
120
110
100
90
80
70
60
50
40
30
20
10
0
ppm

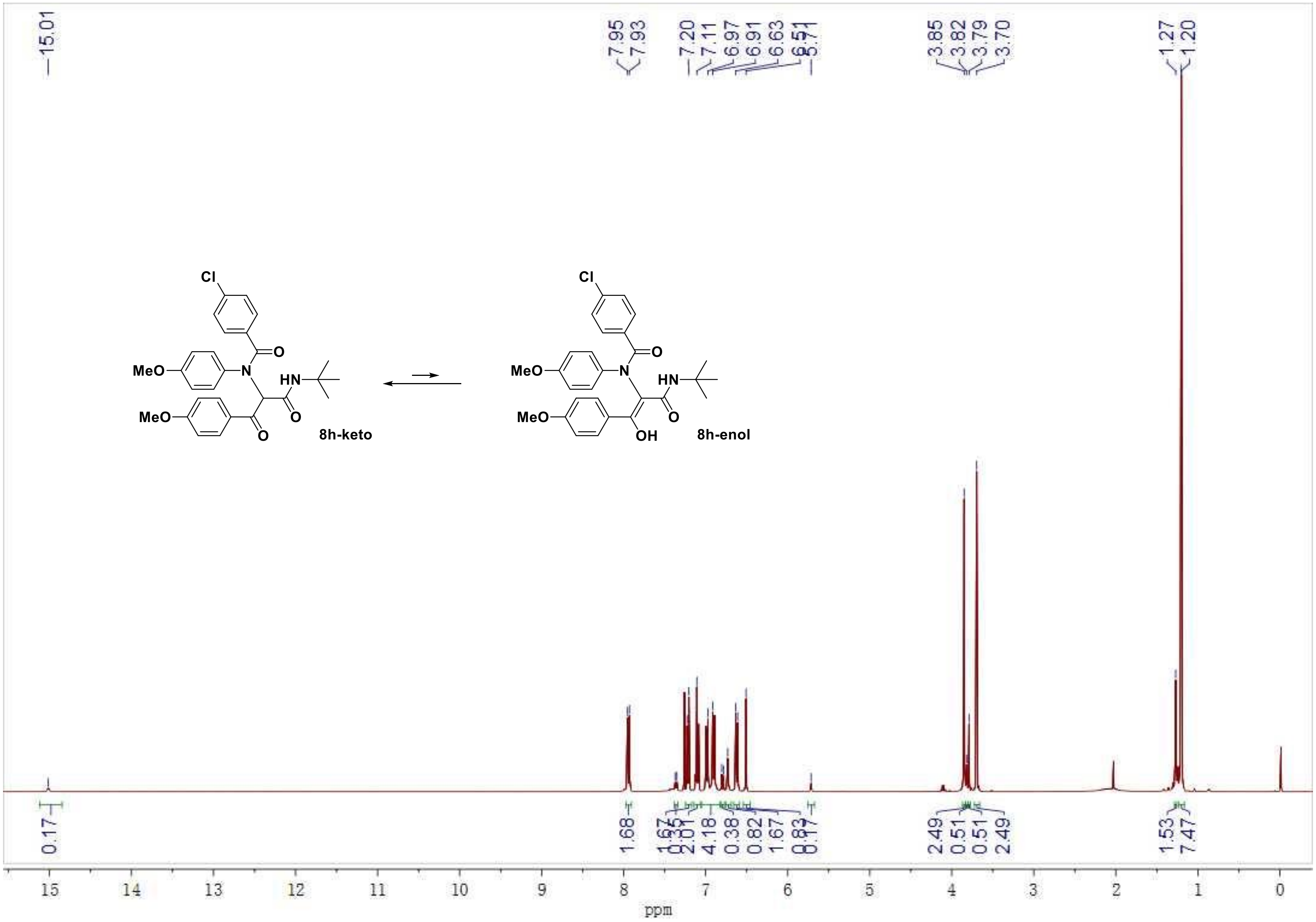
15.01
7.95
7.93
7.20
7.11
6.97
6.91
6.63
6.51
5.71
3.85
3.82
3.79
3.70
1.27
1.20
Cl
MeO
N
O
HN
8h-keto
OH
8h-enol
0.17
1.68
1.67
0.35
2.01
4.18
0.38
0.82
1.67
0.83
0.17
2.49
0.51
0.51
2.49
1.53
7.47
15
14
13
12
11
10
9
8
7
6
5
4
3
2
1
0
ppm

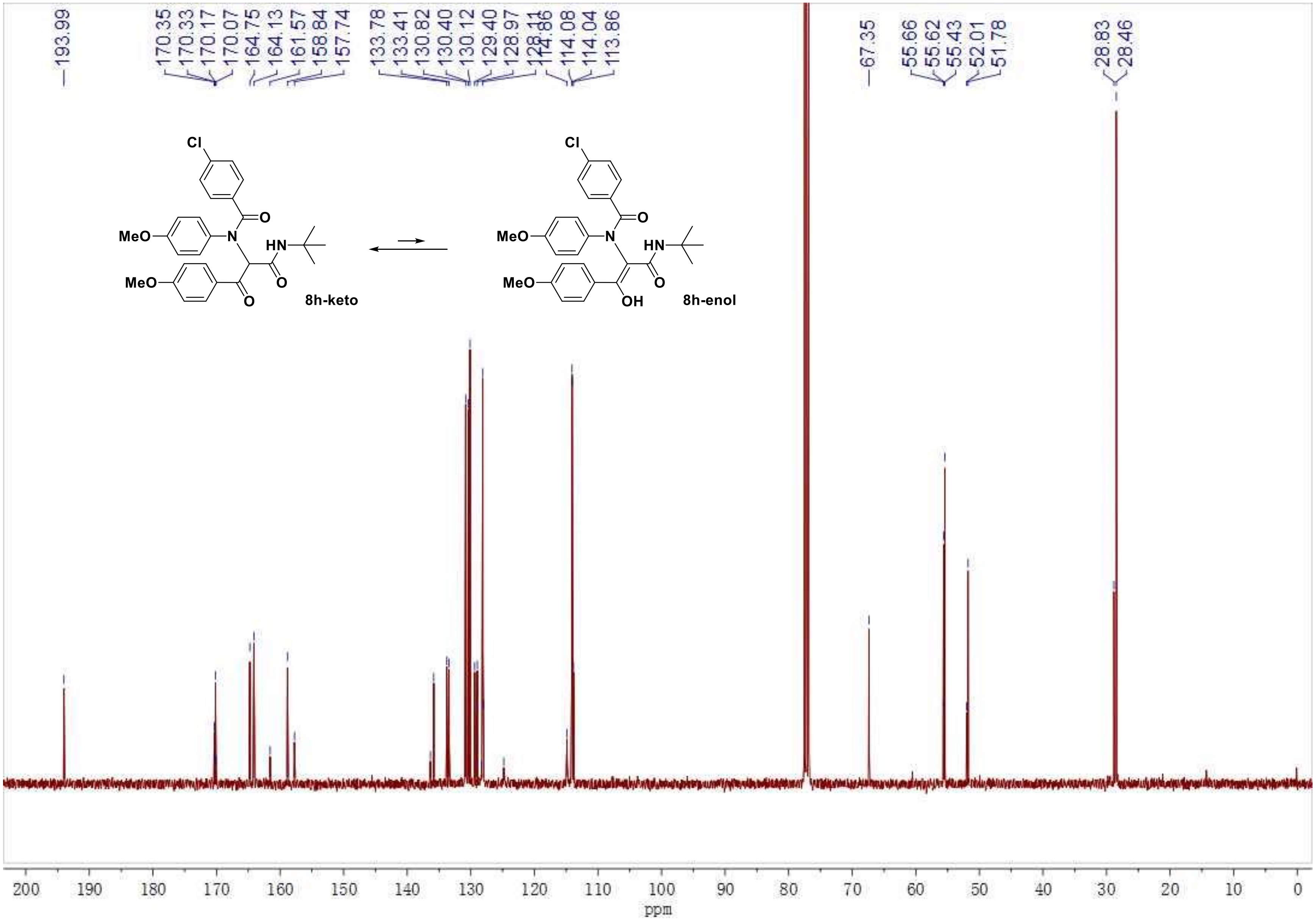

193.99
170.35
170.33
170.17
170.07
164.75
164.13
161.57
158.84
157.74
133.78
133.41
130.82
130.40
130.12
129.40
128.97
114.08
114.04
113.86
67.35
55.66
55.62
55.43
52.01
51.78
28.83
28.46
Cl
MeO
N
O
HN
8h-keto
OH
8h-enol
200
190
180
170
160
150
140
130
120
110
100
90
80
70
60
50
40
30
20
10
0
ppm

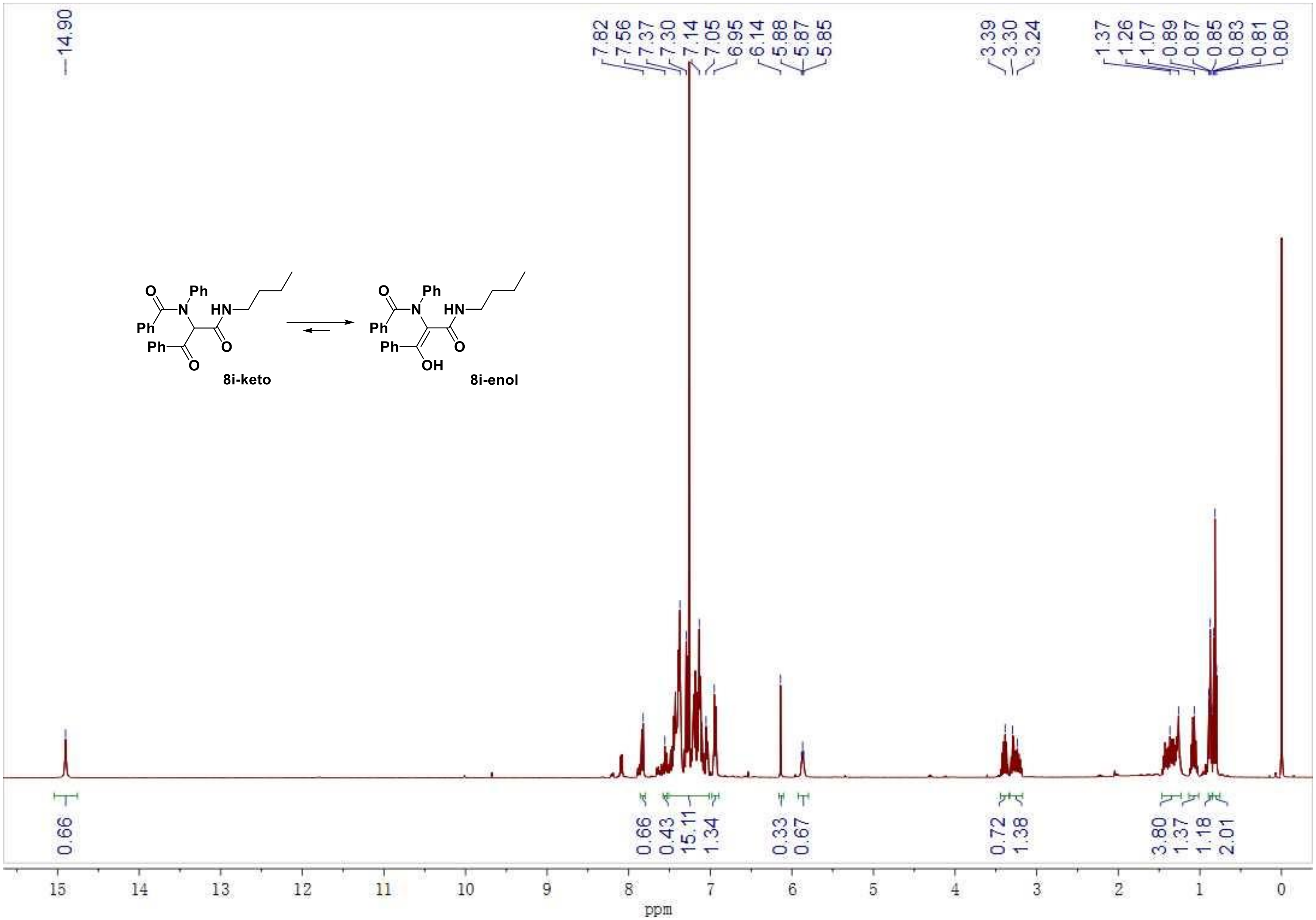

14.90
7.82 7.56 7.37 7.30 7.14 7.05 6.95 6.14 5.88 5.87 5.85
3.39 3.30 3.24
1.37 1.26 1.07 0.89 0.87 0.85 0.83 0.81 0.80
8i-keto
8i-enol
0.66
0.66 0.43 15.11 1.34
0.33 0.67
0.72 1.38
3.80 1.37 1.18 2.01
15 14 13 12 11 10 9 8 7 6 5 4 3 2 1 0
ppm

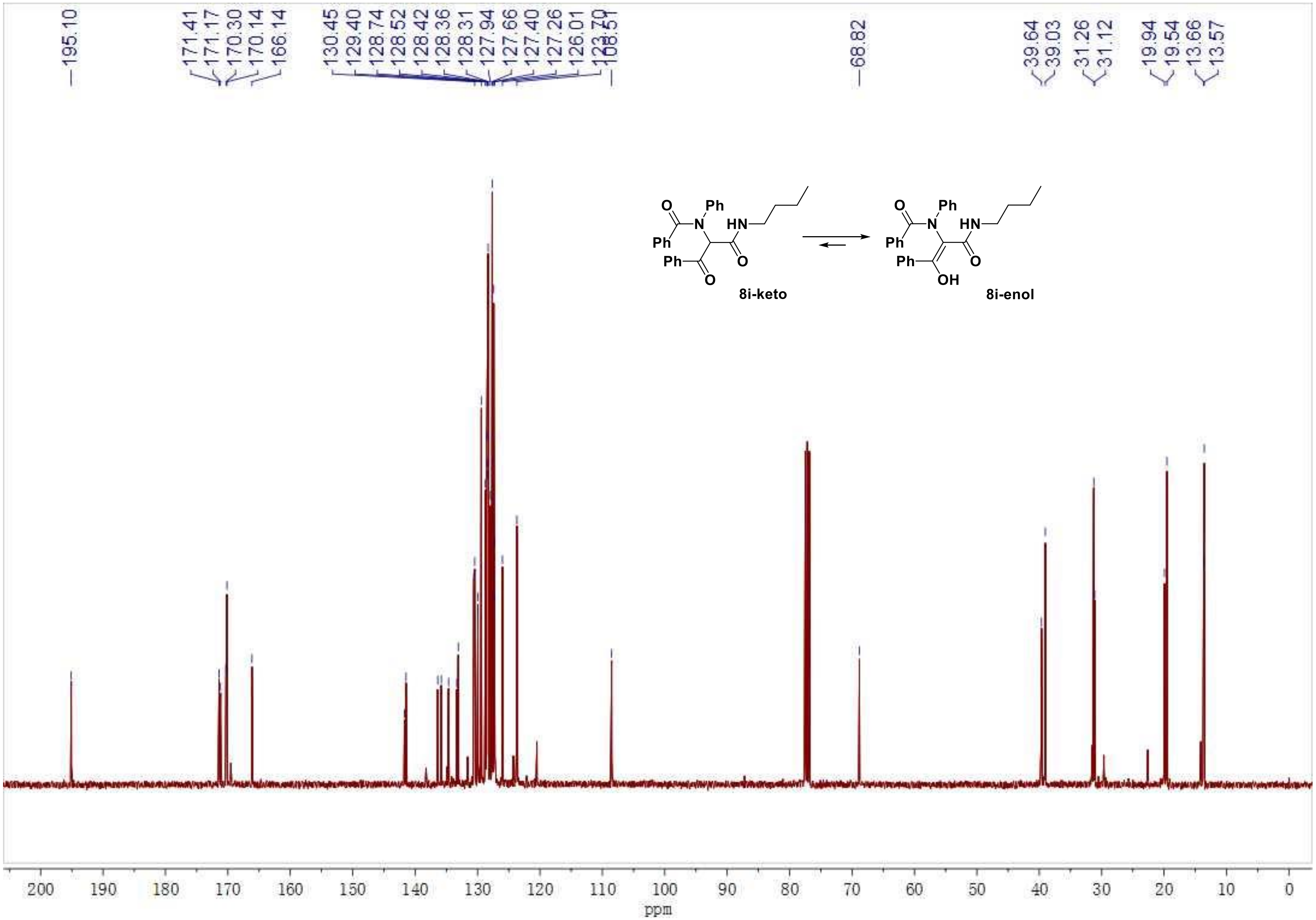
195.10
171.41
171.17
170.30
170.14
166.14
130.45
129.40
128.74
128.52
128.42
128.36
128.31
127.94
127.66
127.40
127.26
126.01
123.70
108.51
68.82
39.64
39.03
31.26
31.12
19.94
19.54
13.66
13.57
8i-keto
8i-enol
ppm

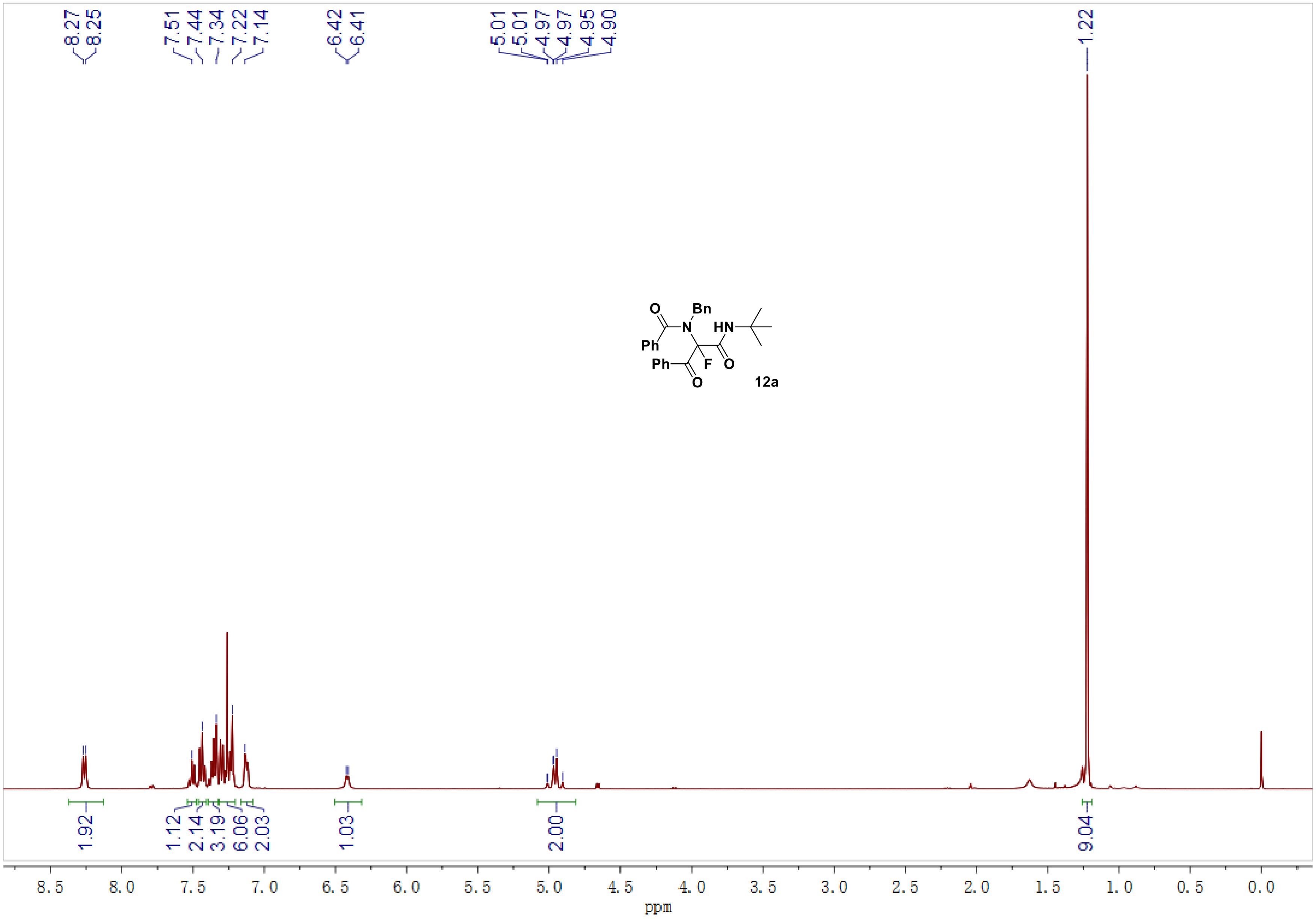
8.27
8.25
7.51
7.44
7.34
7.22
7.14
6.42
6.41
5.01
5.01
4.97
4.97
4.95
4.90
1.22
O
Bn
N
HN
Ph
Ph
F
O
O
12a
1.92
1.12
2.14
3.19
6.06
2.03
1.03
2.00
9.04
8.5
8.0
7.5
7.0
6.5
6.0
5.5
5.0
4.5
4.0
3.5
3.0
2.5
2.0
1.5
1.0
0.5
0.0
ppm

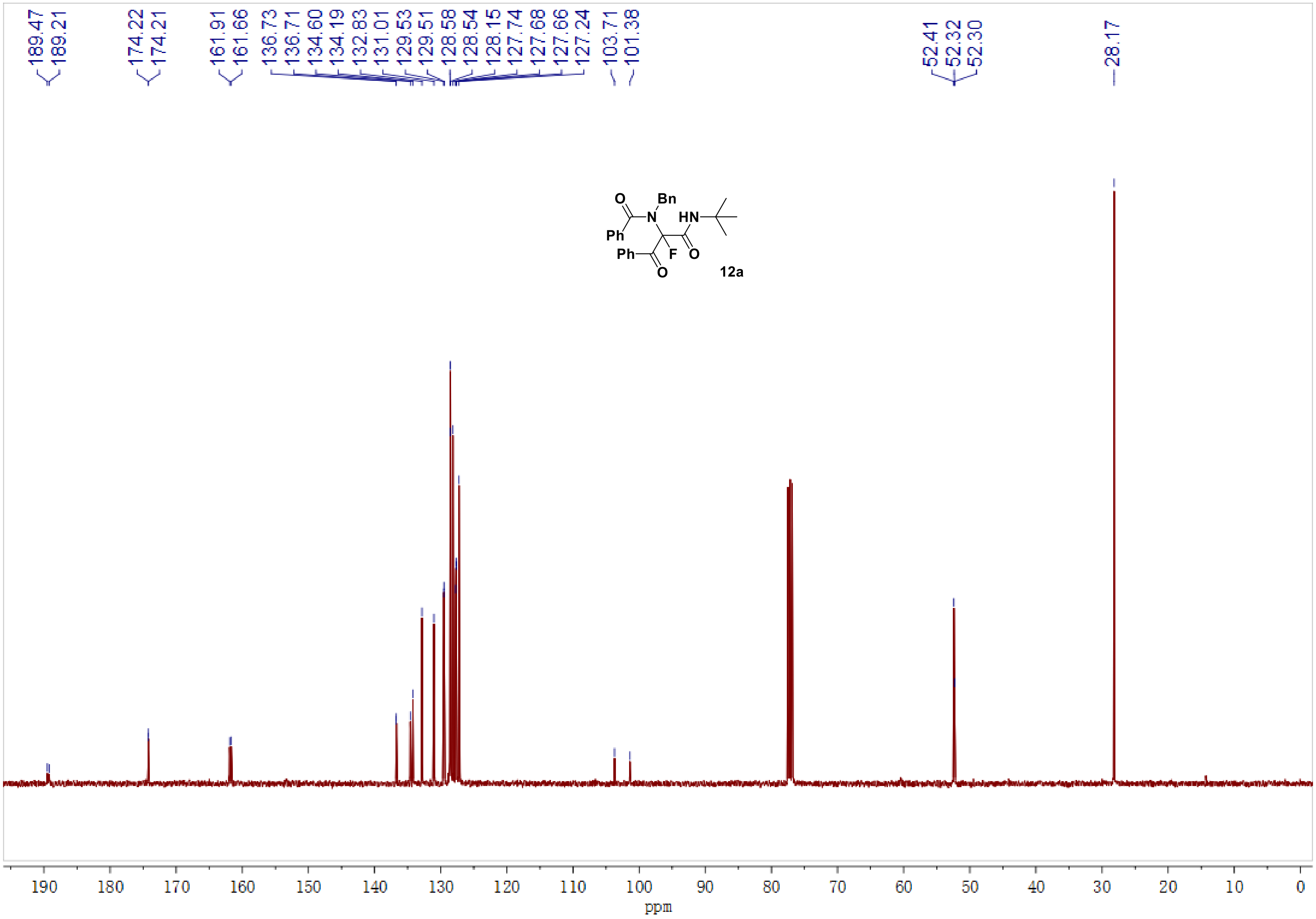
189.47
189.21
174.22
174.21
161.91
161.66
136.73
136.71
134.60
134.19
132.83
131.01
129.53
129.51
128.58
128.54
128.15
127.74
127.68
127.66
127.24
103.71
101.38
52.41
52.32
52.30
28.17
O
Bn
N
HN
Ph
Ph
F
O
O
12a
190 180 170 160 150 140 130 120 110 100 90 80 70 60 50 40 30 20 10 0
ppm

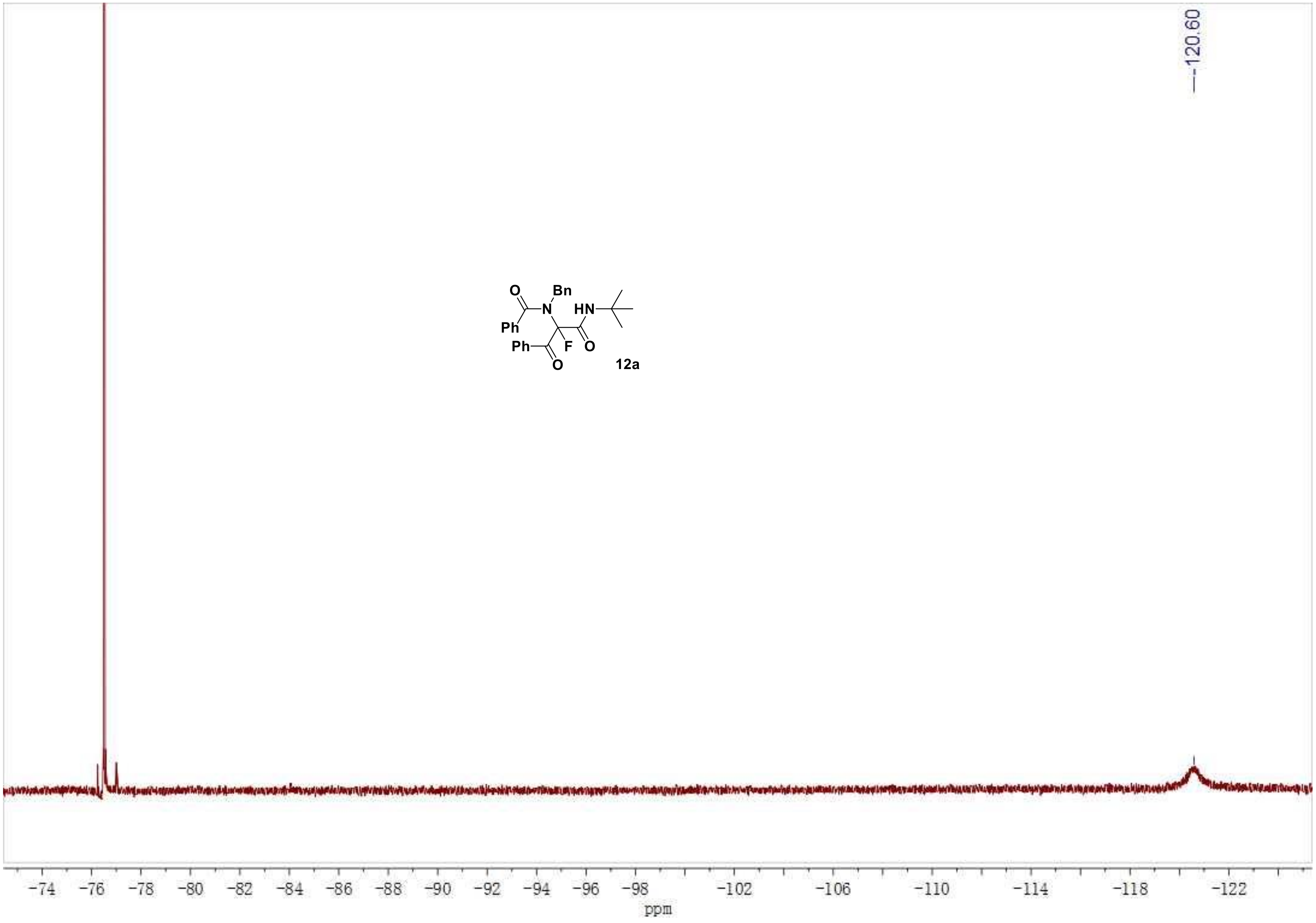
-120.60
O
Bn
N
HN
Ph
Ph
F
O
O
12a
-74
-76
-78
-80
-82
-84
-86
-88
-90
-92
-94
-96
-98
-102
-106
-110
-114
-118
-122
ppm

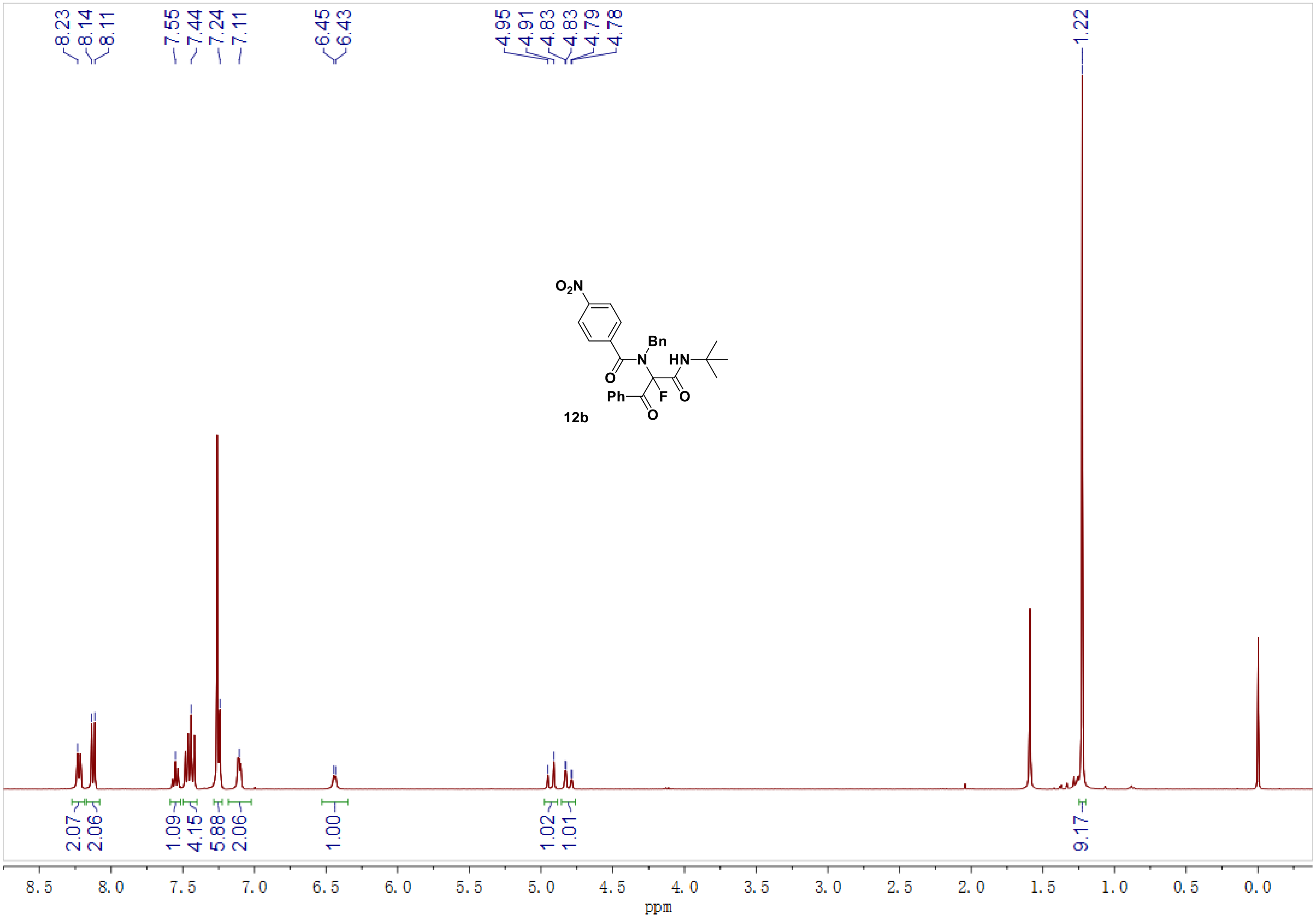
8.23
8.14
8.11
7.55
7.44
7.24
7.11
6.45
6.43
4.95
4.91
4.83
4.83
4.79
4.78
1.22
O_2N
Bn
N
HN
O
Ph
F
O
O
12b
2.07
2.06
1.09
4.15
5.88
2.06
1.00
1.02
1.01
9.17
8.5
8.0
7.5
7.0
6.5
6.0
5.5
5.0
4.5
4.0
3.5
3.0
2.5
2.0
1.5
1.0
0.5
0.0
ppm

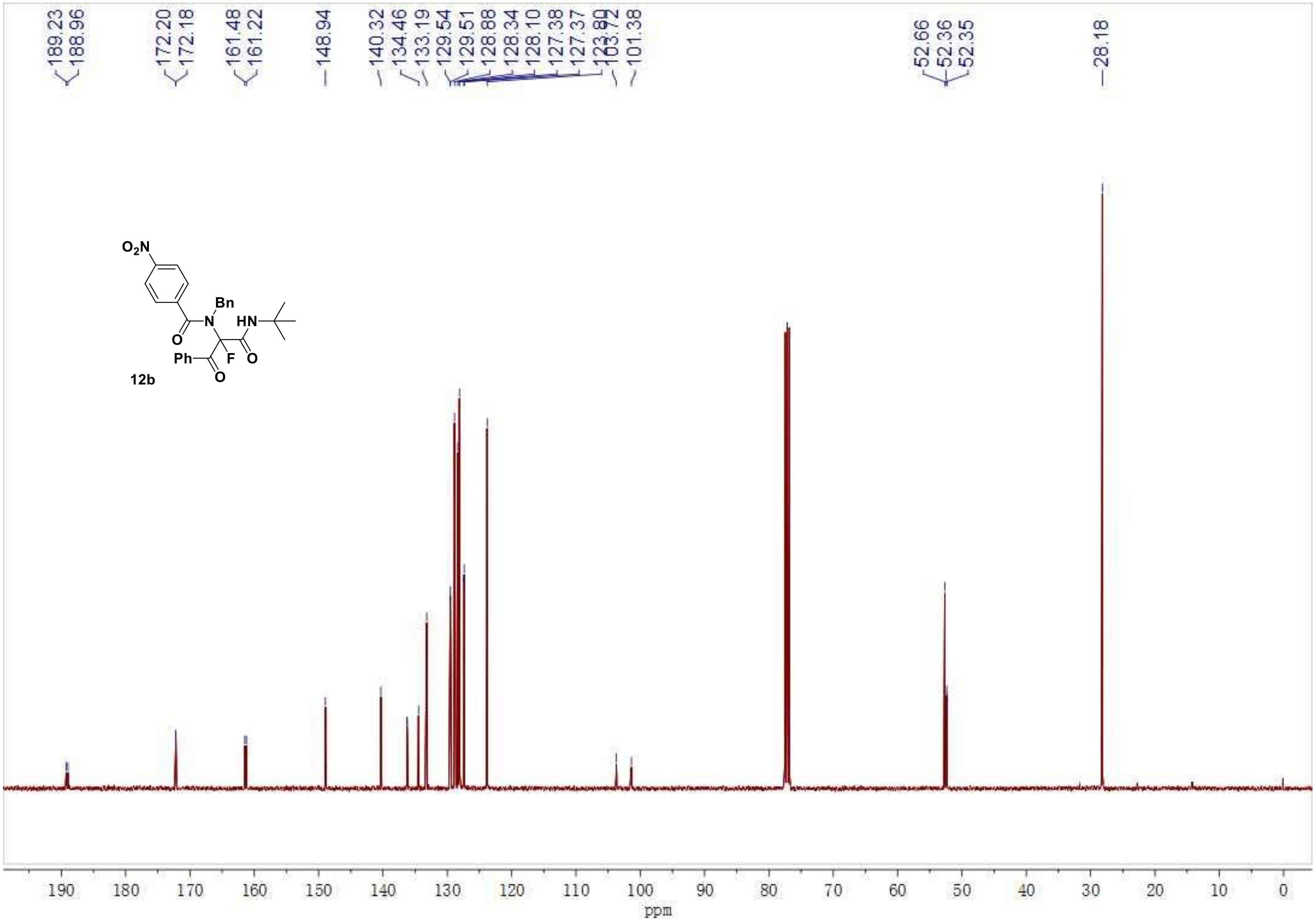
189.23
188.96
172.20
172.18
161.48
161.22
148.94
140.32
134.46
133.19
129.54
129.51
128.88
128.34
128.10
127.38
127.37
123.80
103.72
101.38
52.66
52.36
52.35
28.18
O2N
Bn
N
HN
O
Ph
F
O
O
12b
190 180 170 160 150 140 130 120 110 100 90 80 70 60 50 40 30 20 10 0
ppm

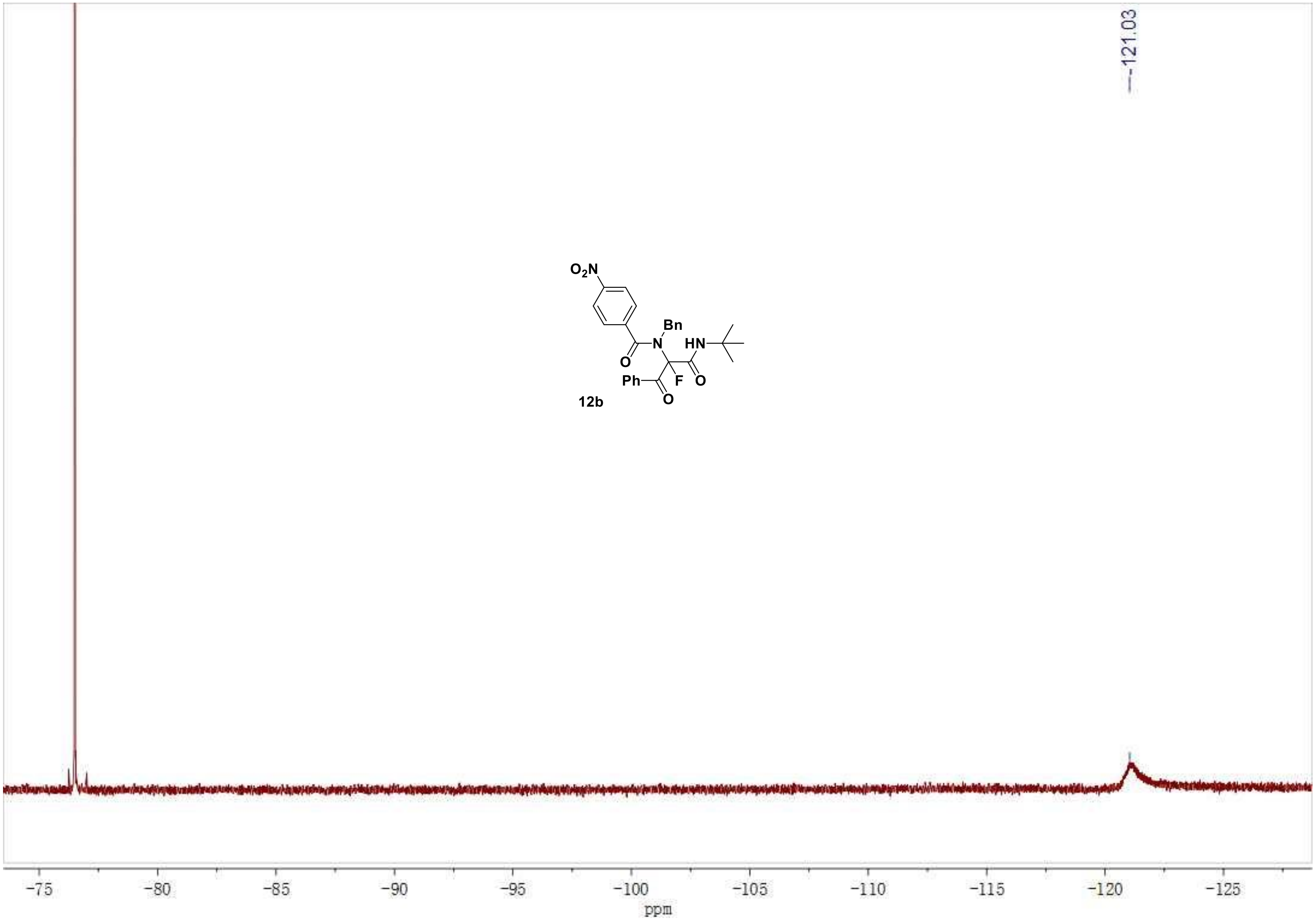

-121.03
O_2N
Bn
N
HN
O
Ph
F
O
O
12b
-75
-80
-85
-90
-95
-100
-105
-110
-115
-120
-125
ppm

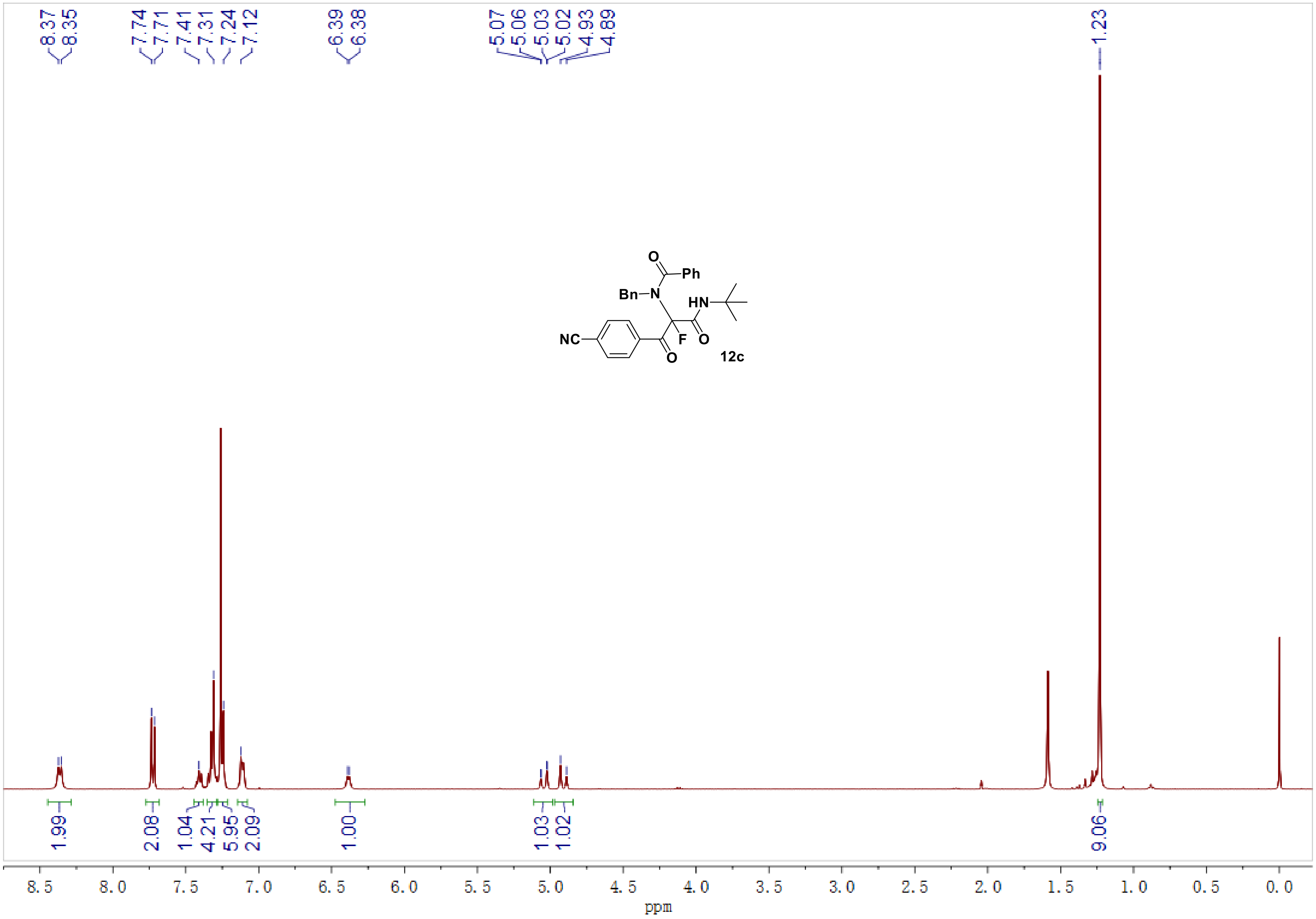
8.37
8.35
7.74
7.71
7.41
7.31
7.24
7.12
6.39
6.38
5.07
5.06
5.03
5.02
4.93
4.89
1.23
O
Ph
Bn
N
HN
NC
F
O
O
12c
1.99
2.08
1.04
4.21
5.95
2.09
1.00
1.03
1.02
9.06
8.5
8.0
7.5
7.0
6.5
6.0
5.5
5.0
4.5
4.0
3.5
3.0
2.5
2.0
1.5
1.0
0.5
0.0
ppm

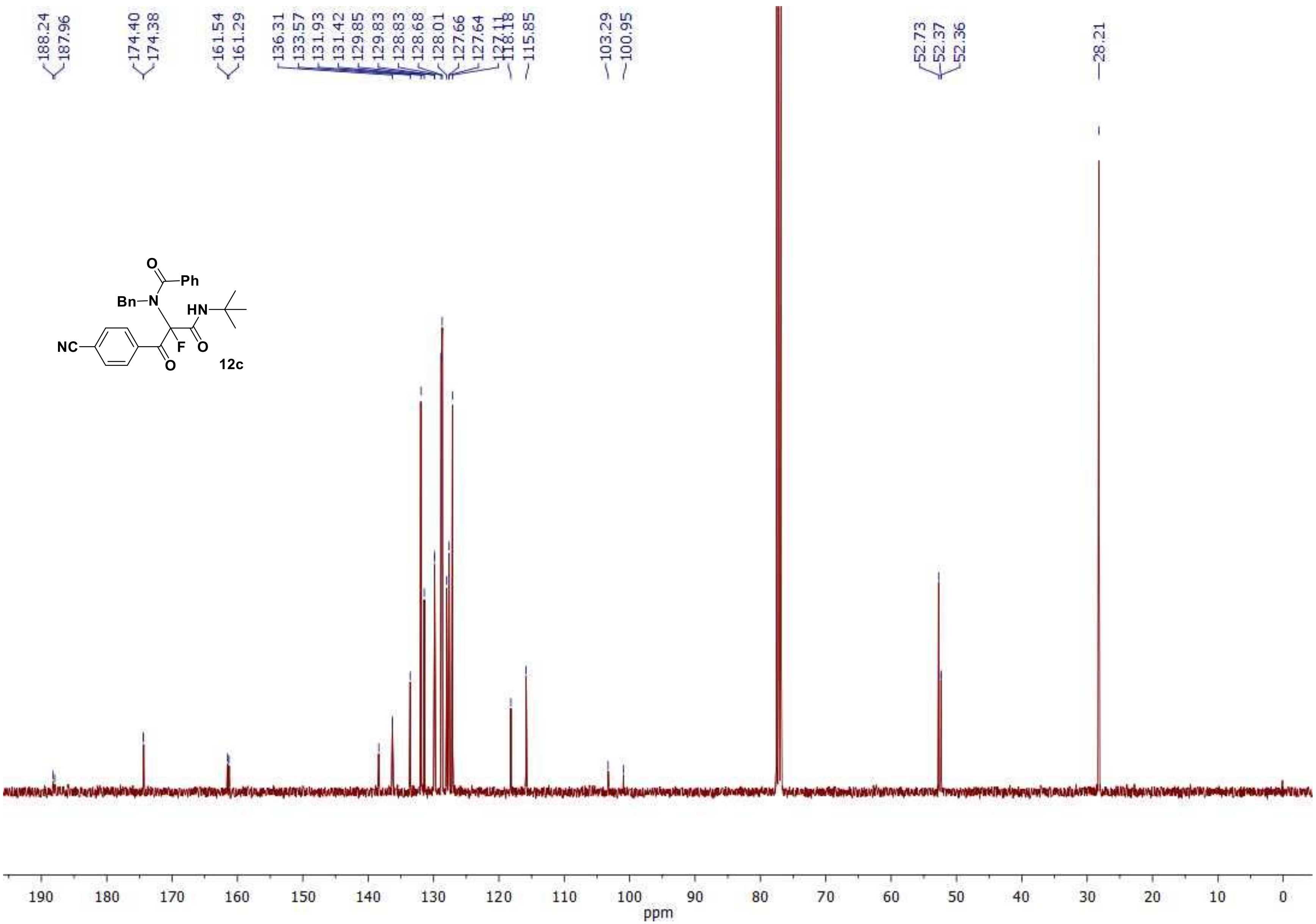
188.24
187.96
174.40
174.38
161.54
161.29
136.31
133.57
131.93
131.42
129.85
129.83
128.83
128.68
128.01
127.66
127.64
127.11
118.18
115.85
103.29
100.95
52.73
52.37
52.36
28.21
12c
190
180
170
160
150
140
130
120
110
100
90
80
70
60
50
40
30
20
10
0
ppm

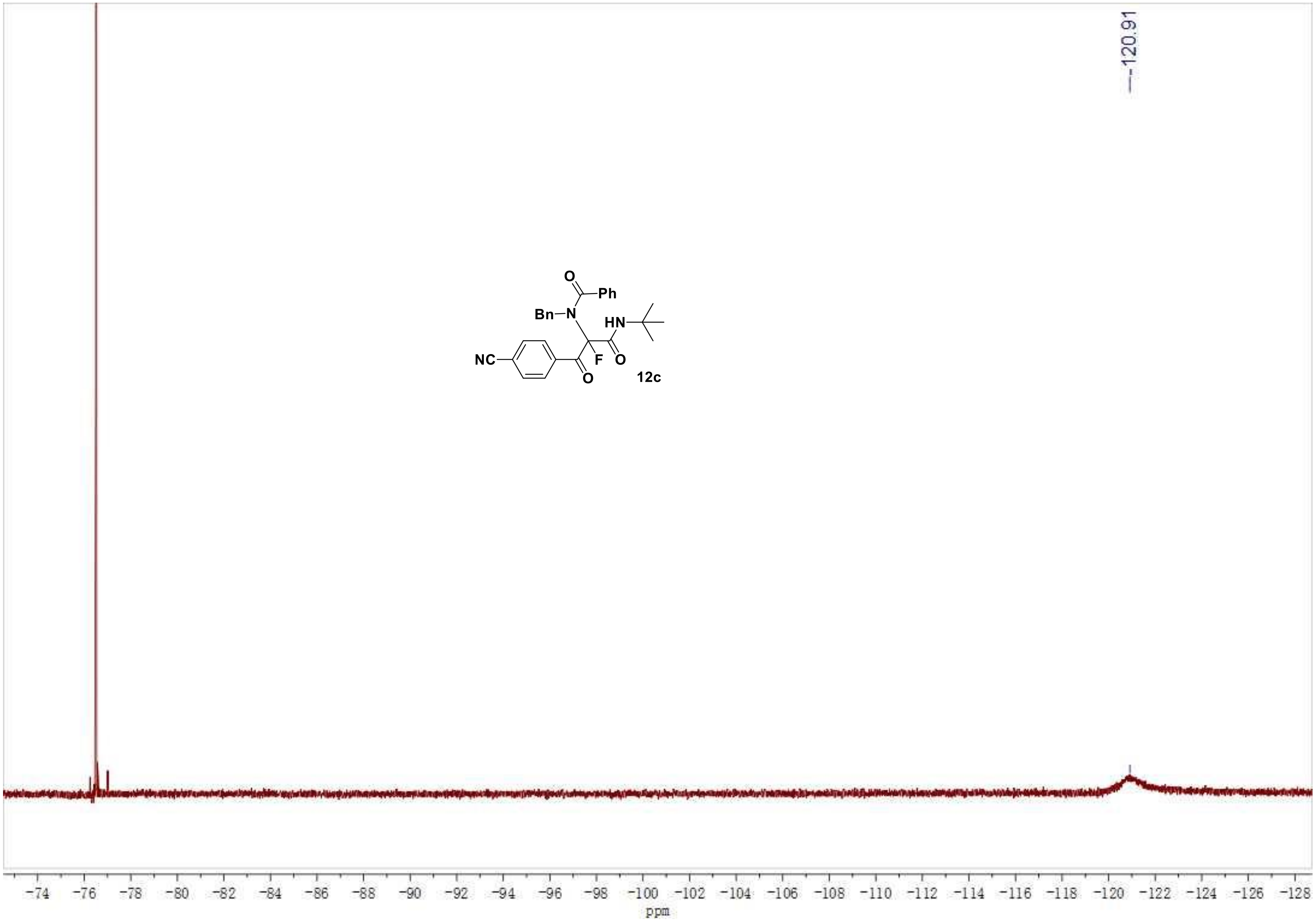
-120.91
O
Ph
Bn
N
HN
NC
F
O
O
12c
-74
-76
-78
-80
-82
-84
-86
-88
-90
-92
-94
-96
-98
-100
-102
-104
-106
-108
-110
-112
-114
-116
-118
-120
-122
-124
-126
-128
ppm

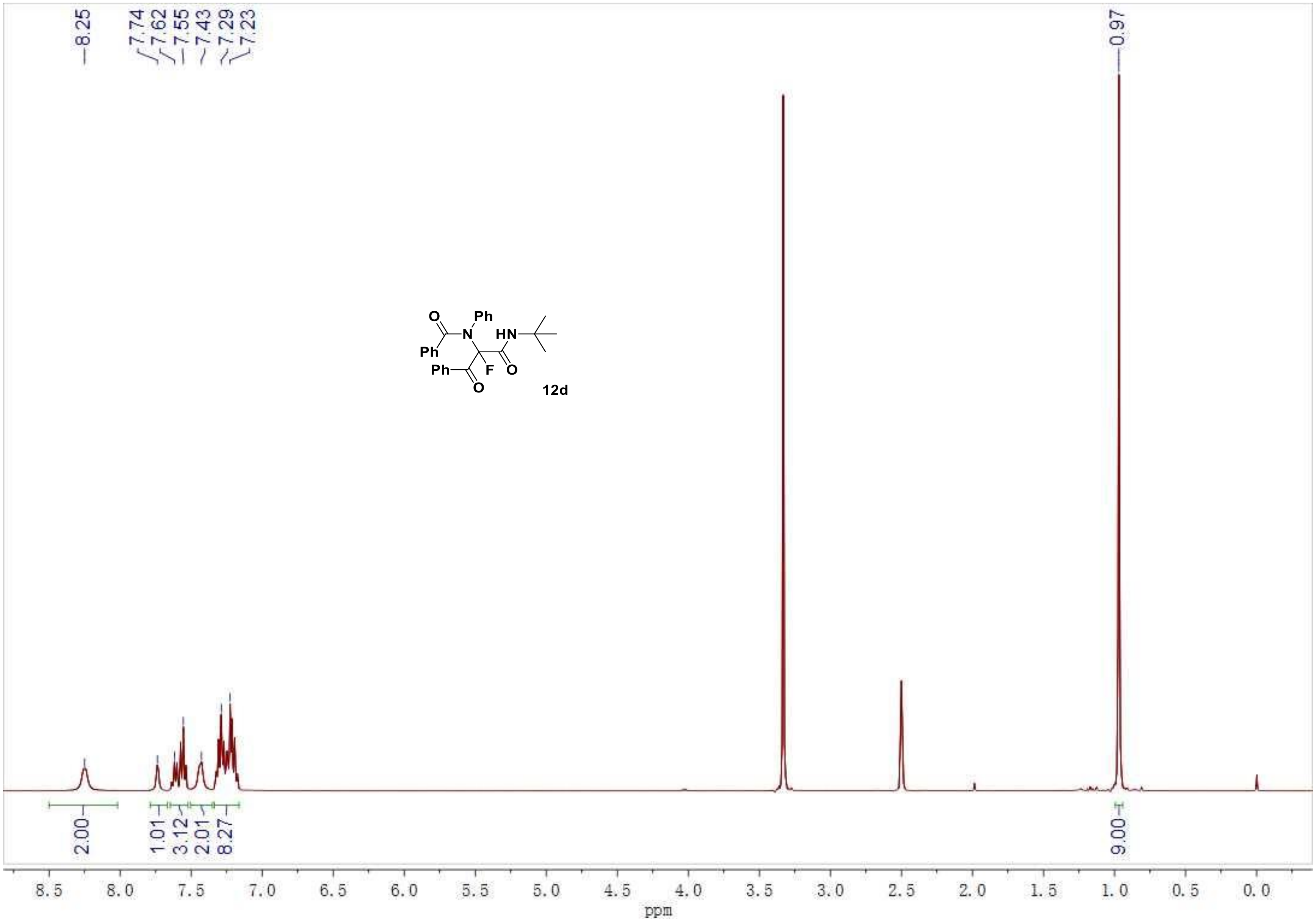

8.25
7.74
7.62
7.55
7.43
7.29
7.23
0.97
O
Ph
N
HN
Ph
Ph
F
O
O
12d
2.00
1.01
3.12
2.01
8.27
9.00
8.5
8.0
7.5
7.0
6.5
6.0
5.5
5.0
4.5
4.0
3.5
3.0
2.5
2.0
1.5
1.0
0.5
0.0
ppm

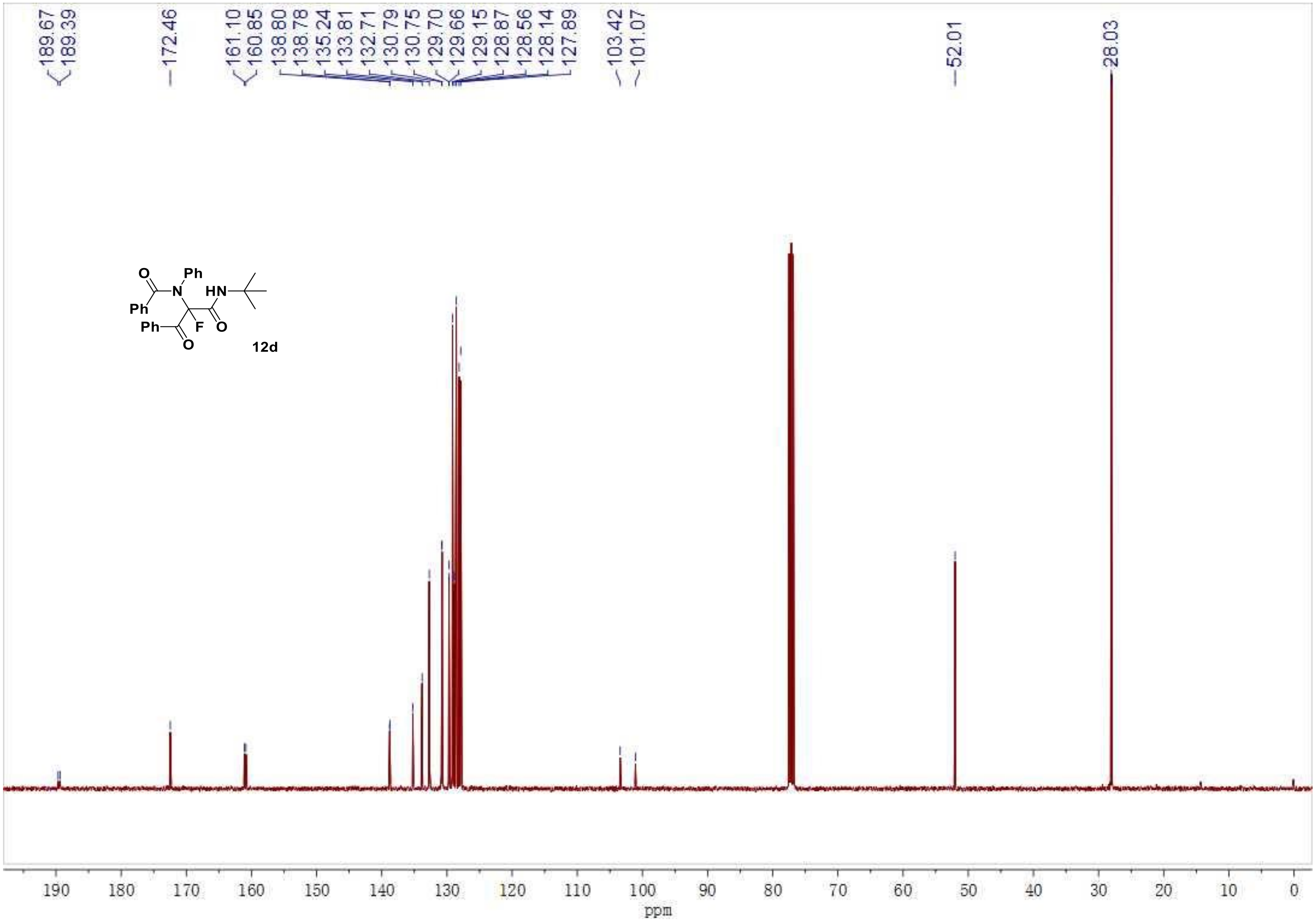
189.67
189.39
172.46
161.10
160.85
138.80
138.78
135.24
133.81
132.71
130.79
130.75
129.70
129.66
129.15
128.87
128.56
128.14
127.89
103.42
101.07
52.01
28.03
O
Ph
N
HN
Ph
F
O
Ph
O
12d
190
180
170
160
150
140
130
120
110
100
90
80
70
60
50
40
30
20
10
0
ppm

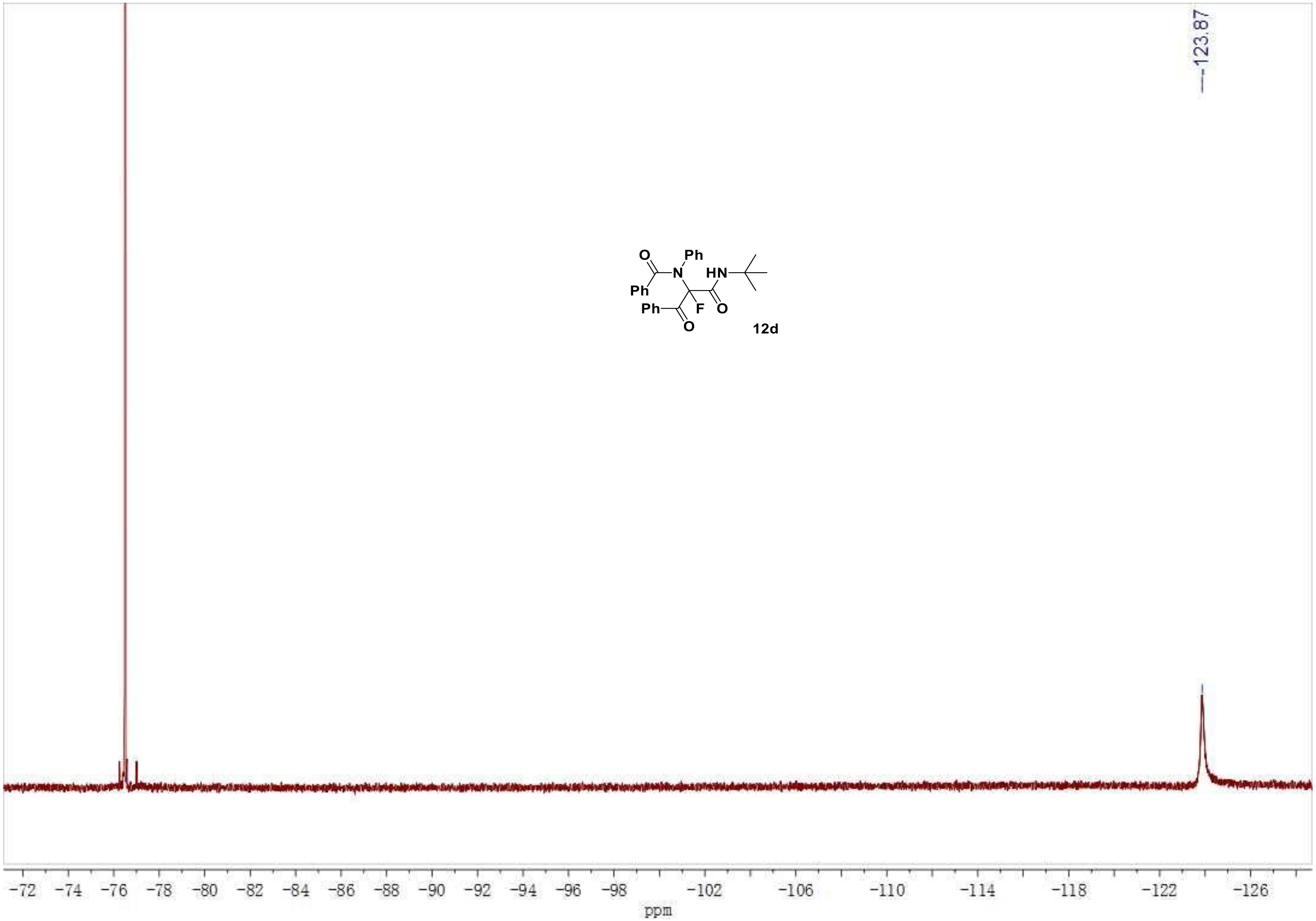

-123.87
Ph
O
N
HN
Ph
Ph
F
O
O
12d
-72
-74
-76
-78
-80
-82
-84
-86
-88
-90
-92
-94
-96
-98
-102
-106
-110
-114
-118
-122
-126
ppm

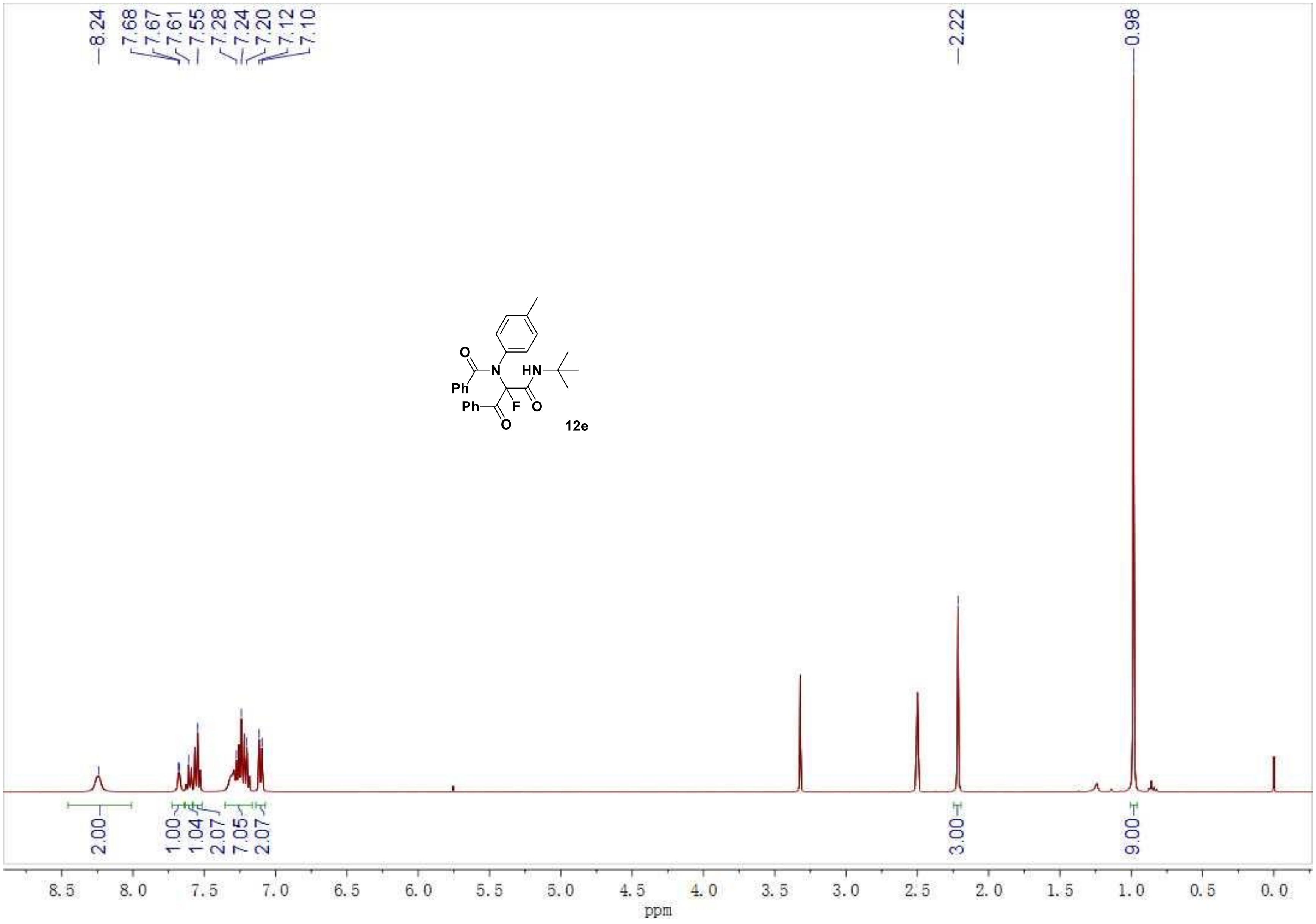

8.24
7.68
7.67
7.61
7.55
7.28
7.24
7.20
7.12
7.10
2.22
0.98
O
N
HN
Ph
Ph
F
O
O
12e
2.00
1.00
1.04
2.07
7.05
2.07
3.00
9.00
8.5
8.0
7.5
7.0
6.5
6.0
5.5
5.0
4.5
4.0
3.5
3.0
2.5
2.0
1.5
1.0
0.5
0.0
ppm

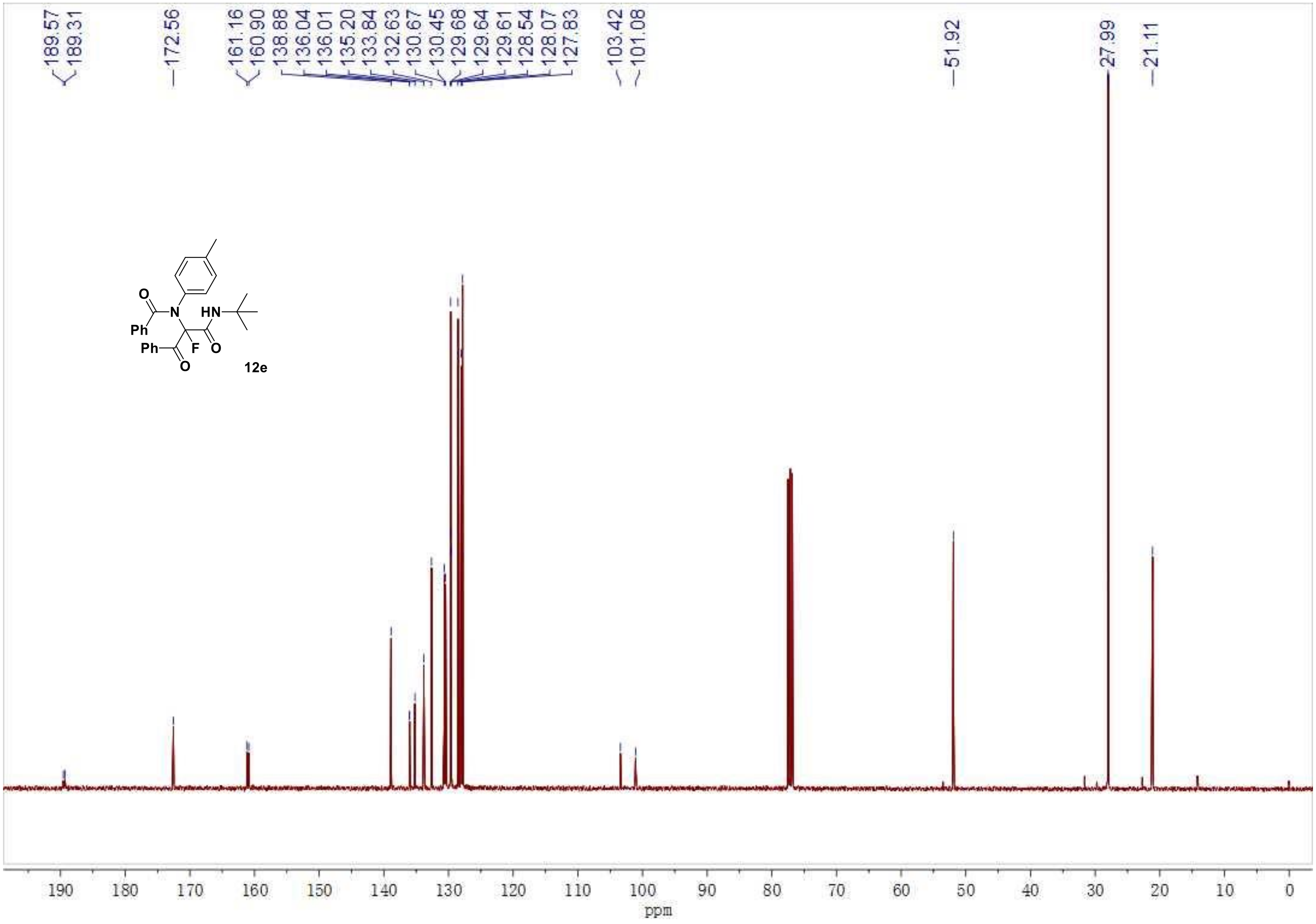
189.57
189.31
172.56
161.16
160.90
138.88
136.04
136.01
135.20
133.84
132.63
130.67
130.45
129.68
129.64
129.61
128.54
128.07
127.83
103.42
101.08
51.92
27.99
21.11
O
N
HN
Ph
Ph
F
O
O
12e
190 180 170 160 150 140 130 120 110 100 90 80 70 60 50 40 30 20 10 0
ppm

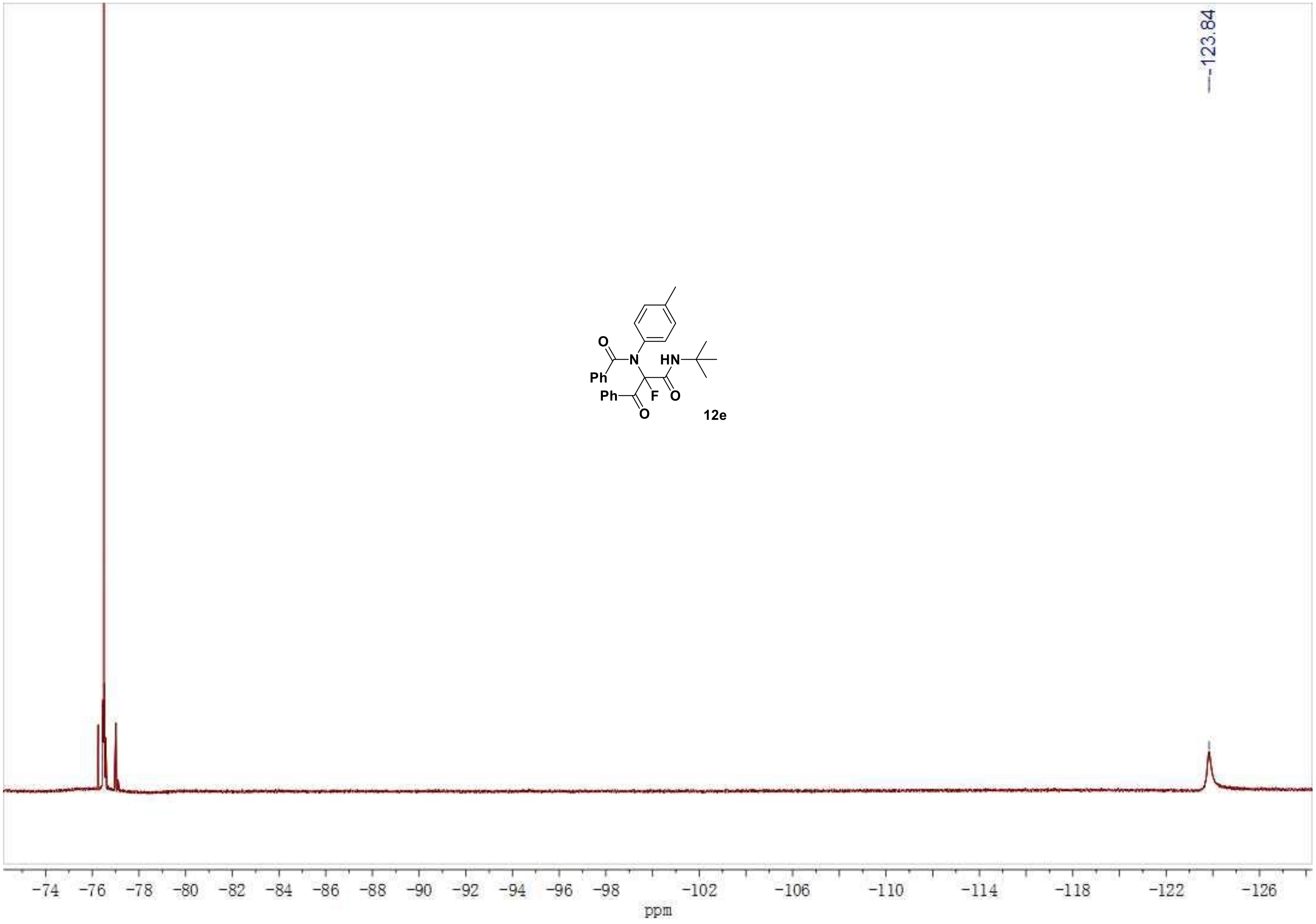
-123.84
O
N
Ph
HN
Ph
F
O
O
12e
-74 -76 -78 -80 -82 -84 -86 -88 -90 -92 -94 -96 -98 -102 -106 -110 -114 -118 -122 -126
ppm

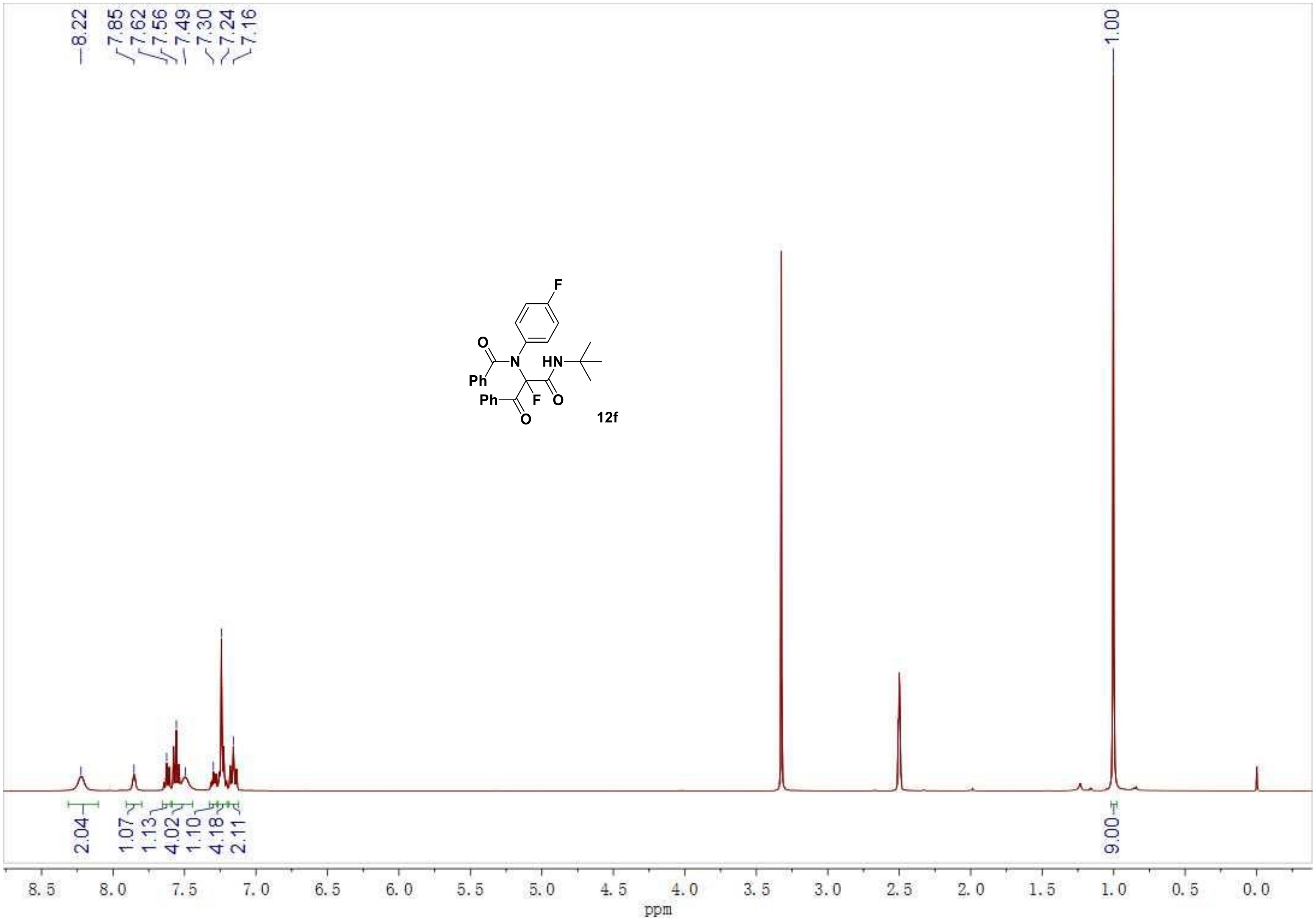

8.22
7.85
7.62
7.56
7.49
7.30
7.24
7.16
1.00
F
O
N
HN
Ph
Ph
F
O
O
12f
2.04
1.07
1.13
4.02
1.10
4.18
2.11
9.00
8.5
8.0
7.5
7.0
6.5
6.0
5.5
5.0
4.5
4.0
3.5
3.0
2.5
2.0
1.5
1.0
0.5
0.0
ppm

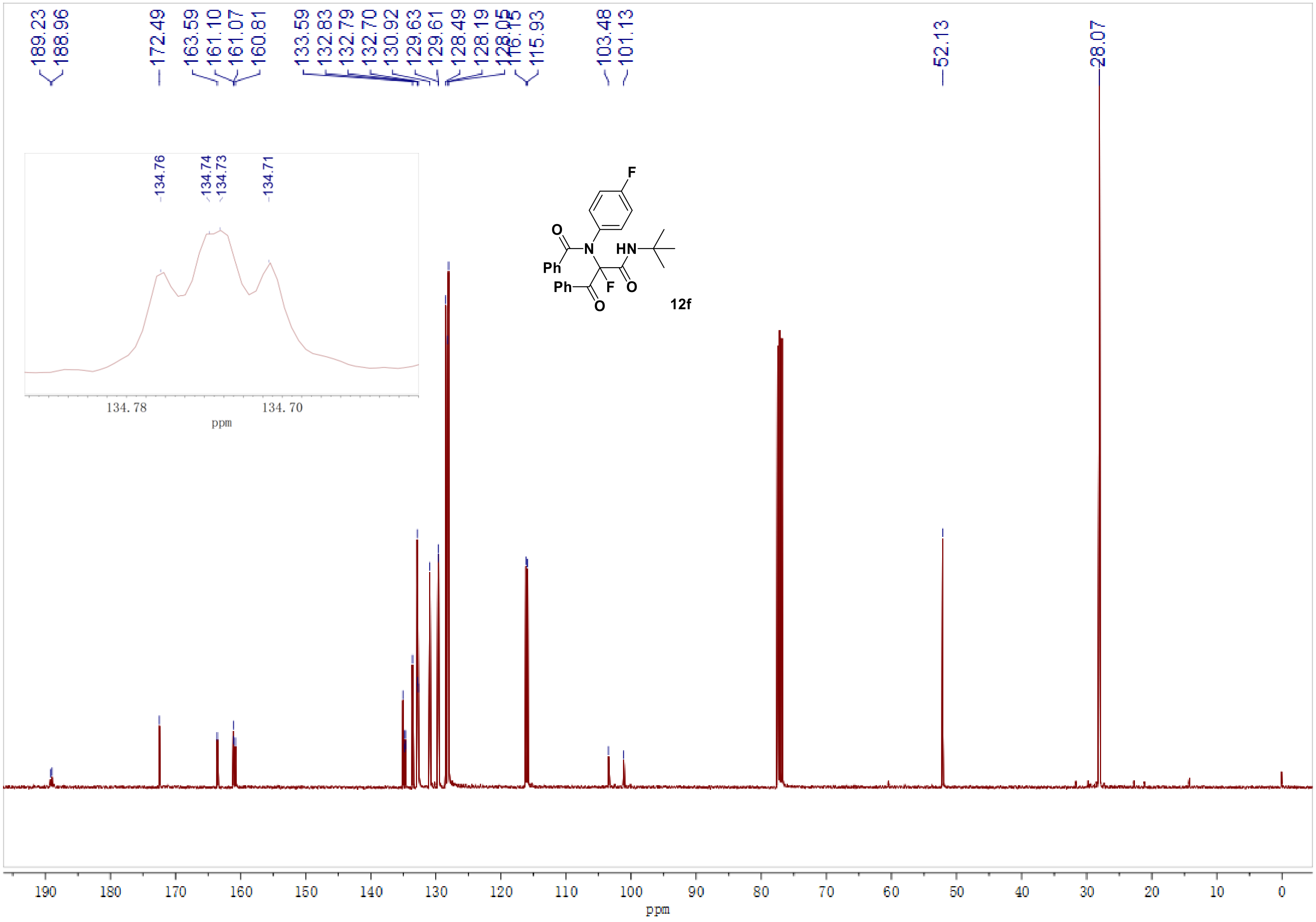
189.23
188.96
172.49
163.59
161.10
161.07
160.81
133.59
132.83
132.79
132.70
130.92
129.63
129.61
128.49
128.19
128.05
116.15
115.93
103.48
101.13
52.13
28.07
134.76
134.74
134.73
134.71
134.78
134.70
ppm
F
O
N
HN
Ph
Ph
F
O
O
12f
190
180
170
160
150
140
130
120
110
100
90
80
70
60
50
40
30
20
10
0
ppm

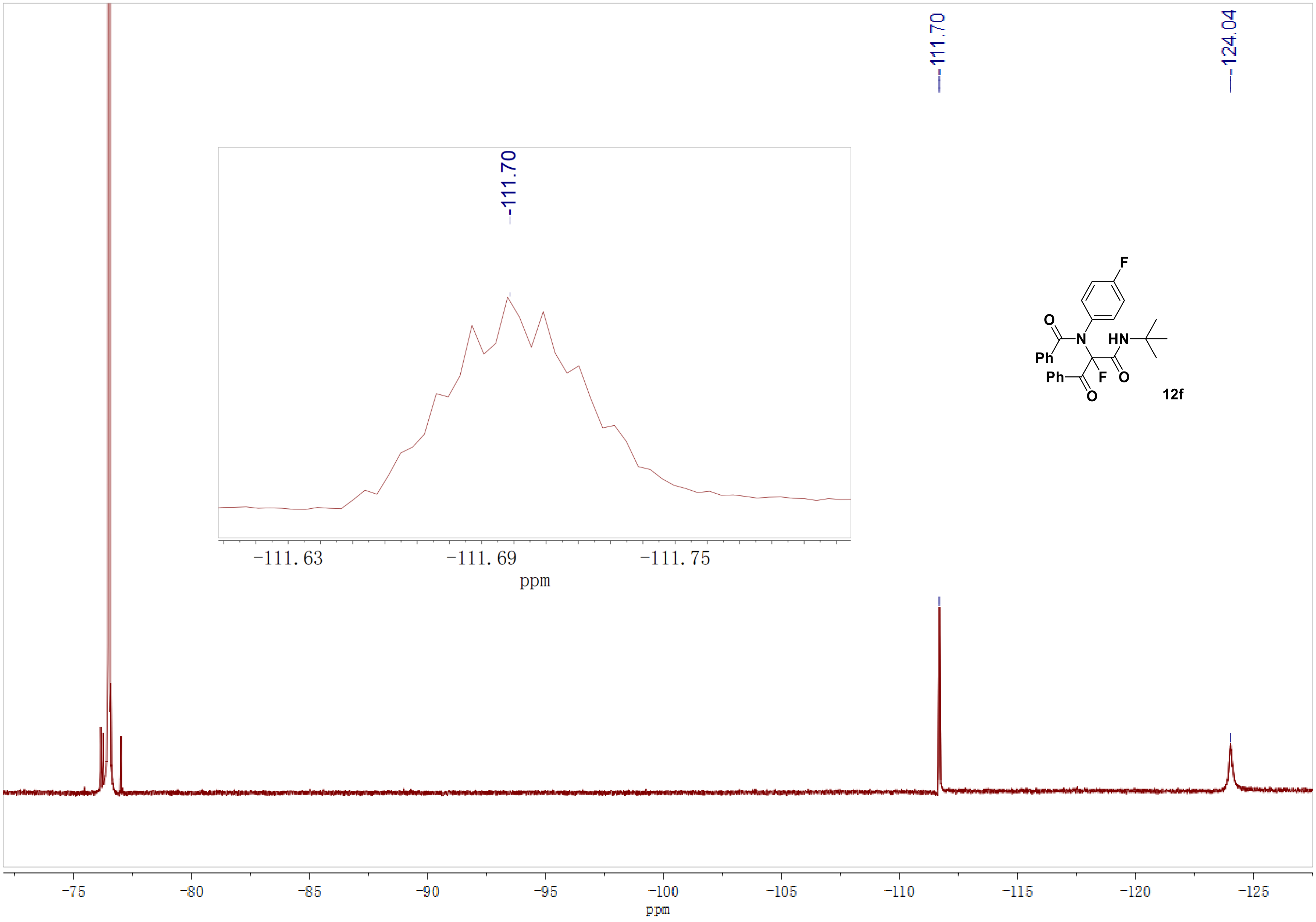
-111.70
-124.04
-111.70
-111.63
-111.69
-111.75
ppm
F
O
N
HN
Ph
Ph
F
O
O
12f
-75
-80
-85
-90
-95
-100
-105
-110
-115
-120
-125
ppm

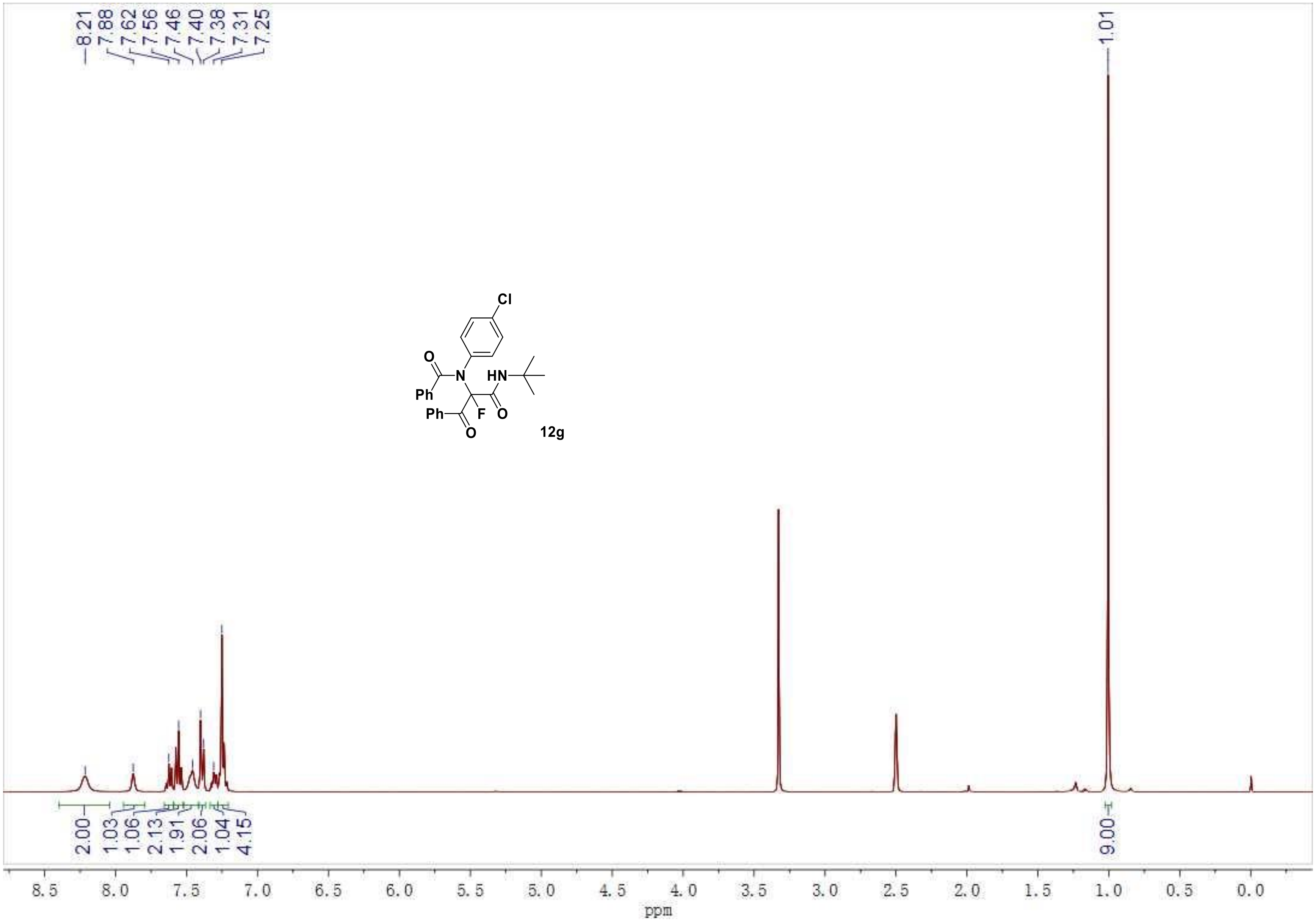
8.21
7.88
7.62
7.56
7.46
7.40
7.38
7.31
7.25
1.01
Cl
O
N
HN
Ph
Ph
F
O
O
12g
2.00
1.03
1.06
2.13
1.91
2.06
1.04
4.15
9.00
8.5
8.0
7.5
7.0
6.5
6.0
5.5
5.0
4.5
4.0
3.5
3.0
2.5
2.0
1.5
1.0
0.5
0.0
ppm

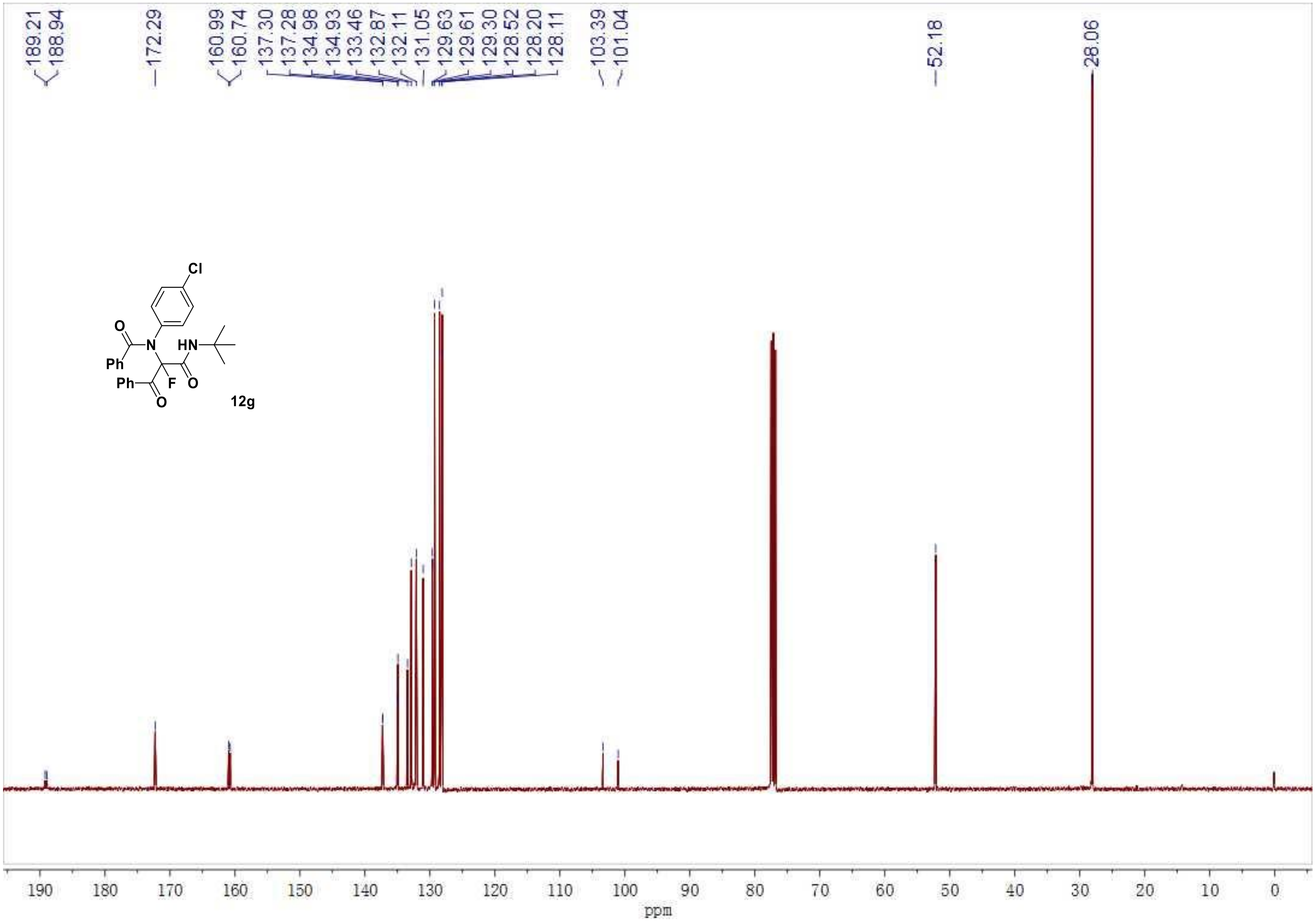
189.21
188.94
172.29
160.99
160.74
137.30
137.28
134.98
134.93
133.46
132.87
132.11
131.05
129.63
129.61
129.30
128.52
128.20
128.11
103.39
101.04
52.18
28.06
Cl
O
N
HN
Ph
Ph
F
O
O
12g
190
180
170
160
150
140
130
120
110
100
90
80
70
60
50
40
30
20
10
0
ppm

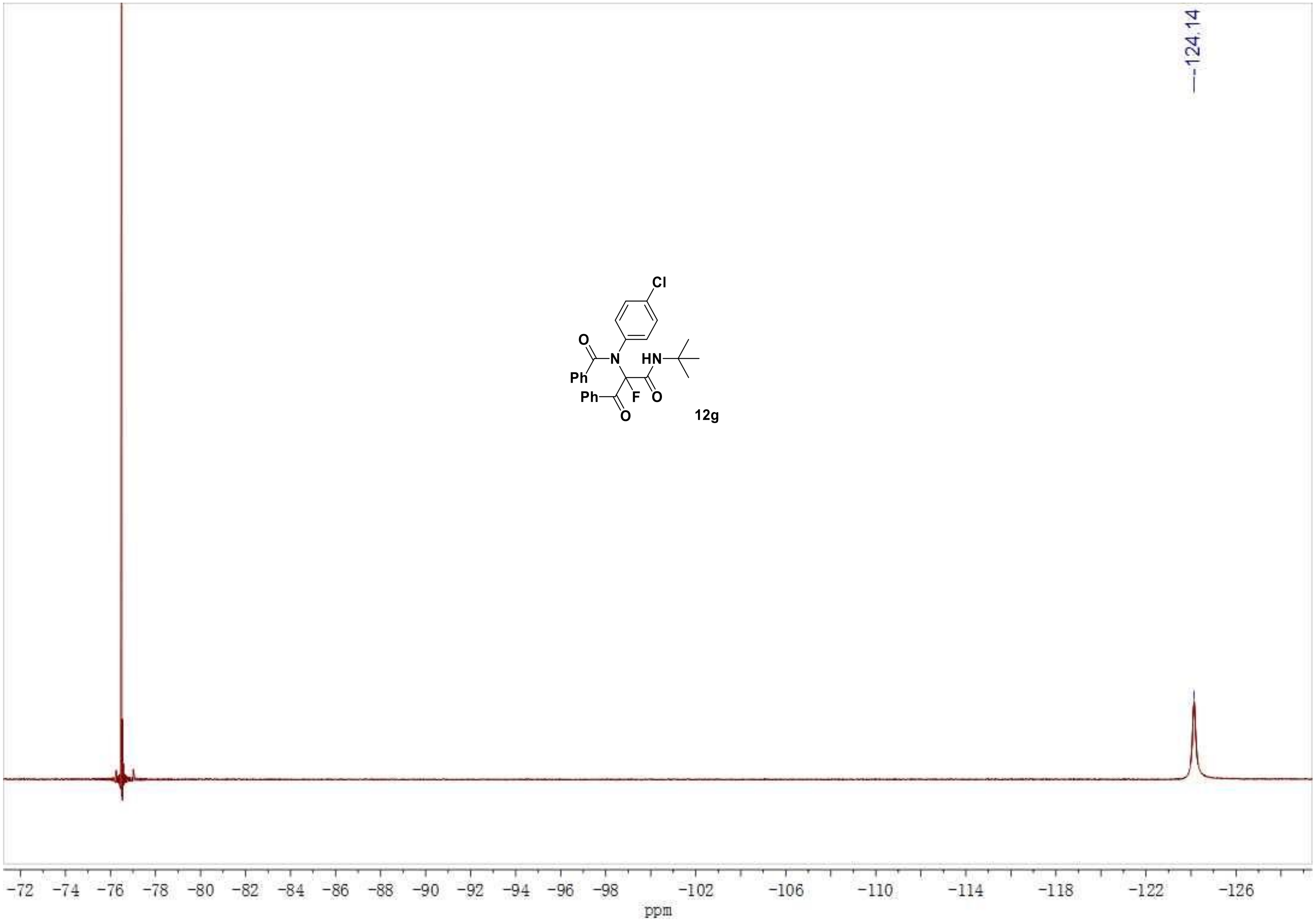

-124.14
Cl
O
N
HN
Ph
Ph
F
O
O
12g
-72 -74 -76 -78 -80 -82 -84 -86 -88 -90 -92 -94 -96 -98 -102 -106 -110 -114 -118 -122 -126
ppm

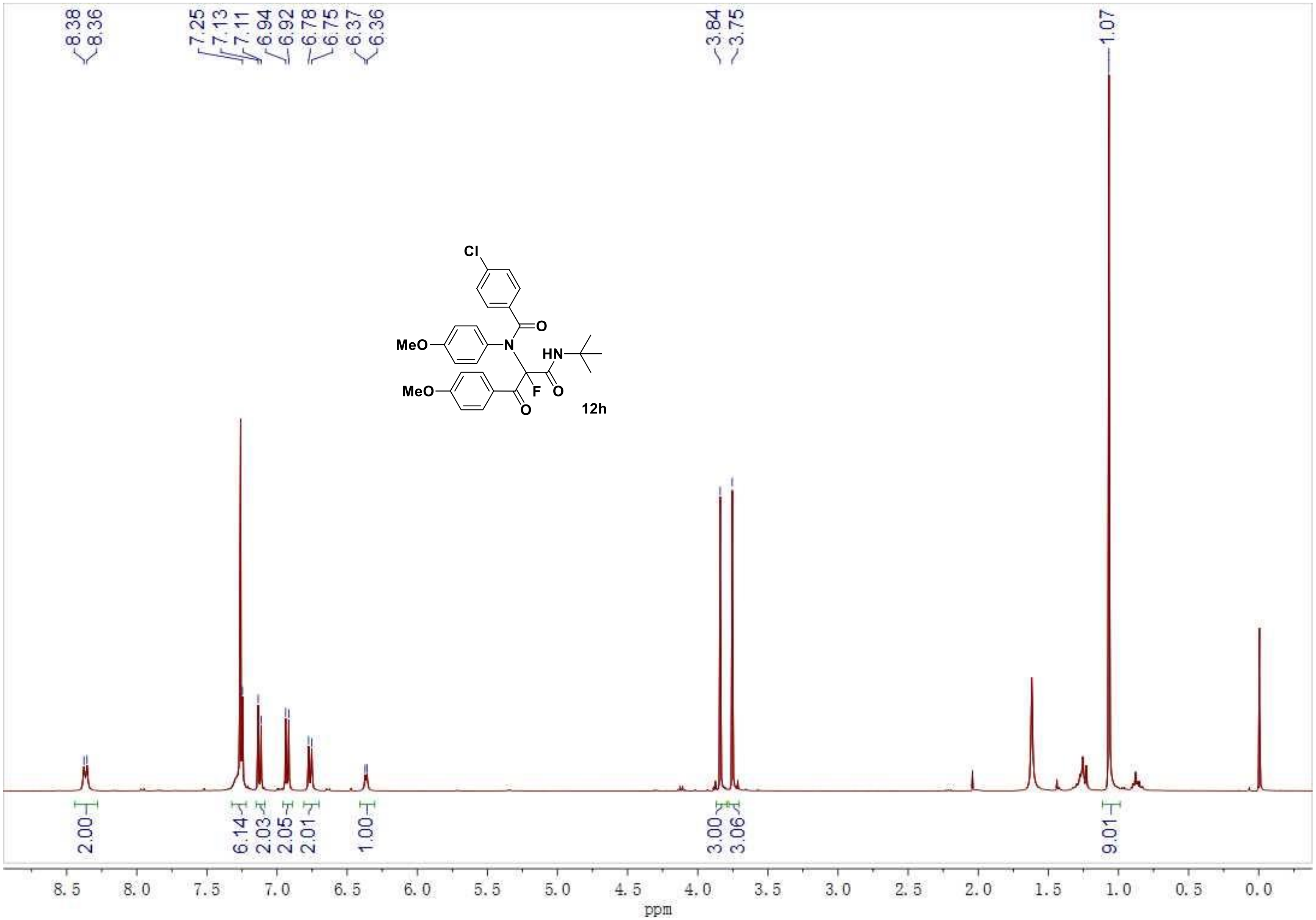

8.38
8.36
7.25
7.13
7.11
6.94
6.92
6.78
6.75
6.37
6.36
3.84
3.75
1.07
Cl
O
MeO
N
HN
MeO
F
O
O
12h
2.00
6.14
2.03
2.05
2.01
1.00
3.00
3.06
9.01
8.5
8.0
7.5
7.0
6.5
6.0
5.5
5.0
4.5
4.0
3.5
3.0
2.5
2.0
1.5
1.0
0.5
0.0
ppm

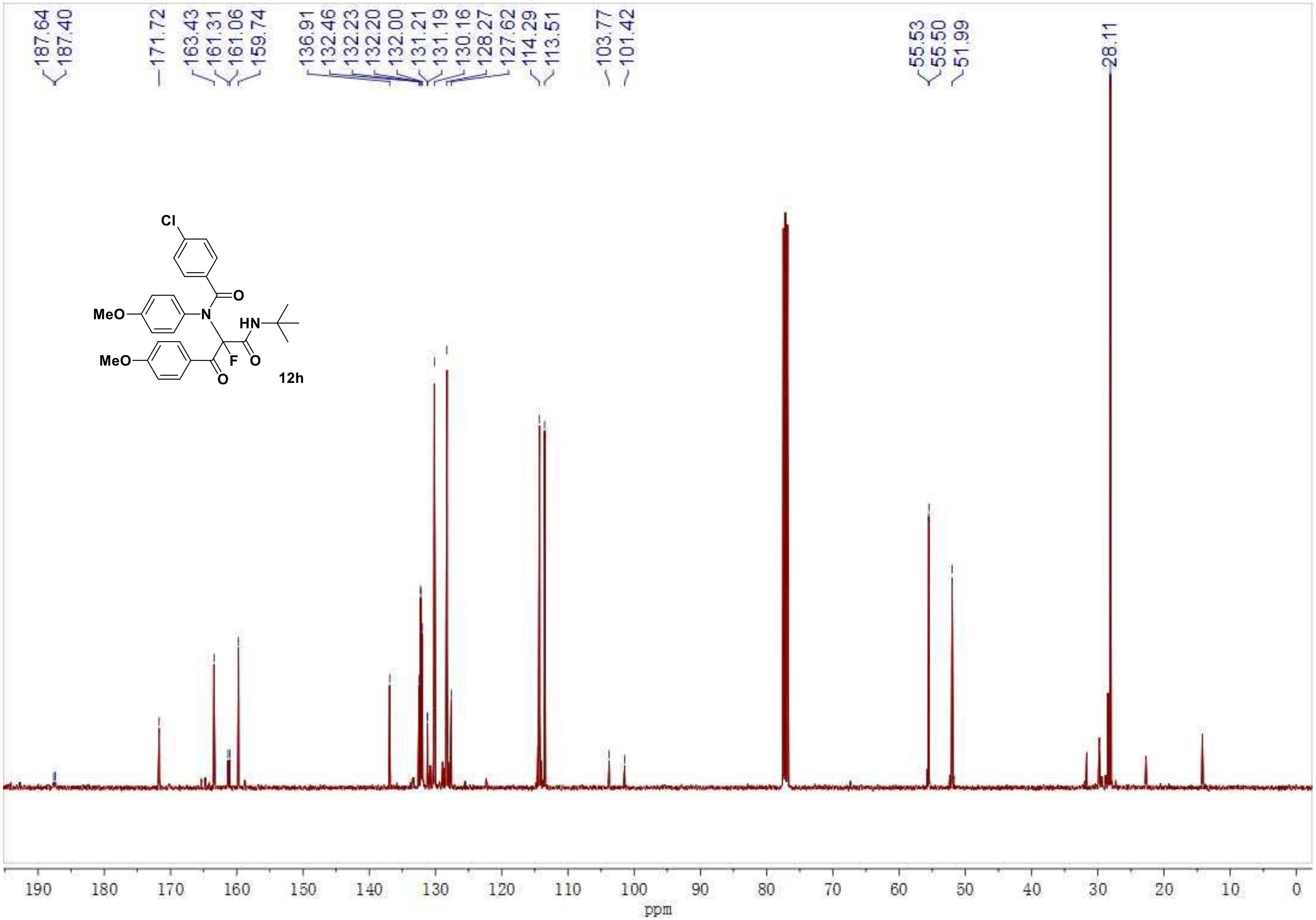
187.64
187.40
171.72
163.43
161.31
161.06
159.74
136.91
132.46
132.23
132.20
132.00
131.21
131.19
130.16
128.27
127.62
114.29
113.51
103.77
101.42
55.53
55.50
51.99
28.11
Cl
MeO
N
O
HN
MeO
F
O
O
12h
190 180 170 160 150 140 130 120 110 100 90 80 70 60 50 40 30 20 10 0
ppm

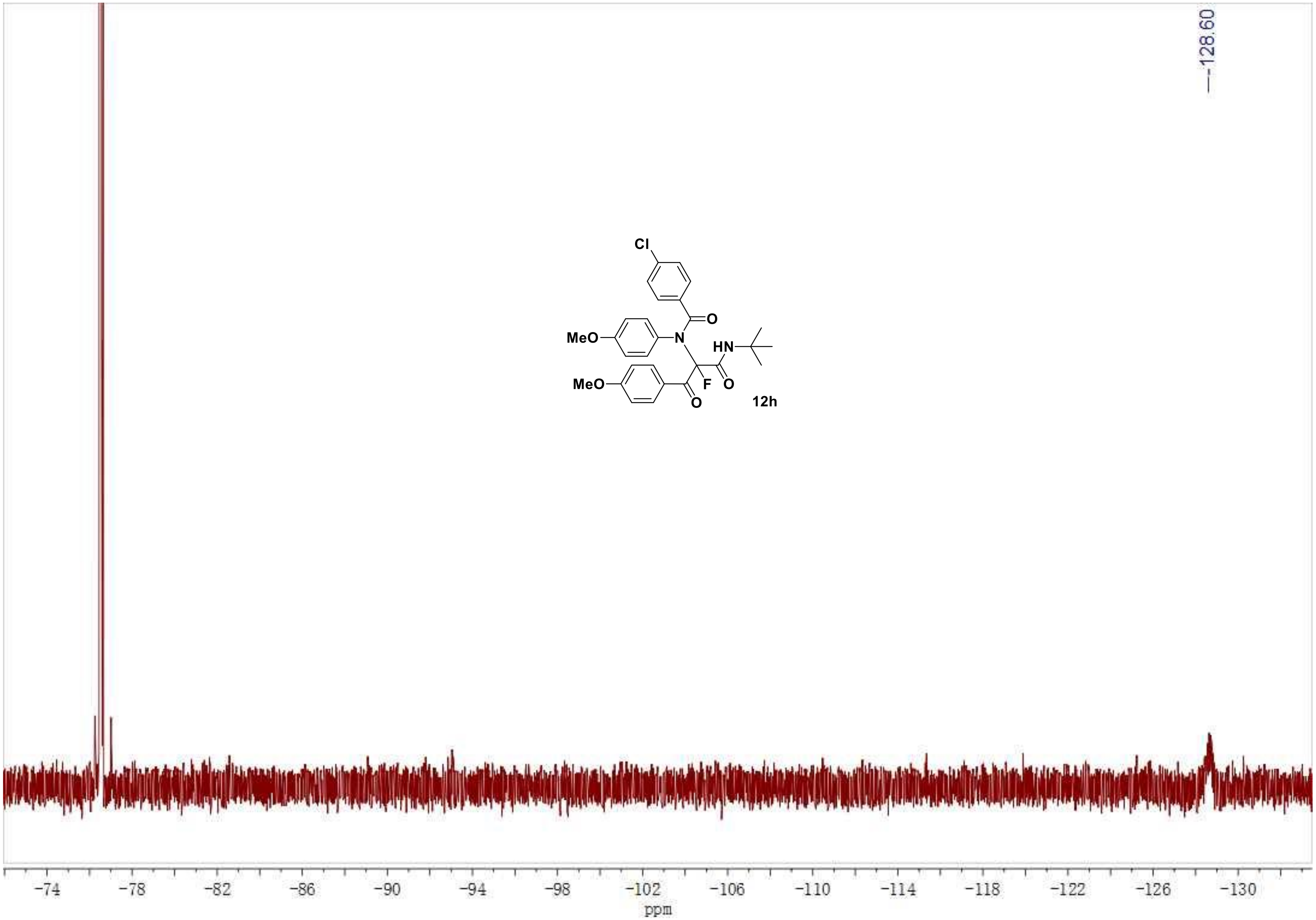

-128.60
Cl
O
MeO
N
HN
F
O
MeO
O
-74
-78
-82
-86
-90
-94
-98
-102
-106
-110
-114
-118
-122
-126
-130
ppm

12h

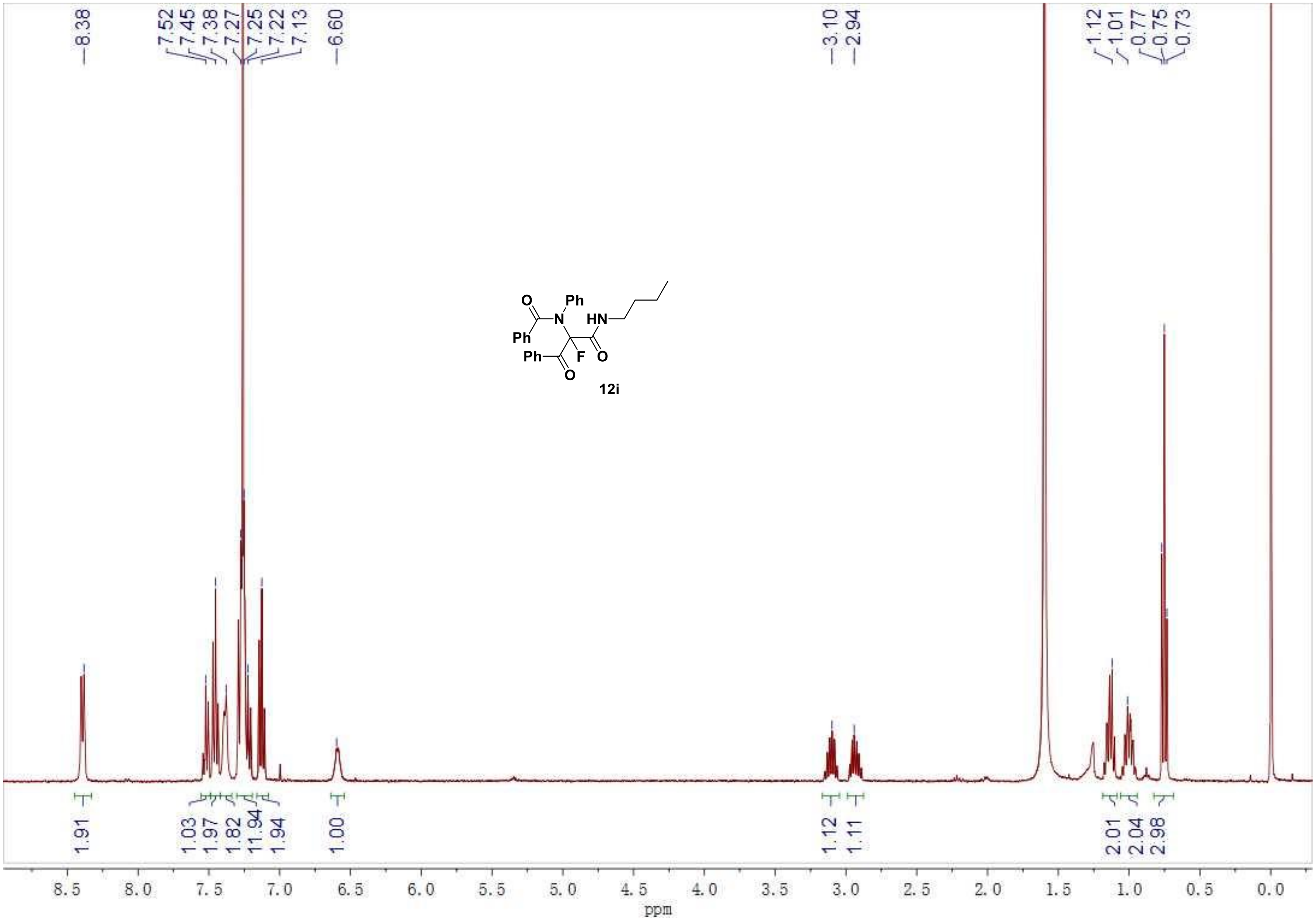

8.38
7.52
7.45
7.38
7.27
7.25
7.22
7.13
6.60
3.10
2.94
1.12
1.01
0.77
0.75
0.73
O
Ph
N
HN
Ph
Ph
F
O
O
12i
1.91
1.03
1.97
1.82
11.94
1.94
1.00
1.12
1.11
2.01
2.04
2.98
8.5
8.0
7.5
7.0
6.5
6.0
5.5
5.0
4.5
4.0
3.5
3.0
2.5
2.0
1.5
1.0
0.5
0.0
ppm

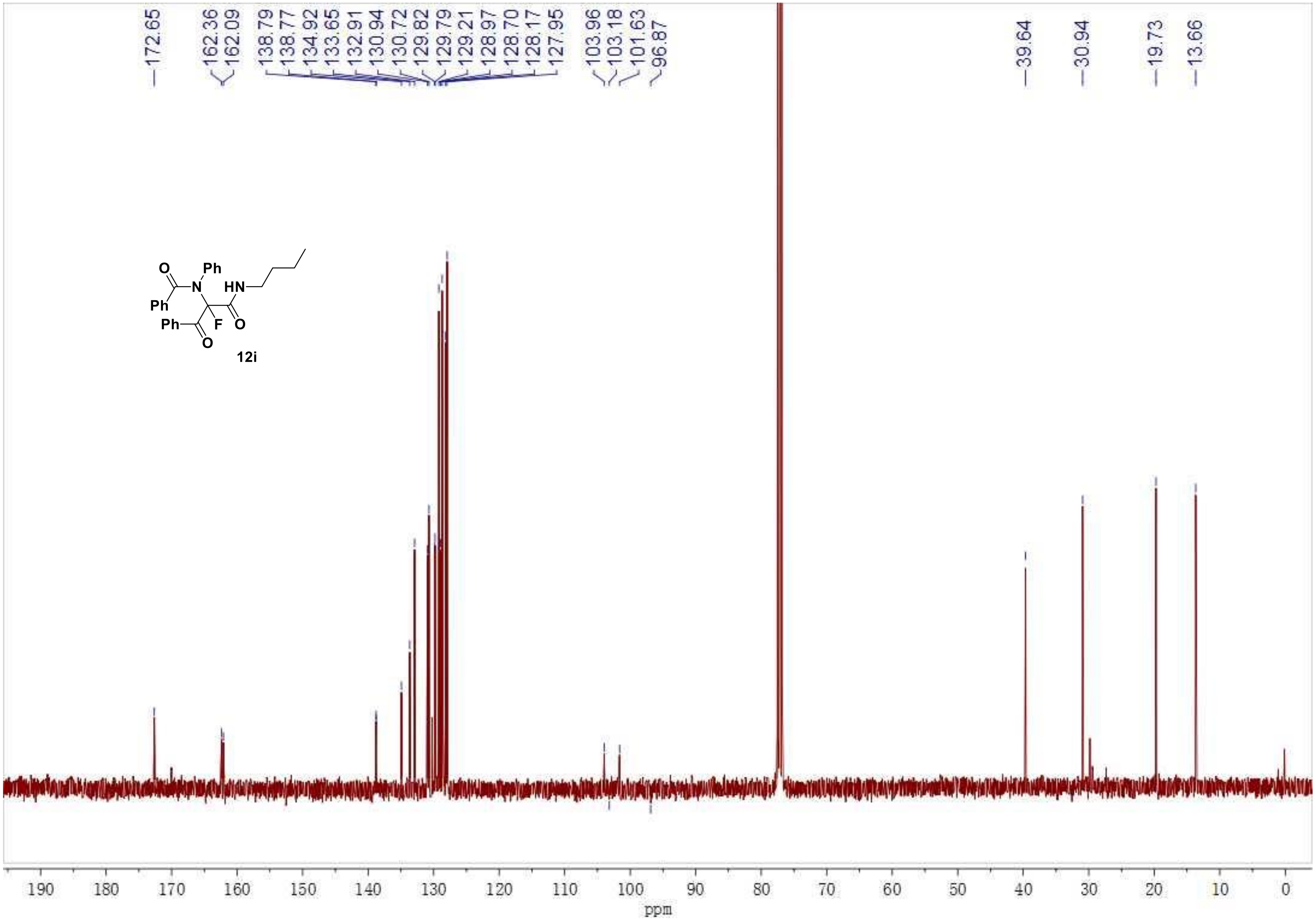
172.65
162.36
162.09
138.79
138.77
134.92
133.65
132.91
130.94
130.72
129.82
129.79
129.21
128.97
128.70
128.17
127.95
103.96
103.18
101.63
96.87
39.64
30.94
19.73
13.66
O
Ph
N
HN
Ph
Ph
F
O
O
12i
190
180
170
160
150
140
130
120
110
100
90
80
70
60
50
40
30
20
10
0
ppm

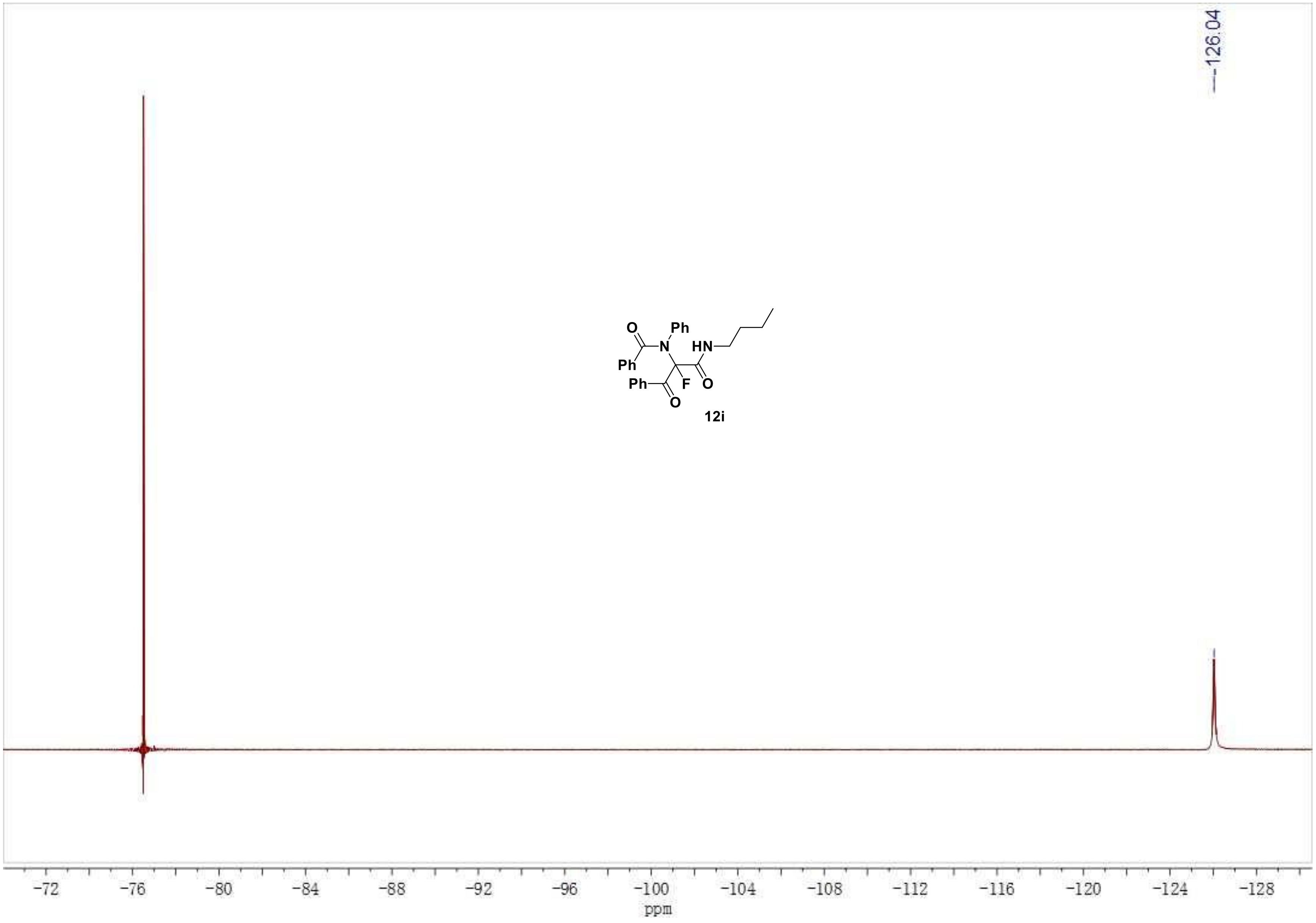

-126.04
O
Ph
N
HN
Ph
Ph
F
O
O
12i
-72
-76
-80
-84
-88
-92
-96
-100
-104
-108
-112
-116
-120
-124
-128
ppm
